# Re-adenylation by TENT5A enhances efficacy of SARS-CoV-2 mRNA vaccines

**DOI:** 10.1038/s41586-025-08842-1

**Published:** 2025-04-16

**Authors:** Paweł S. Krawczyk, Michał Mazur, Wiktoria Orzeł, Olga Gewartowska, Sebastian Jeleń, Wiktor Antczak, Karolina Kasztelan, Aleksandra Brouze, Katarzyna Matylla-Kulińska, Natalia Gumińska, Bartosz Tarkowski, Ewelina P. Owczarek, Kamila Affek, Paweł Turowski, Agnieszka Tudek, Małgorzata Sroka, Tomasz Śpiewla, Monika Kusio-Kobiałka, Aleksandra Wesołowska, Dominika Nowis, Jakub Golab, Joanna Kowalska, Jacek Jemielity, Andrzej Dziembowski, Seweryn Mroczek

**Affiliations:** 1 Laboratory of RNA Biology, International Institute of Molecular and Cell Biology, Warsaw, Poland; 2 Institute of Genetics and Biotechnology, Faculty of Biology, University of Warsaw, Warsaw, Poland; 3 Genome Engineering Facility, International Institute of Molecular and Cell Biology, Warsaw, Poland; 4 ExploRNA Therapeutics, Warsaw, Poland; 5 Institute of Biochemistry and Biophysics, Warsaw, Poland; 6 Laboratory of Protein Structure, International Institute of Molecular and Cell Biology, Warsaw, Poland; 7 Faculty of Physics, University of Warsaw, Warsaw, Poland; 8 Department of Medical Biology, Medical University of Warsaw, Warsaw, Poland; 9 Laboratory of Experimental Medicine, Medical University of Warsaw, Warsaw, Poland; 10 Department of Immunology, Medical University of Warsaw, Warsaw, Poland; 11 Centre of New Technologies, University of Warsaw, Warsaw, Poland; 12 Department of Embryology, Faculty of Biology, University of Warsaw, Warsaw, Poland

**Keywords:** RNA vaccines, RNA metabolism

## Abstract

Despite the widespread use of mRNA vaccines against COVID-19, little is known about the metabolism of therapeutic RNAs. Here we use nanopore sequencing^1–3^ to analyse individual therapeutic mRNA molecules, focusing on their poly(A) tails. We show that the Moderna mRNA-1273 vaccine^4^ has a poly(A) tail of around 100 nucleotides, followed by an mΨCmΨAG sequence. In cell lines, mRNA-1273 undergoes rapid degradation initiated by mΨCmΨAG removal, followed by CCR4–NOT-mediated deadenylation. However, in medically relevant preclinical models, particularly in macrophages, mRNA-1273 poly(A) tails are extended to up to 200 nucleotides by the TENT5A poly(A) polymerase^5–7^, which is induced by the vaccine. Re-adenylation, which stabilizes target mRNAs, is consistently observed in synthetic mRNAs that encode proteins targeted to the endoplasmic reticulum, such as ovalbumin or antigens from Zika virus^8^ or the malaria parasite^9^. The extent of re-adenylation varies: the BioNTech–Pfizer BNT162b2 vaccine^10^ shows less potent re-adenylation than mRNA-1273, which correlates with a smaller proportion of membrane-associated BNT162b2. This highlights the crucial role of spatial accessibility to ER-resident TENT5A in determining re-adenylation efficiency. In vivo, TENT5A is expressed in immune cells that take up mRNA vaccine, and TENT5A deficiency reduces specific immunoglobulin production for mRNA vaccines after immunization in mice. Overall, our findings reveal a principle for enhancing the efficacy of therapeutic mRNAs, paving the way for improvement.

## Abstract (web summary)

Upon intramuscular administration, COVID-19 mRNA vaccines are primarily taken up by macrophages, in which the cellular machinery extends their poly(A) tails, thereby increasing mRNA stability and translation, providing an explanation for the efficacy of these vaccines.

## Main

During the COVID-19 pandemic, mRNA vaccines were deployed on a global scale with unprecedented speed. Despite the need for immediate action, the mRNA vaccine field is still in its early stages, because this method of vaccine design was entirely new. The two anti-COVID-19 mRNA vaccines that were first approved—BNT162b2 (Comirnaty) and mRNA-1273 (Spikevax)—are in vitro transcribed (IVT) mRNAs^4,10^ that encode the coronaviral spike protein. These therapeutic mRNAs have all of their uridines replaced by *N*^1^-methyl-pseudouridines (mΨ), and are overall very similar. However, they differ in their cap structures, untranslated region (UTR) sequences and 3′ tails^11^. The BNT162b2 vaccine is capped cotranscriptionally with a trinucleotide Cap1 analogue (CleanCap AG, with 3′-O-methylation on m7G) and a composite poly(A) tail of 30 adenosines followed by 10 other nucleotides and then 70 extra adenosines (ref. ^12^). The mRNA-1273 vaccine is capped post-transcriptionally, creating natural Cap1 through vaccinia virus capping enzyme (VCE) and mRNA Cap 2′-*O*-methyltransferase (MTA), and has a poly(A) tail of undisclosed length. Both vaccines are encapsulated in lipid nanoparticles (LNPs) of slightly different compositions and enter the cells through endocytic pathways^13,14^.

Intramuscularly administered mRNA vaccines can locally transfect resident and infiltrating immune cells, such as antigen-presenting dendritic cells (DCs) and macrophages. These cells become activated and can migrate to neighbouring lymph nodes^15–18^. LNPs, as well as secreted antigens produced in muscle, are transported to local lymph nodes, where they are taken up by macrophages and resident DCs^18,19^. DCs, which have a central role in adaptive immunity, endocytose antigens produced by other cells and are thought to translate vaccine mRNAs^19^. Peptide antigens are presented by class I and class II major histocompatibility complex (MHC) molecules to induce an immune response^18^. Studies on mRNA vaccines have focused on DCs^20,21^; macrophages and monocytes—despite being the main cell types that take up LNP-encapsulated RNA—have received less attention^15,18,22^. Moreover, the distribution of mRNA vaccines has been studied in local lymph nodes, rather than in muscles, where it is administered. Thus, the role of macrophages and local antigen production in mRNA vaccine efficacy is not well defined. Researchers’ knowledge of the in vivo and intracellular metabolism of mRNA vaccines is also limited. Notably, almost nothing is known about the metabolism of vaccine poly(A) tails, but the paradigm extrapolated from data on endogenous transcripts (or reporters) asserts that the stability of therapeutic mRNAs is determined by the rate of poly(A) tail shortening (deadenylation).

Here we use nanopore RNA sequencing (RNA-seq) to analyse mRNA therapeutics, revealing the complexity of their metabolism. Specifically, we show that the endoplasmic reticulum (ER)-associated TENT5A poly(A) polymerase stabilizes IVT mRNAs encoding secreted proteins. In vivo, TENT5A enhances the immunogenicity of the anti-COVID-19 mRNA-based vaccines mRNA-1273 and BNT162b2, but not that of Nuvaxovid, which contains purified spike protein as an antigen. To our knowledge, the effect of endogenous polyadenylation machinery on mRNA therapeutics has not been previously reported or even considered.

## Nanopore sequencing of mRNA vaccines

**Fig. 1:**
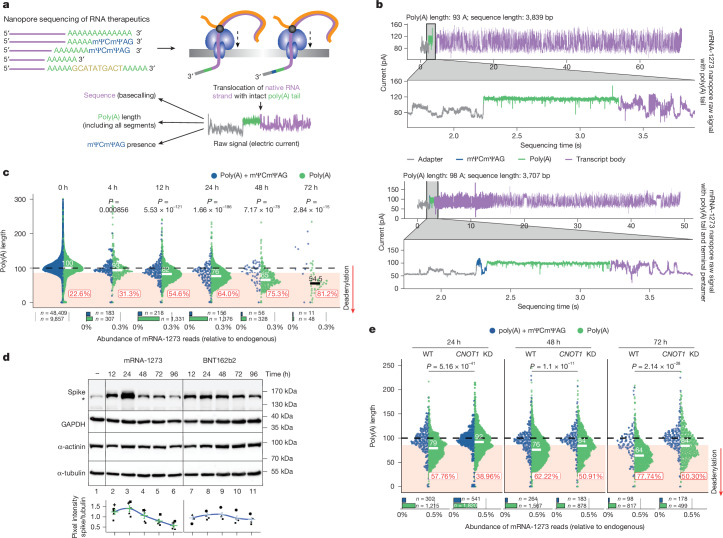
DRS of mRNA-1273 reveals the CCR4–NOT-mediated dynamics of its poly(A) tail in model cell lines. **a**, Overview of the eDRS approach for studying mRNA therapeutics. RNA molecules passing through the pores from the 3′ end cause perturbations in the electric current readout, which are further translated into the sequence (basecalled), and then used to calculate the poly(A) length or to detect the presence of a terminal mΨCmΨAG pentamer. **b**, Representative raw signals from eDRS showing mRNA-1273 with (bottom) and without (top) a 3′ terminal mΨCmΨAG pentamer. The signal from poly(A) is zoomed in for visualization purposes. **c**, mRNA-1273 poly(A) length distribution in HEK293T cells treated with the vaccine for up to 72 h. **d**, Western blot analysis of spike protein expression from mRNA-1273 and BNT162b2 in HEK293T cells up to 96 h after the delivery of mRNA LNPs. Levels of α-actinin, GAPDH and α-tubulin served as loading controls. Asterisk indicates unspecific signal from untreated cells. Bottom, quantification of spike protein levels (five independent biological experiments); mean ± s.e.m. indicated in colour. **e**, mRNA-1273 poly(A) length distribution for HEK293T cells with tetracycline-induced depletion of CNOT1 (*CNOT1* knockdown (KD)) or controls (wild type (WT), without tetracycline), treated with the vaccine for up to 72 h. For **c**,**e**, eDRS-measured poly(A) tail lengths are shown with reads containing (blue) or lacking (green) an mΨCmΨAG sequence, with median values and the fraction of shortened tails shown. Bottom panels show the relative abundance of each group, with group sizes indicated. *P* values calculated with two-sided Wilcoxon test (on reads lacking mΨCmΨAG) with Benjamini–Hochberg correction. Data represent two independent experiments. See also Extended Data Fig. 1. Source data

Owing to methodological challenges, the in vivo metabolism of therapeutic mRNAs is poorly understood. Commonly used sequencing-by-synthesis methods rely on the analysis of PCR-amplified cDNA, but these approaches are indirect and error-prone, particularly when studying poly(A) tails, because of polymerase slippages on homopolymers^1^. Quality control of mRNA therapeutics, especially their poly(A) tails, presents similar difficulties. Direct RNA sequencing (DRS), developed by Oxford Nanopore Technologies (ONT)^2^, is a promising solution to these challenges. DRS records electric currents during the passage of individual single-stranded RNA molecules through a protein pore, providing reliable information about RNA composition with single-molecule resolution (Fig. 1a).

**Extended Data Fig. 1:**
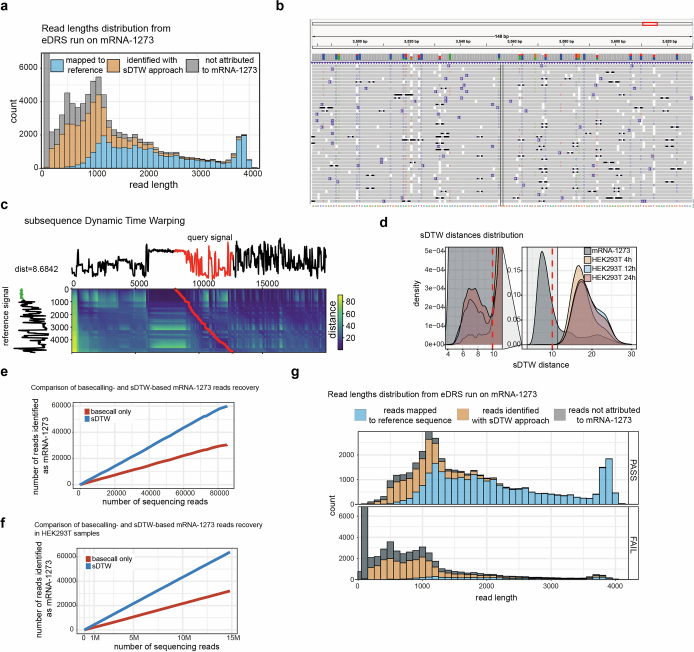
Nanopore DRS of mRNA vaccines. Related to Fig. 1. **a**, Read length distribution of eDRS data. Blue – reads mapped to mRNA-1273 after standard basecalling by Guppy. Orange – reads identified as mRNA-1273 using an sDTW approach. Grey – reads not attributed to mRNA-1273 (too low quality). Bin width = 100, stacked histogram. **b**, IGV snapshot of Guppy basecalled mRNA-1273. Mismatches are visible in positions where mΨ was incorporated. **c**, sDTW approach. Reference signal (on the left, with the part of poly(A) tail marked in green) is compared with the query signal (on top), and a subsequence match location is found (marked in red). Heat map shows the similarities between different parts of reference and query signals, used for drawing the warping path (in red). **d**, Distribution of sDTW distances (compared to reference signal) of crude mRNA-1273 sample, and HEK293T samples incubated with mRNA-1273 (replicate 1, for clarity only time points 4 h, 12 h, and 24 h are shown). Distance threshold (≤10) for identification of reads as originating from mRNA-1273 indicated as red line. **e**, Comparison of mRNA-1273 recovery using standard basecalling and sDTW approach. **f**, Comparison of mRNA-1273 reads recovery from sequencing data of HEK293 + mRNA-1273 cells, using standard basecalling and sDTW approach. **g**, Distribution of read lengths from mRNA-1273 eDRS, for reads passing quality filter (mean qscore ≥7, bottom), or failing (mean qscore <7, top). In blue - reads basecalled using Guppy and aligned to the reference sequence. In yellow- reads identified as mRNA-1273 using sDTW approach. In grey - other reads. Bin width = 100, stacked histogram. Source data

Analysis of intact mRNA-1273 using a standard DRS pipeline (Fig. 1a) revealed that a substantial proportion of aligned reads covered the full-length vaccine (Extended Data Fig. 1a, blue), indicating the potential for comprehensive analysis of mRNA therapeutics. However, uridine-to-mΨ substitution in mRNA therapeutics affects the current signal recorded, resulting in imprecise translation into the sequence (basecalling) (Extended Data Fig. 1b). Therefore, we developed a subsequence dynamic time warping (sDTW) approach (Supplementary Note 1 and Extended Data Fig. 1c), which enables the identification of twice as many sequences compared with the standard DRS pipeline (Extended Data Fig. 1a,d–g).

**Extended Data Fig. 2:**
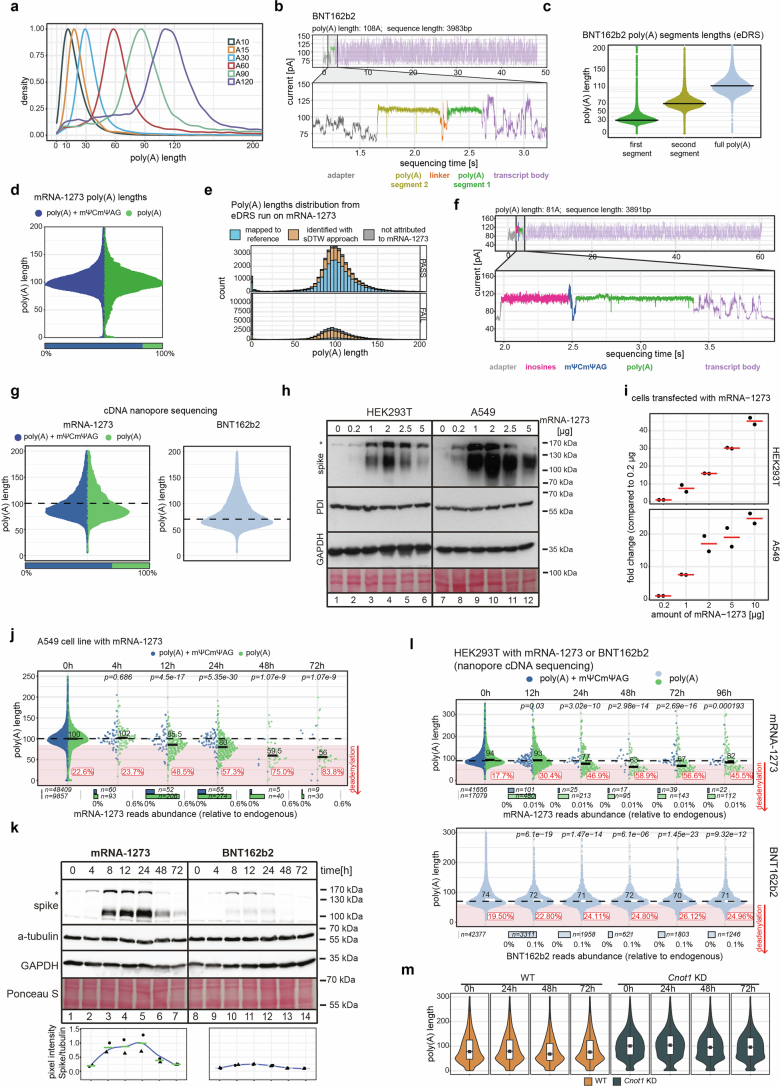
mRNA-1273 terminates with a non-A pentamer (mΨCmΨAG) and its poly(A) tail dynamics are CCR4–NOT-dependent. Related to Fig. 1. **a**, DRS-based poly(A) lengths estimation results for control Renilla luciferase transcripts with predefined poly(A) tails length of 10, 15, 30, 60, 80, and 120 adenines. **b**, Representative raw eDRS signal from BNT162b2. Signal from poly(A) is zoomed in the bottom panel. In violet – transcript body, 2 segments of poly(A) tail are shown in green and khaki, interleaved by 10-nt linker (in orange), in grey – sequencing adaptor. **c**, Distribution of poly(A) lengths for BNT162b2, separately for first (green, 30 adenines) and second poly(A) segment (in khaki, 70 adenines) and whole poly(A) tail (110 nt, in light blue). **d**, Distribution of poly(A) lengths for mRNA-1273 containing (blue) and lacking (green) 3′ terminal mΨCmΨAG. Bottom – fraction of reads in each group is indicated. **e**, Distribution of poly(A) lengths for mRNA-1273 eDRS reads, for reads passing quality filter (mean qscore >=7, bottom), or failing (mean qscore <7, top). In blue- reads basecalled using Guppy and aligned to reference sequence. In yellow - reads identified as mRNA-1273 using sDTW approach. In grey - other reads. Bin width = 4, stacked histogram. **f**, Representative raw eDRS signal from I-tailed mRNA-1273. Signal from poly(A) is zoomed in the bottom panel. Signal from inosines shown in pink, mΨCmΨAG in blue. **g**, Distribution of poly(A) lengths for mRNA-1273 (left) or BNT162b2 (right, second segment, 70 adenines), obtained with nanopore cDNA sequencing. **h**, Western blots for spike protein showing efficient translation of mRNA-1273 in HEK293T and A549 cells 24 h after treatment with varying amounts of vaccine LNPs. PDI and GAPDH levels, and Ponceau staining (bottom) served as loading controls. Asterisk indicates unspecific signal from untreated cells. Representative picture from 2 independent biological experiments shown. **i**, Amounts of vaccine RNA in HEK293T or A549 cells incubated with 0.2-10 µg of mRNA-1273 LNPs per well of 6-well plate for 24 h, assessed with qPCR (n = 2 independent biological experiments). **j**, mRNA-1273 poly(A) length distribution in A549 cells treated with the vaccine LNPs for up to 72 h. **k**, Western blot analysis of spike protein expression from mRNA-1273 and BNT162b2 in HEK293T cells up to 96 h after RNA transfection with lipofectamine-based reagent. Western Blot on spike protein; α-tubulin, GAPDH and Ponceau staining served as loading controls. Asterisk indicates unspecific signal from untreated cells. Bottom, quantification of spike protein levels (2 independent biological experiments). **l**, Poly(A) length distribution for mRNA-1273 (top panel) or BNT162b2 (lower panel) in HEK293T cells treated with the vaccine LNPs for up to 96 h, obtained with nanopore cDNA sequencing. **m**, Distribution of poly(A) lengths of endogenous transcripts in HEK293T cells with tetracycline-induced depletion of *CNOT1* (*CNOT1* KD, shown in black) or controls (WT, without tetracycline, shown in orange) in time course up to 72 h. Boxes show 25th-75th percentiles with median line; whiskers extend to 1.5×interquartile range (IQR). Data for **a**,**b**,**c**,**d**,**e**,**f**,**j** and **m** were obtained with eDRS, for **g** and **l** with cDNA sequencing. For **d**,**g**,**j**, and **l** (top) poly(A) tail lengths are shown with reads containing (blue) or lacking (green) 3′ terminal mΨCmΨAG, with median values and fraction of shortened tails shown. Bottom panels: relative abundance of each group, with group sizes indicated. *P* values calculated with two-sided Wilcoxon test (on reads lacking mΨCmΨAG) with Benjamini–Hochberg correction. Data represent 2 independent biological experiments (**l** and **m**) or single experiment (**j**). Source data

Next, we focused on analysing vaccine poly(A) tails, which are crucial for mRNA stability and translation, using nanopolish polya^23^ (Extended Data Fig. 2a). We confirmed that BNT162b2 exhibits a composite poly(A) tail^12^ (Extended Data Fig. 2b,c). By contrast, mRNA-1273 contains a 100-adenosine poly(A) tract (Extended Data Fig. 2d,e). However, when we visually inspected individual mRNA-1273 nanopore signals, we observed an unexpected perturbation between the poly(A) tail and the adaptor used for library preparation (Fig. 1b, bottom, blue). This signal suggests the presence of terminal non-adenosine residues in the mRNA-1273 vaccine. To determine the specificity of the signature for the 3′ end of mRNA-1273, we used vaccine RNA with an enzymatically extended 3′ end with inosine nucleotides (I-tailing), followed by DRS using a custom protocol. This confirmed the current perturbation at the 3′ end of the vaccine (Extended Data Fig. 2f). Finally, the presence of a TCTAG (mΨCmΨAG in the vaccine mRNA) was revealed through rapid amplification of cDNA 3′ end (3′-RACE) Illumina sequencing. The mΨCmΨAG pentamer is likely to represent the residue of restriction enzyme cleavage of the DNA template. Notably, such pentamer-ending RNAs were efficiently incorporated into the sequencing library, despite the fact that the protocol relies on poly(T)-splint ligation of a sequencing adaptor to the poly(A) tail.

To study mRNA vaccines quantitatively, we developed an enhanced DRS (eDRS) pipeline that includes sDTW and a modified algorithm for detecting poly(A) tails with mΨCmΨAG (Supplementary Note 2 and Supplementary Fig. 1a) or BNT162b2 poly(A) linker sequence (Supplementary Note 3 and Supplementary Fig. 1c). Our analysis of mRNA-1273 revealed that most vaccine RNA molecules had an intact 3′ end, with approximately 20% of reads lacking the mΨCmΨAG sequence (Extended Data Fig. 2d, bottom, green). These also showed a wider poly(A) length distribution (Extended Data Fig. 2d, top, green). Of note, the mΨCmΨAG pentamer enables easy discrimination between intact vaccine mRNA, defined as possessing a 3′-terminal mΨCmΨAG sequence, and processed RNA, which ends with a pure poly(A) sequence; this facilitates detailed analysis of poly(A) metabolism (Supplementary Note 2). A time-efficient computational pipeline that allows the analysis of poly(A) lengths from nanopore cDNA libraries has recently been released by ONT, enabling us to obtain similar poly(A) lengths and to confirm the presence of the pentamer with the orthogonal method (Extended Data Fig. 2g). In summary, both eDRS and cDNA nanopore sequencing are well-suited for analysing mRNA vaccines.

## Deadenylation of mRNA-1273 in cell lines

Next, we used nanopore sequencing to monitor mRNA-1273 stability and poly(A) tail status in cells that are commonly used in preclinical analyses. The optimal amount of vaccine RNA was determined to be non-toxic to cells (Supplementary Note 4 and Supplementary Fig. 2), allowing efficient protein production and detection by eDRS (Extended Data Fig. 2h,i). We then examined mRNA-1273 metabolism in HEK293T and A549 cells in a time course (Fig. 1c and Extended Data Fig. 2j). A clear decrease in mean poly(A) length over time was observed, accompanied by a reduction in the number of vaccine reads. This deadenylation occurred exclusively in reads lacking mΨCmΨAG.

Notably, BNT162b2 exhibited slower antigen production kinetics than mRNA-1273 (Fig. 1d), even when the vaccine mRNA was delivered into cells using lipofectamine reagent (Extended Data Fig. 2k). This was accompanied by a slower rate of deadenylation and greater stability in HEK293T cells, as determined by nanopore cDNA sequencing (Extended Data Fig. 2l).

Finally, to investigate the role of deadenylation in mRNA-1273 decay, we generated a HEK293T cell line with tetracycline-inducible depletion of the scaffold subunit (CNOT1) of the primary deadenylase complex CCR4–NOT. Depletion of CNOT1 resulted in increased global poly(A) lengths of endogenous mRNAs (Extended Data Fig. 2m) and reduced poly(A) tail shortening in mRNA-1273, compared with the uninduced state (Fig. 1e). Significant changes in poly(A) length were observed only in reads that lacked the terminal mΨCmΨAG pentamer. The ratio of transcripts lacking the pentamer remained constant under both conditions, indicating that CCR4–NOT does not participate in mΨCmΨAG removal. These observations suggest that deadenylation restricts the lifespan of therapeutic mRNAs in model cell lines.

## Re-adenylation of mRNA-1273 in vivo

**Fig. 2:**
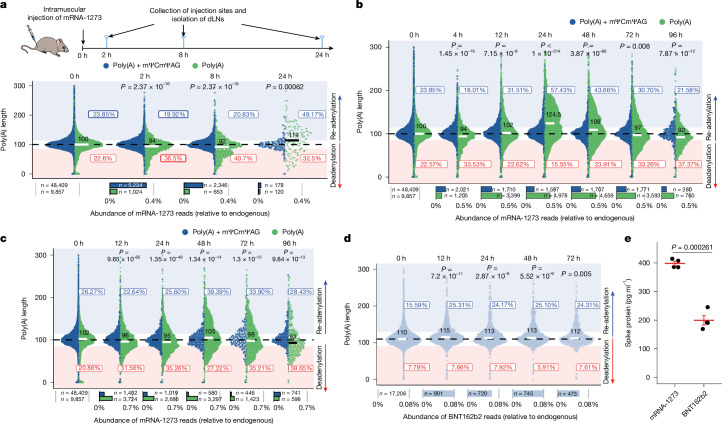
The poly(A) tail of mRNA-1273 is extended in macrophages in vivo and in vitro. **a**, mRNA-1273 poly(A) length distribution in tissues surrounding vaccine injection sites up to 24 h after intramuscular administration. dLNs, draining lymph nodes. **b**,**c**, mRNA-1273 poly(A) length distribution in mBMDMs (**b**) or hMDMs (**c**) incubated with mRNA-1273 LNPs for up to 96 h. **d**, BNT162b2 poly(A) length distribution in mBMDMs incubated with BNT162b2 LNPs for up to 72 h. **e**, In vitro translation assay (3 h) using reticulocyte lysate for mRNA-1273 and BNT162b2. Three micrograms of mRNA was used. Protein was measured by ELISA. Mean ± s.e.m. indicated in red. *P* values calculated with two-sided *t*-test. *n* = 4 independent biological experiments. For **a**–**d**, eDRS-measured poly(A) tail lengths with median values and fractions of shortened or elongated tails are shown. For **a**–**c**, reads containing (blue) or lacking (green) an mΨCmΨAG sequence are shown separately. Bottom panels show the relative abundance of each group, with group sizes indicated. *P* values calculated with two-sided Wilcoxon test (on reads lacking mΨCmΨAG) with Benjamini–Hochberg correction. Data represent two (**a**,**c**,**d**) or three (**b**) independent experiments. See also Extended Data Figs. 2 and 3. Source data

**Extended Data Fig. 3:**
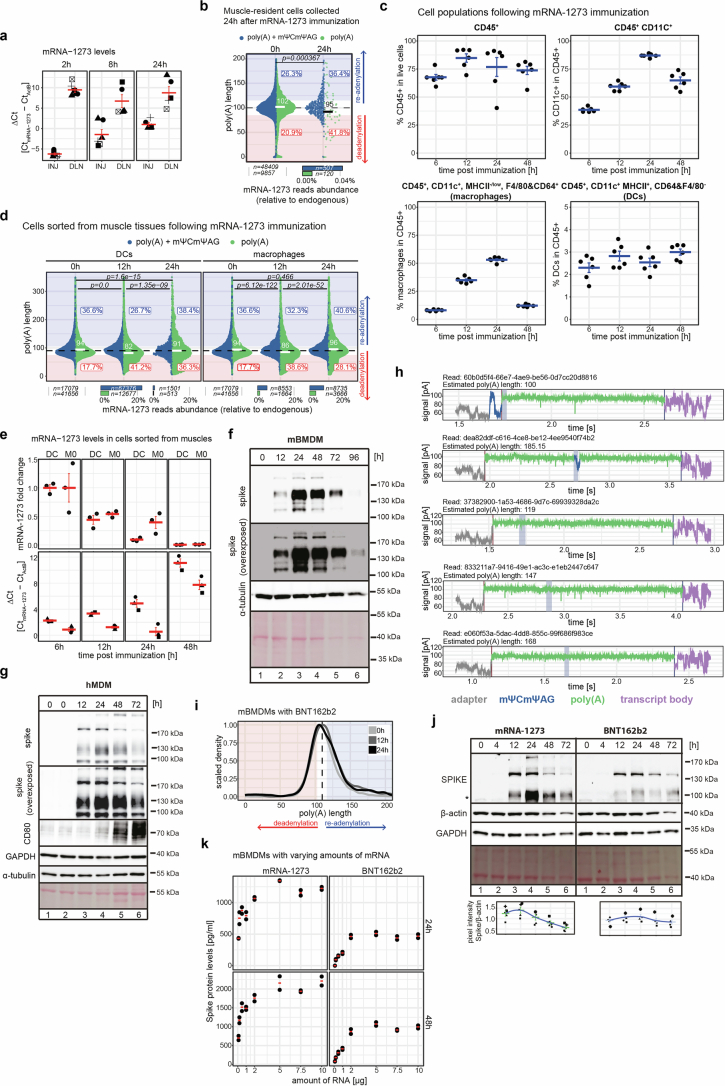
mRNA-1273 poly(A) tail extension in vivo and in macrophages. Related to Fig. 2. **a**, Abundance of mRNA-1273 in tissues surrounding injection sites or in the dLNs, 2 h, 8 h, or 24 h following immunization, expressed as ΔCt (cycle threshold) mRNA-1273 Ct related to ActB Ct values. N = 5 independent biological replicates (mice) for each time point. Values from the same mouse at given time point indicated with matching shapes. Mean values ± s.e.m. indicated in red. **b**, eDRS-based mRNA-1273 poly(A) lengths distribution in muscle-resident cells collected from tissues surrounding injection sites 24 h after immunization with mRNA-1273 vaccine. **c**, Relative abundances of CD45^+^ (top left), CD45^+^ CD11C^+^ (top right), CD45^+^ F4/80&CD64^+^ MHCII^−/low^ CD11c^+^ (macrophages, bottom left), and CD45^+^ CD11c^+^ MHCII^+^ F4/80&CD64^−^ (DCs, bottom right) cell populations in muscles (injection sites) 6 h, 12 h, 24 h and 48 h following immunization with mRNA-1273. Mean values ± s.e.m. indicated in blue. N = 6 independent biological experiments (mice) for each time point. For gating strategy see Supplementary Fig. 5. **d**, cDNA-seq measured mRNA-1273 poly(A) lengths distribution in muscle-resident DCs (CD45^+^, I-A/I-E (MHCII)^high^, CD11c^+^, F4/80^−^ and CD64^−^) and macrophages (CD45^+^, CD11c^+^, F4/80^+^, CD64^+^), sorted from vaccine injection sites 12 h and 24 h after administration. **e**, Levels of mRNA-1273 in muscle-resident macrophages and DCs, sorted out from injection sites 6 h, 12 h, 24 h and 48 h after mRNA-1273 administration. Values are shown as fold change of vaccine molecules per cell, relative to 6 h time point (top) or ΔCt (cycle threshold) mRNA-1273 Ct related to ActB Ct values (bottom). Mean values ± s.e.m. indicated in red. N = 3 independent biological experiments (mice) for each time point. **f**,**g**, Western blot analysis of spike protein expression showing efficient translation of mRNA-1273 in mBMDMs (**f**) or hMDMs (**g**) up to 96 h (**f**) or 72 h (**g**) after mRNA LNPs delivery. Western blot on spike protein, CD80 as the activation marker (**g**), α-tubulin and GAPDH levels and Ponceau S staining (bottom) served as loading controls. Representative pictures from 2 independent biological experiments. **h**, Representative raw DRS currents of mRNA-1273 poly(A) tails detected in samples 24 h after administration to mBMDMs. Unprocessed poly(A) tail with intact pentamer at the poly(A) 3’end (first panel), re-adenylated poly(A) tail with intact pentamer inside poly(A) (second panel), and re-adenylated poly(A) tails without signs of pentamer signature (third, fourth and fifth panel). Expected position of the mΨCmΨAG pentamer signature is marked with grey shading. Out of 100 randomly selected raw nanopore currents, only 2 contained pentamer remnants inside poly(A) stretch. **i**, eDRS-based BNT162b2 poly(A) lengths distribution in mBMDMs 12 h and 24 h after vaccine administration (related to Fig. 2d, 2 time points shown for clarity). Density plot, with values scaled in range 0–1, is shown. **j**, Spike protein production in mBMDMs up to 72 h after mRNA-1273 (left) or BNT162b2 (right) delivery. Western blot on spike protein, GAPDH, β-actin, and Ponceau staining (bottom) served as loading controls. Bottom, quantification of spike protein levels (normalized to β-actin levels, 4 independent biological experiments) ; mean values ± s.e.m. indicated in colour. **k**, Amount of spike protein produced 24 h and 48 h after a dose-dependent administration (0.2-10 µg of mRNA LNPs per well in 6-well plate) of mRNA-1273 (left) or BNT162b2 (right), assessed by ELISA from culture medium. N = 2 independent biological experiments. For **b** and **d**, reads are divided into containing (blue) or lacking (green) mΨCmΨAG sequence, with median values and fractions of elongated or shortened tails shown. Bottom panels: relative abundance of each group, with group sizes indicated. P values calculated with two-sided Wilcoxon test (on reads lacking mΨCmΨAG) with Benjamini–Hochberg correction. Data represent 2 independent biological experiments (**b**). For **d**, sequencing of material pooled from 6 mice was done.

The fate of mRNA therapeutics depends on various factors that are difficult to replicate in model cell lines. The stability of the mRNA and the therapeutic outcome might be influenced by the microenvironment at the RNA delivery site and by specific gene-expression patterns in target cells. To study vaccine mRNA metabolism, we delivered mRNA-1273 intramuscularly in a mouse model. The RNA was isolated directly from the tissues at the injection sites and from the draining lymph nodes at 2 h, 8 h and 24 h after immunization (Fig. 2a). Quantitative PCR with reverse transcription (qRT–PCR) revealed that the abundance of vaccine mRNA is orders of magnitude higher at the injection sites than in the draining lymph nodes (Extended Data Fig. 3a), in which the levels of mRNA-1273 were insufficient for effective eDRS analysis. eDRS on samples from the injection sites revealed that most mRNA-1273 poly(A) tails were unprocessed and contained a terminal mΨCmΨAG sequence even 24 h after injection, although mild deadenylation of processed tails was noticeable at all time points analysed (Fig. 2a). Of note, we found that at the 24-h time point, the median poly(A) tail length of mRNA-1273 increased from 100 to 114 nucleotides, and that 49% of the reads lacking mΨCmΨAG pentamer had lengths greater than 115 nucleotides. We confirmed mRNA-1273 poly(A) tail extension with eDRS on RNA isolated from immune cells infiltrating vaccine injection sites (Extended Data Fig. 3b).

To gain further insight into infiltrating immune cells, which presumably take up mRNA-1273, we performed a flow-cytometry analysis in the time course after immunization (Supplementary Note 5 and Supplementary Figs. 3–6). We observed that 24 h and 48 h after immunization, the muscles were populated almost exclusively by macrophage-type cells (F4/80^+^CD64^+^) also expressing CD11c^+^ (Extended Data Fig. 3c and Supplementary Fig. 6). At the same time, cells with markers attributed to tissue-resident DCs (CD45^+^, I-A/I-E (MHCII)^high^, CD11c^+^, F4/80^−^ and CD64^−^) represented a minor population (around 20 times smaller than the macrophage population), the abundance of which remained stable over time. We then collected these two populations in a post-injection time course. Notably, cDNA nanopore sequencing indicated that mRNA poly(A) tails can be extended in vivo at 24 h when compared with 12 h for both macrophages and DCs (Extended Data Fig. 3d). However, deadenylation dominates in DCs, leading to a sharp decrease in mRNA stability.

In parallel with cDNA sequencing, we assessed the absolute abundance of mRNA-1273 using qRT–PCR. Both DCs and macrophages efficiently take up the vaccine, with an average of 82 and 152 molecules per cell, respectively (Extended Data Fig. 3e). However, macrophages exhibited a greater stability of mRNA-1273 than DCs (Extended Data Fig. 3d,e), which correlates with the more potent re-adenylation. Given the abundance, macrophages represent the main cell population responsible for the local metabolism of vaccine mRNA, and we therefore shifted to in vitro cultures of mouse bone marrow-derived macrophages (mBMDMs) and human monocyte-derived macrophages (hMDMs) as experimental models. Both were efficiently transfected with mRNA-1273 LNPs without visible toxic effects (Supplementary Note 4), allowing antigen detection for up to 96 h (Extended Data Fig. 3f,g). The stability of the vaccine mRNA was observed for at least 72 h, with approximately 25% of the molecules retaining unprocessed 3′ ends after 24 h (Fig. 2b,c). In mBMDMs, we observed a median elongation of around 24 adenosines in the poly(A) tails of vaccine mRNAs 24 h after transfection, with a fraction of tails reaching up to 200 adenosines. The vast majority of the elongated tails lacked the pentamer signature in the raw nanopore currents (Extended Data Fig. 3h). The median poly(A) length returned to the original 100 adenosines 72 h after transfection. In hMDMs, re-adenylation of mRNA-1273 was observed 48 h after vaccine delivery, although to a lesser extent (Fig. 2c). All of this indicates that re-adenylation of mRNA-1273 vaccine observed in vivo can be recapitulated with cultured macrophages as models.

We speculated that a similar phenomenon would be observed for BNT162b2, but in fact we observed only a minor extension of poly(A) tails throughout the time course after administration to mBMDMs (Fig. 2d and Extended Data Fig. 3i). This could be related in part to an inability to distinguish between processed and intact tails, which is not the case for mRNA-1273 (Supplementary Note 2). However, translation efficiency is also diminished in mBMDMs treated with BNT162b2, compared with those treated with mRNA-1273 (Extended Data Fig. 3j). In addition, dose-dependent assays using mBMDMs showed that mRNA-1273 was more effectively translated at all dose ranges—compared with BNT162b2, which produced less antigen (Extended Data Fig. 3k). These slower kinetics of protein production seem to be directly related to the translation efficiency, because the effect is also seen in an vitro translation system based on reticulocyte lysate (Fig. 2e).

In summary, our data show that therapeutic mRNA can undergo poly(A) tail elongation in target cells, as shown for mRNA-1273 in vivo and in cultured macrophages.

## Treatment with mRNA-1273 induces TENT5A

**Fig. 3:**
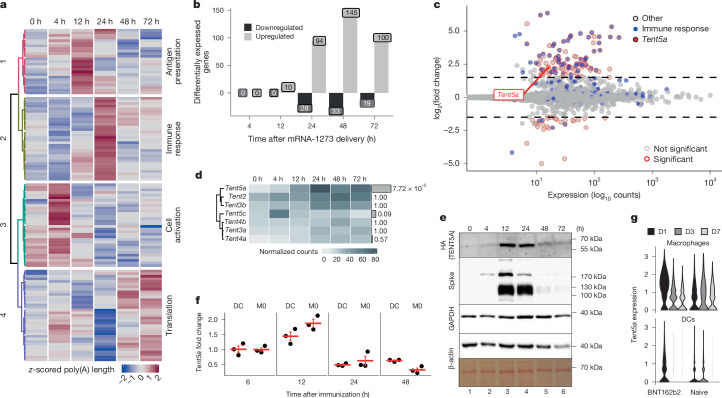
mRNA-1273 induces the innate immune response and the expression of TENT5A. **a**, Transcripts with changed poly(A) lengths (*n* = 124, Kruskal–Wallis test) after administration of mRNA-1273 to mBMDMs. *Z*-score normalized poly(A) lengths are represented in a colour scale. Clustering using hclust, ward.D method. Major functionalities enriched in a given cluster are shown on the right. **b**,**c**, Differential expression analysis after administration of mRNA-1273 to mBMDMs. **b**, Number of upregulated or downregulated genes at time points (compared with 0 h). **c**, MA plot for the 24-h time point; genes with *P* < 0.05, |log_2_(fold change)| ≥ 1.5 are marked with red circles and immune response genes are marked in blue. **d**, Expression (DESeq2-normalized counts) of non-canonical poly(A) and poly(U) polymerases in mBMDMs treated with mRNA-1273 for up to 72 h. Gene names are on the left, adjusted *P* values from the DESeq2 LRT test are on the right, with bar height indicating significance. **e**, Expression of TENT5A protein in mBMDMs derived from a *FKBP12*^*F36V*^*-HA-Tent5a* knock-in mouse line after administration of mRNA-1273. Western blot for HA (to detect tagged TENT5A) and spike; GAPDH, β-actin and Ponceau staining (bottom) served as loading controls. *n* = 1. See Extended Data Fig. 5a. **f**, Levels of *Tent5a* (by qRT–PCR) in muscle-resident macrophages (M0) and DCs, sorted from injection sites 6 h, 12 h, 24 h or 48 h after injection of mRNA-1273. Three independent replicates (mice) per time point. Values shown as fold change relative to the 6-h time point. Mean values ± s.e.m. indicated in red. **g**, *Tent5a* expression (from single-cell RNA-seq) in lymph node macrophages (top) and DCs (bottom) of naive mice or mice immunized with BNT162b2, at days (D) 1, 3 or 7 after immunization. Data reanalysed from a previous study^15^. **a**–**d** show results of the poly(A) lengths (**a**) or differential expression analysis (DESeq2, **b**–**d**) on eDRS sequencing data from three independent biological experiments. See also Extended Data Fig. 4.

**Extended Data Fig. 4:**
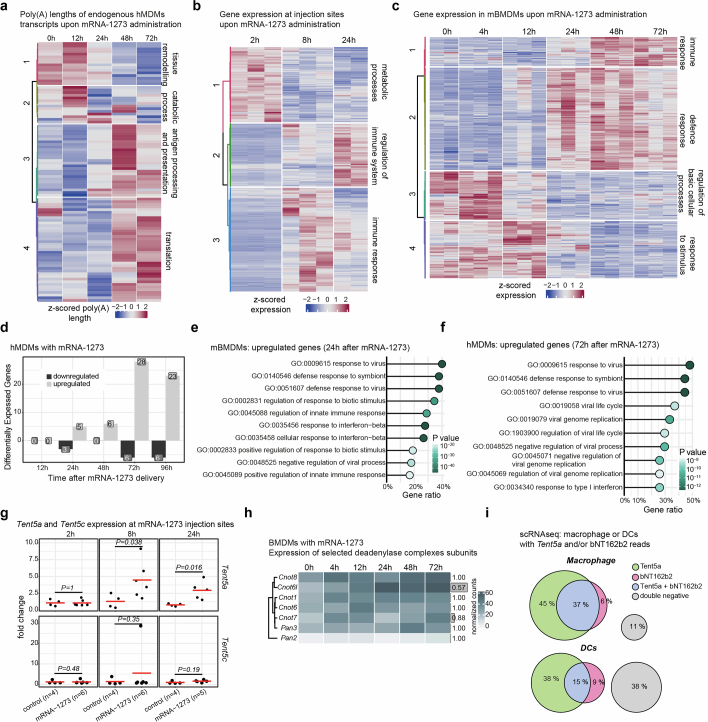
Innate immune response and induction of TENT5A poly(A) polymerase after vaccine administration. Related to Fig. 3. **a**, Transcripts with changed poly(A) lengths (revealed with Kruskal–Wallis test) in the time course (up to 72 h) upon mRNA-1273 LNPs administration to hMDMs. Z-score normalized poly(A) lengths are represented in a colour scale. **b**, Genes with changed expression in tissues surrounding injection sites upon mRNA-1273 administration. Expression values were z-score normalized. Only genes with significant change in expression (DESeq2 LRT test *P* < 0.05) are shown. **c**, Genes with changed expression in mBMDMs upon mRNA-1273 LNPs administration. Expression values were z-score normalized. Only genes with significant change in expression (DESeq2 LRT test *P* < 0.05) are shown. **d**, Differentially expressed genes following administration of mRNA-1273 in hMDMs. Numbers of genes up- and downregulated at each time point (in comparison to time 0 h) are shown. Differential expression analysis done with DESeq2 R package. **e**,**f**, GO (Biological Process) terms significantly enriched for genes upregulated 24 h (**e**) or 72 h (**f**) after mRNA-1273 administration in mBMDMs (**e**) or hMDMs (**f**). **g**, Expression of *Tent5a* (left panel) or *Tent5c* (right panel) at mRNA-1273 injection sites measured with qPCR, presented as fold change (relative to 2 h). P values were calculated with two-sided Wilcoxon test. Mean values indicated in red. Group sizes (number of mice included) provided on the plot. **h**, Expression (DESeq2-normalized counts) of deadenylase complexes subunits in mBMDMs treated with mRNA-1273 LNPs for up to 72 h. Gene names are on the left, adjusted *P* values from the DESeq2 LRT test are shown on the right, with bar height indicating significance. **i**, Cells with detected *Tent5a*, BNT162b2, both, or none of them, in macrophages (top) of DCs (bottom) in the single-cell RNA-seq data from a previous study^15^. For **a**–**c**: Clustering done using hclust, with ward.D method. Descriptions of major functionalities enriched in a given cluster are shown on the right. For **e** and **f**: on the x-axis the percentage of all upregulated genes assigned to given GO term. Colours represent the enrichment *P* value. Data from 2 (**a,b**,**d**,**f**) or 3 (**c**,**e**,**h**) sequencing datasets from independent biological experiments.

Whole-transcriptome analysis of mBMDMs treated with mRNA-1273 revealed significant changes in poly(A) length not only for mRNA-1273 but also for 124 endogenous transcripts (Kruskall–Wallis test, *P* < 0.001). The transcripts associated with immune response showed the most pronounced tail extension, with an average of 20 adenosines at 24 h (Fig. 3a and Supplementary Tables 1 and 2). These included MHC components (*H2-K1*, *H2-D1*, *H2-T23* and *B2m*), serum complement components (*C1qb* and *C1qc*), lysosomal proteins (*Lamp1* and *Laptm5*) and innate immune response genes (*Ctsb*, *Ctsd*, *ApoE*, *Lyz2*, *Cst3* and *Ctss*). Out of the 92 transcripts with a significant change in poly(A) tail length in hMDMs (Extended Data Fig. 4a and Supplementary Table 3), 38 were orthologues of those identified in mBMDMs, encoding mainly proteins that are involved in antigen presentation.

Because poly(A) extension of mRNA-1273 and endogenous transcripts occurred simultaneously in both cell types (after 24 h and 48 h in mBMDMs and hMDMs, respectively), we hypothesized that a common factor—probably transcriptionally induced after vaccine delivery—mediates this process.

Indeed, the changes in poly(A) tails were accompanied by complex transcriptomic responses. At the injection sites, upregulated genes (Extended Data Fig. 4b, clusters 2 and 3 and Supplementary Table 4) were enriched in functions related to the immune response and cell activation (Supplementary Table 5), suggesting the infiltration of immune cells, which is consistent with previous reports^19^. Administration of mRNA-1273 resulted in changes in the expression of 327 and 87 genes in mBMDMs and hMDMs, respectively, of which 37 were orthologous. Differentially expressed genes were observed starting at 24 h after mRNA-1273 administration in mBMDMs (Fig. 3b, Extended Data Fig. 4c and Supplementary Table 6) and after 72 h in hMDMs (Extended Data Fig. 4d and Supplementary Table 7), and continued until the end of the experiment. Of note, the observed responses resembled those previously reported for the BNT162b2 vaccine^15^, with increased expression of immune response genes (Fig. 3c, Extended Data Fig. 4e,f and Supplementary Tables 8 and 9), including several interferon-stimulated genes (ISGs, such as *Stat1*, *Stat2*, *Mx1* and components of the OAS pathway) and genes involved in antigen presentation.

Among the genes upregulated after mRNA-1273 administration in mBMDMs, we observed *Tent5a*, the only non-canonical cytoplasmic poly(A) polymerase exhibiting such a pattern (Fig. 3d). Previously, we showed that *Tent5a* and *Tent5c* were induced in mBMDMs after activation with lipopolysaccharide (LPS)^7^, and we observed an elongation of poly(A) tails in a group of mRNAs; this elongation was very similar to that seen after mRNA-1273 delivery. It suggests that TENT5A is indeed a factor responsible for vaccine re-adenylation. Moreover, *Tent5a* is the only cytoplasmic poly(A) polymerase that is significantly induced at vaccine injection sites (Extended Data Fig. 4g), probably in infiltrating macrophages. *Tent5c* is also expressed in all analysed cells but at a much lower level (Fig. 3d). At the same time, no change in the expression of subunits of the deadenylase complexes was observed (Extended Data Fig. 4h). Finally, the induction of TENT5A after mRNA-1273 administration is also visible at the protein level, as revealed by an analysis of mBMDMs derived from a knock-in mouse line (*FKBP12*^*F36V*^*-HA-Tent5a*) expressing TENT5A fused with a dTAG degrader (FKBP12(F36V)–HA tag) (Fig. 3e). Accordingly, we observed increased expression of *Tent5a* in cells sorted from the injection sites 12 h after immunization (Fig. 3f). One day after immunization with BNT162b2, some induction of *Tent5a* was also observed in single-cell RNA-seq data from a previous study^15^, which we reanalysed focusing on the expression of TENT genes (Fig. 3g). In this analysis, *Tent5a* expression was detected in macrophages (but not in DCs) taking up the vaccine (Extended Data Fig. 4i).

Overall, these experiments suggest a possible role for TENT5A and, to a lesser extent, TENT5C, in the re-adenylation of mRNA-1273 observed in macrophages, in both mouse and human models.

## TENT5A re-adenylates mRNA-1273

**Fig. 4:**
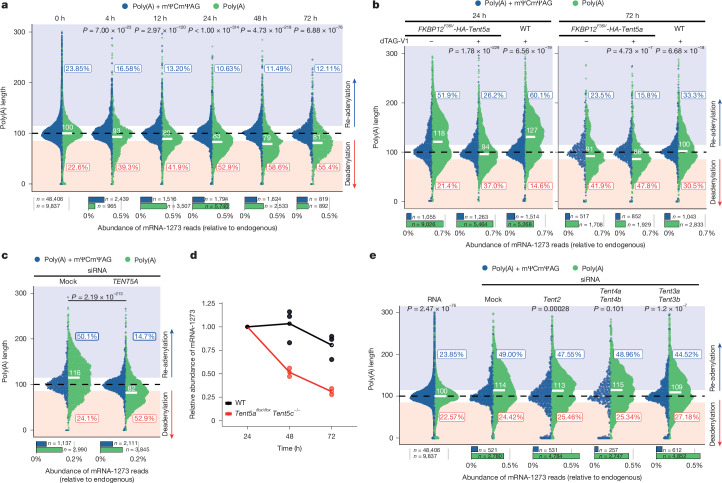
TENT5A specifically re-adenylates mRNA-1273 in macrophages, thus stabilizing it. **a**, mRNA-1273 poly(A) length distribution in *Tent5a*^*flox/flox*^*Tent5c*^−/−^ mBMDMs treated with mRNA-1273 for up to 72 h. **b**, mRNA-1273 poly(A) length distribution in mBMDMs after rapid depletion of TENT5A. mBMDMs derived from the *FKBP12*^*F36V*^*-HA-Tent5a* knock-in mouse line in the presence of the depletion inducer dTAG-V1 were treated with mRNA-1273 LNPs for 24 h (left) or 72 h (right). As controls, *FKBP12*^*F36V*^*-HA-Tent5a* cells without dTAG-V1, and wild-type cells with dTAG-V1, are shown. **c**, mRNA-1273 poly(A) length distribution in hMDMs transfected with either mock (left) or *TENT5A*-specific (right) siRNAs, treated with mRNA-1273 LNPs for 48 h. Average distributions from three different siRNAs in two biological replicates. **d**, Stability of mRNA-1273 in wild-type (black) or *Tent5a*^*flox/flox*^*Tent5c*^−/−^ (red) mBMDMs, calculated based on data from **a** and Fig. 2b. Abundance of mRNA-1273 normalized to 24 h time point. Mean values shown as empty circles. **e**, mRNA-1273 poly(A) length distribution in mBMDMs transfected with either mock or *Tent2*-, *Tent4a/Tent4b*- or *Tent3a/Tent3b*-specific siRNAs, treated with mRNA-1273 LNPs for 24 h. Average distributions from three different siRNAs in two independent biological replicates. For **a**–**c**,**e**, eDRS-measured poly(A) tail lengths are shown with reads containing (blue) or lacking (green) an mΨCmΨAG sequence, with median values and fractions of elongated or shortened tails shown. Bottom panels show the relative abundance of each group, with group sizes indicated. *P* values calculated with two-sided Wilcoxon test (on reads lacking mΨCmΨAG) with Benjamini–Hochberg correction. Data in **a**,**b** represent two independent experiments. See also Extended Data Fig. 5.

**Extended Data Fig. 5:**
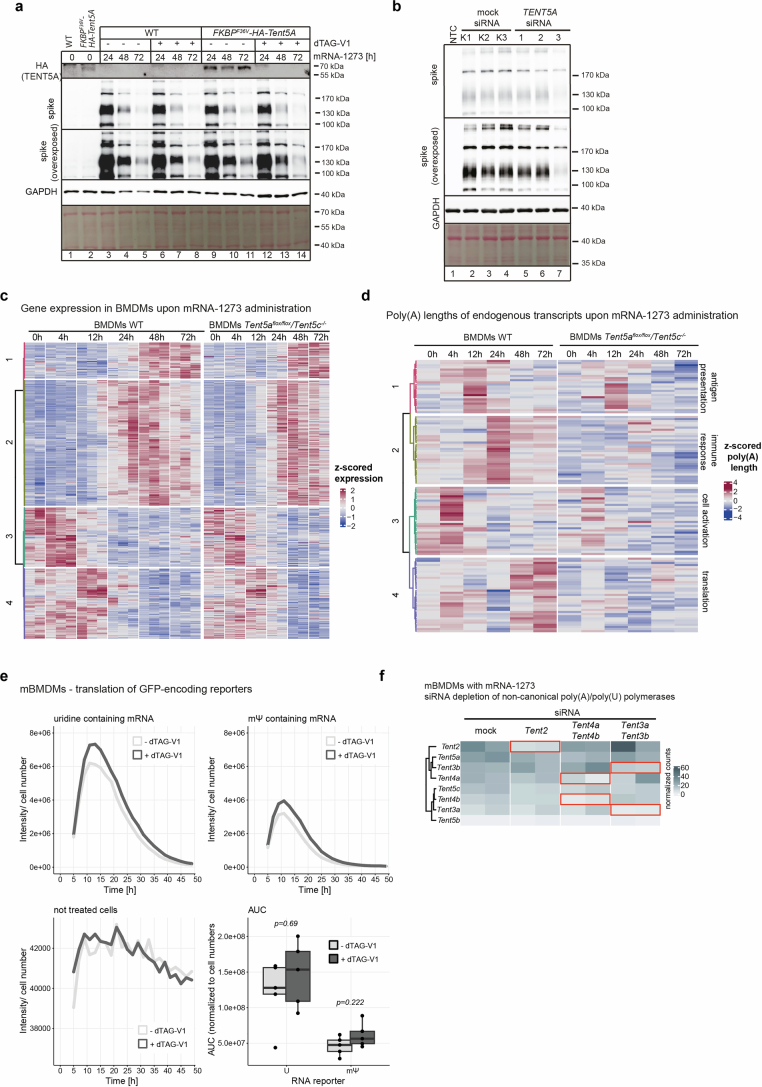
TENT5 poly(A) polymerases re-adenylate mRNA-1273. Related to Fig. 4. **a**, Expression of TENT5A (*FKBP12*^*F36V*^*-HA-Tent5a*) after mRNA-1273 administration (for up to 72 h) to mBMDMs derived from wild-type or *FKBP12*^*F36V*^*-HA-Tent5a* knock-in mouse line. Both lines were treated with dTAG-V1 inducer or untreated. Western blot on HA (to detect TENT5A) and spike; GAPDH and Ponceau (bottom) staining served as loading controls. N = 1. Please compare with Fig. 3e. **b**, Western blot for spike protein in hMDMs after *TENT5A* KD, 48 h after mRNA-1273 delivery. GAPDH and Ponceau (bottom) staining served as loading controls. Representative picture from 2 independent biological experiments. **c**, Expression of genes induced in mBMDMs upon mRNA-1273 addition, shown for wild-type (left) and (*Tent5a*^*flox/flox*^*/Tent5c*^*−/−*^) cells (right). Expression values were z-score normalized. Clusters and genes order as in Extended Data Fig. 4c. **d**, Changes in poly(A) tails lengths for mBMDMs wild-type (left) and (*Tent5a*^*flox/flox*^*/Tent5c*^*−/−*^) (right) in time course after addition of mRNA-1273. Z-score normalized poly(A) lengths are represented in colour scale. Clusters and gene order as in Fig. 3a. **e**, EGFP translation efficiency in mBMDMs derived from *FKBP12*^*F36V*^*-HA-Tent5a* knock-in mouse line. Cells were treated with dTAG-V1 inducer or untreated. EGFP intensity values are shown (normalized to cell number) for untreated (bottom left) or transfected with either uridine- (top left) or mΨ-mRNA (top right) cells in a time course after transfection. Average for 6 replicates. Area under curve was calculated for each condition (bottom right) and P values calculated using Wilcoxon test. Boxes show 25th-75th percentiles with median line; whiskers extend to 1.5×interquartile range (IQR). **f**, Expression (DESeq2-normalized counts) of non-canonical poly(A)/poly(U) polymerases in mBMDMs transfected with either mock or *Tent2*-, *Tent4a-* and *Tent4b*-, or *Tent3a-* and *Tent3b*-specific siRNAs, treated with mRNA-1273 for 24 h. Gene names are on the left. Individual replicates are shown. Red squares indicate genes silenced in a given condition.

To evaluate the role of TENT5 proteins in vaccine mRNA re-adenylation, we studied mRNA-1273 metabolism in mBMDMs lacking TENT5A and TENT5C (double-knockout *Tent5a*^*flox/flox*^*Tent5c*^−/−^). Cells were treated with mRNA-1273 for up to 72 h and RNA was isolated and subjected to eDRS. In comparison with wild-type mBMDMs, we observed relatively fast mRNA-1273 decay and deadenylation in the absence of TENT5A and TENT5C (Fig. 4a), with median poly(A) tails shortened to around 83 As after 24 h, despite the high ratio of RNAs lacking the 3′ pentamer. Rapid depletion of TENT5A protein in mBMDMs using the degron-inducible *FKBP12*^*F36V*^*-HA-Tent5a* model (Fig. 4b and Extended Data Fig. 5a) led to the same effect, with mRNA-1273 poly(A) tails shortened 24 h after vaccine administration, when compared with controls. The presence of the FKBP12(F36V) tag itself caused only a minor reduction in the activity of TENT5A, which was evident at both 24 h and 72 h after mRNA administration. Small interfering RNA (siRNA) knockdown of *TENT5A* in hMDMs also caused mRNA-1273 poly(A) shortening and decreased antigen production, when compared with mock-siRNA-transfected cells, and this effect was observed for three independent siRNAs (Fig. 4c and Extended Data Fig. 5b). All of these data indicate that TENT5 proteins have a crucial role in the regulation of mRNA-1273, with TENT5A being particularly key. In line with this, in *Tent5a*^*flox/flox*^*Tent5c*^−/^^−^ mBMDMs, the stability of mRNA-1273 is markedly reduced (Fig. 4d).

Notably, dysfunction of TENT5-mediated cytoplasmic polyadenylation had no effect on the transcriptional response, which was similar in both wild-type and *Tent5a*^*flox/flox*^*Tent5c*^−/−^ mBMDMs (Extended Data Fig. 5c and Supplementary Table 10). A negative effect of the knockout was observed only for poly(A) tails (Extended Data Fig. 5d), as in the case of LPS-activated macrophages^24^. Furthermore, TENT5A dysfunction does not affect the global translation efficiency, as revealed by high-content microscopy fluorescence quantifications of EGFP-encoding reporter mRNAs (Extended Data Fig. 5e).

Finally, to look at the potential effect of other non-canonical poly(A)/poly(U) polymerases on mRNA-1273 poly(A) tails, we depleted *Tent2*, *Tent4a*, *Tent4b*, *Tent3a* and *Tent3b* in mBMDMs and subsequently administered mRNA-1273. In all cases, we observed efficient re-adenylation of mRNA-1273 (Fig. 4e and Extended Data Fig. 5f), which was lost only in *Tent5a*^*flox/flox*^*Tent5c*^−/−^ cells (Fig. 4a). We conclude that TENT5A is responsible for mRNA-1273 re-adenylation in macrophages.

## Re-adenylation of ER-targeted IVT mRNAs

**Fig. 5:**
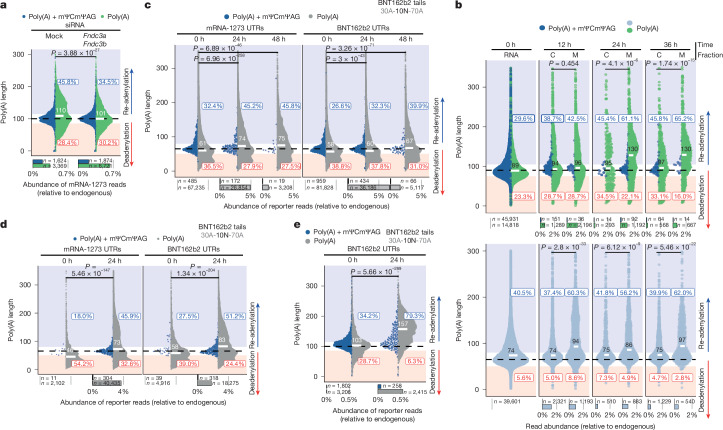
ER targeting specifies the TENT5-mediated re-adenylation of synthetic mRNAs. **a**, eDRS-measured mRNA-1273 poly(A) length distribution 24 h after administration to mock (left) or *Fndc3a-* and *Fndc3b-*depleted mBMDMs. Average distributions from three different siRNAs in two replicates. **b**, cDNA-seq-measured poly(A) length distribution for mRNA-1273 (top) and BNT162b2 (bottom) co-transfected to wild-type mBMDMs in equal amounts, showing cytoplasmic (C) and membrane (M) fractions over time (up to 36 h). **c**, cDNA-seq-measured OVA reporter poly(A) length distribution in mBMDMs transfected with mRNAs that have BNT162b2 or mRNA-1273 UTRs and BNT162b2-like poly(A) tails (with terminal mΨCmΨAG) for up to 48 h. The type of UTRs in the reporter is indicated at the top. **d**,**e**, PfCSP reporter poly(A) length distribution in mBMDMs transfected with mRNAs that have BNT162b2-like poly(A) tails with a terminal mΨCmΨAG for up to 24 h, analysed with either cDNA-seq (**d**) or eDRS (**e**). Type of UTRs in the reporter indicated at the top. For all panels, reads are divided into those with (blue) or without (green or grey) an mΨCmΨAG sequence, with median values and fractions of elongated or shortened tails shown. Average distributions for two biological experiments (**a**–**d**) or a single biological experiment (**e**). Bottom panels show the relative abundance of each group, with group sizes indicated. *P* values calculated with two-sided Wilcoxon test (on reads lacking mΨCmΨAG) with Benjamini–Hochberg correction. See also Extended Data Figs. 6–9.

**Extended Data Fig. 6:**
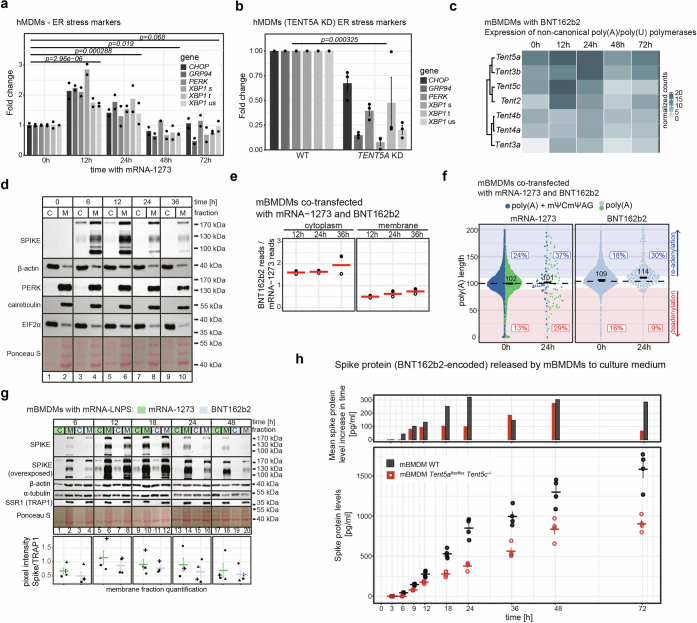
ER targeting specifies TENT5-mediated re-adenylation of mRNAs. Related to Fig. 5. **a**,**b**, ER stress marker expression in wild-type hMDMs after administration of mRNA-1273 for up to 72 h (**a**) and in hMDMs with siRNA-mediated *TENT5A* KD, 48 h after mRNA-1273 delivery (**b**). *XBP1* s—spliced, us—unspliced, t—total. Data from 2 (**a**) and 3 (**b**) independent biological experiments. *P* values calculated for fold changes using two-sided Wilcoxon test, Benjamini–Hochberg correction, time points (without dividing by transcripts) considered as individual groups. **c**, Expression (DESeq2-normalized counts) of non-canonical poly(A)/poly(U) polymerases in mBMDMs treated with BNT162b2 for up to 72 h. Gene names on the left. N = 2 independent biological experiments. **d**, Spike protein in cytoplasmic (C) or membrane (M) fractions of mBMDMs after cotransfection of mRNA-1273 and BNT162b2 (for up to 36 h). Western blot for spike; calreticulin, PERK and EIF2a served as a control of purity of fractions; β-actin and Ponceau S staining (bottom) served as loading controls. Representative picture from 2 independent biological experiments shown. **e**, Ratios of BNT162b2 to mRNA-1273 reads in cytoplasmic and membrane fractions of mBMDMs (data from Fig. 5b). N = 2 independent biological experiments. Median values shown in red. **f**, DRS measured poly(A) length distribution for mRNA-1273 (left) and BNT162b2 (right) co-transfected (with lipofectamine reagent) to wild-type mBMDMs. RNA was analysed 24 h post-transfection. For the left panel reads are divided into containing (blue) or lacking (green) mΨCmΨAG sequence, with median values and fractions of elongated or shortened tails shown. Bottom: relative abundance of each group, with group sizes indicated. *P* values calculated with two-sided Wilcoxon test (on reads lacking mΨCmΨAG) with Benjamini–Hochberg correction. Data represent 2 independent biological experiments, **g**, Spike protein in the cytoplasmic (C) or membrane (M) fractions of mBMDMs after mRNA-1273 (green) or BNT162b2 (blue) administration (for up to 36 h). Western blot for spike; controls for fraction purity include SSR1 (TRAP1) and loading controls such as β-actin and α-tubulin. Representative picture from 3 independent experiments. Bottom, quantification of spike protein in membrane fraction (3 biological and 1 technical replicate, normalized to TRAP1 levels), mean values ± s.e.m. indicated in colour. **f**, **h**, Amount of spike protein (assessed with ELISA) in the culture medium, up to 72 h after administration of BNT162b2 to either wild-type (black) or *Tent5a*^*flox/flox*^/*Tent5c*^*−/−*^ (red) mBMDMs. The average increase in spike protein amount per time point (top) or absolute measured concentrations (bottom) are shown. Mean values ± s.e.m. indicated. N = 4 independent biological experiments. Source data

As a next step, we focused on factors determining TENT5A substrate specificity. Most of the mRNAs with poly(A) tails that were extended after mRNA-1273 vaccination in wild-type but not in *Tent5a*^*flox/flox*^*Tent5c*^−/−^ mBMDMs (Extended Data Fig. 5d) encode proteins that are translated at the ER. Notably, mRNA-1273 also encodes an ER-targeted protein, which, as we show, hijacks TENT5 proteins to extend its poly(A) tails. Massive translation of spike (and other proteins) at the ER, after mRNA-1273 administration, led to increased ER stress in hMDMs (Extended Data Fig. 6a). However, siRNA depletion of *TENT5A* resulted in decreased spike protein translation (Extended Data Fig. 5b) and reduced the levels of unfolded protein response markers (Extended Data Fig. 6b), consistent with the role of TENT5A in promoting vaccine antigen translation. TENT5C has previously been shown to localize to the ER through interaction with ER-associated transmembrane FNDC3A and FNDC3B proteins^25^. Depleting *Fndc3a* and *Fndc3b* in mBMDMs led to the abrogation of mRNA-1273 re-adenylation (Fig. 5a), indicating that, in the case of TENT5A, its cooperation with FNDC3A and FNDC3B is essential for the re-adenylation and stabilization of mRNA-1273.

**Extended Data Fig. 7:**
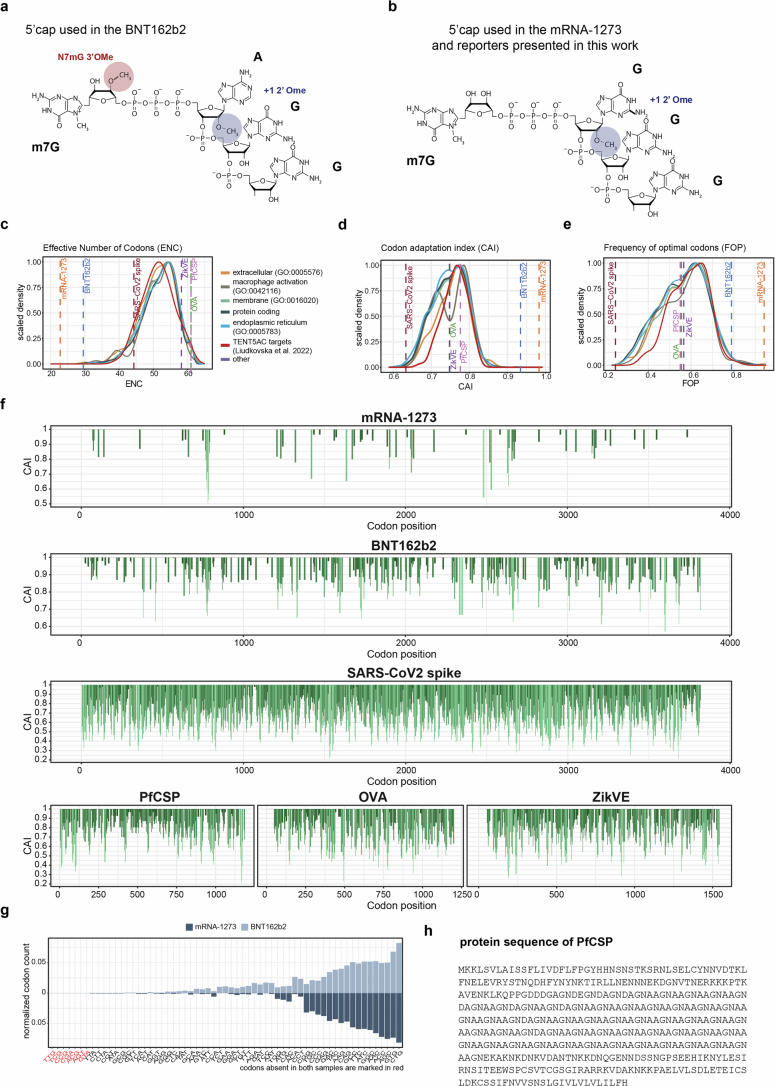
Sequence properties of mRNA-1273 and BNT162b2 vaccines and mRNA reporters. Related to Fig. 5. **a**,**b**, Structures of CleanCap AG 3′-OMe (**a**), used in BNT162b2 and Cap1 (**b**), used in the mRNA-1273 and enzymatically capped mRNA reporters analysed in this work. CleanCap used for the synthesis of reporters presented in Extended Data Figs. 8c and 9a–c is the same as in **b**, but with A nucleotide in the first position. **c**–**e**, Effective number of codons (ENC, **c**), codon adaptation index (CAI, **d**), and frequency of optimal codons (FOP, **e**) values for vaccine and reporter mRNAs CDSs used in this study, compared to average distributions for various groups of endogenous mouse transcripts. Shared legend for all the panels is presented on panel **c**. **f**, Sliding-window analysis of CAI of reporter mRNAs CDSs used in this study. **g**, Frequency of individual codons in mRNA-1273 and BNT162b2 CDSs. **h**, Protein sequence of PfCSP reporter.

Because our previous studies in different cell types revealed that the specificity of TENT5 proteins is not determined by sequence motifs^5–7,24^, and that the presence of an ER targeting signal is sufficient to drive TENT5-mediated regulation^6^, the efficient re-adenylation of mRNA-1273 but not BNT162b2 was puzzling. Given that coronavirus spike proteins are co-translationally translocated to the ER^26^, we sought to further investigate the spatial determinants of vaccine mRNA metabolism. Because in mBMDMs, *Tent5a* is more strongly induced by mRNA-1273 than by BNT162b2 (Fig. 3d and Extended Data Fig. 6c), we co-transfected mRNA-1273 and BNT162b2 to mBMDMs with lipofectamine-based transfection reagent, to exclude the differential effects of each vaccine on transcriptomic response. Next, we fractionated cells after vaccine administration in a time-course manner and performed nanopore cDNA sequencing of isolated RNA. This analysis revealed robust re-adenylation of not only mRNA-1273 but also BNT162b2, specifically in the membrane fraction (Fig. 5b and Extended Data Fig. 6d), confirming that ER association is a prerequisite for efficient re-adenylation. The lower load of BNT162b2 on ER (Extended Data Fig. 6e) and the inability to distinguish between intact and processed vaccines—unlike mRNA-1273, for which reads ending in mΨCmΨAG can be excluded—are likely to explain why re-adenylation of BNT162b2 is less pronounced in the total fraction (Extended Data Fig. 6f). Next, we administered both vaccines to mBMDMs separately and analysed the dynamics of antigen production. The spike protein was present in the membrane fraction, with a trend towards lower levels of BNT162b2-encoded than mRNA-1273-encoded spike protein on the ER, mainly at early time points (Extended Data Fig. 6g). This aligns with the results from our analysis of whole-cell lysates and culture medium, and with the in vitro translation assay presented before (Figs. 1d and 2e and Extended Data Fig. 3j,k). It could be caused by a combination of effects of differences in the cap structure (Extended Data Fig. 7a,b), the codon optimality of the coding sequence (CDS) (Supplementary Note 7, Extended Data Fig. 7c–g) and the UTRs. Nevertheless, despite relatively inefficient re-adenylation, *Tent5a*^*flox/flox*^*Tent5c*^*−/*^^*−*^ mBMDMs produce less spike protein, compared with wild-type mBMDMs, after BNT162b2 administration over a time course (Extended Data Fig. 6h), indicating that TENT5A also has a beneficial effect on antigen production for this vaccine.

**Extended Data Fig. 8:**
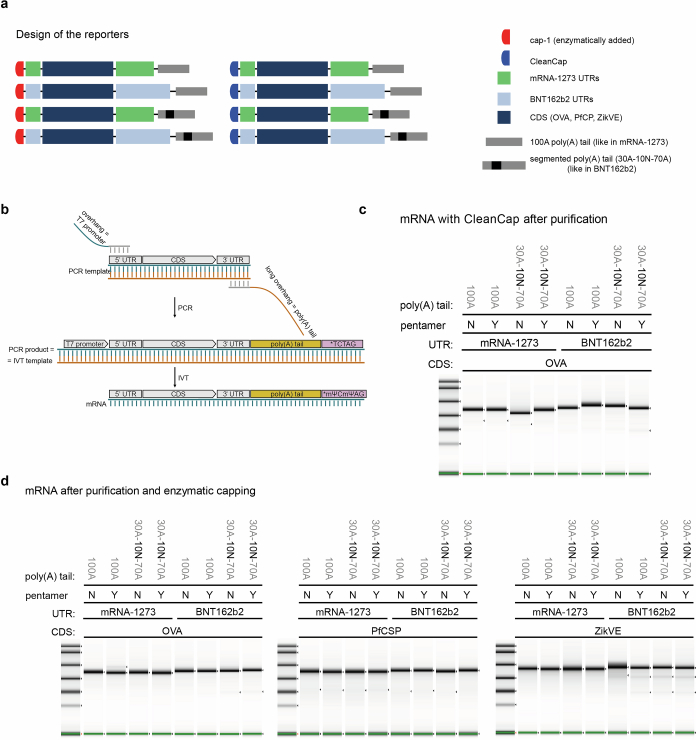
Generation of ER-targeted mRNA reporters. Related to Fig. 5. **a**, Schematic representation of the design of IVT mRNA reporters used in this work. **b**, Illustration of IVT mRNA reporters synthesis. Templates for IVT reaction are prepared with the PCR, adding T7 promoter on one of the primers and desired poly(A) tail on the second primer. **c**,**d**, Integrity and purity of synthesized mRNAs, assessed with TapeStation 2200. Separate pictures are shown for mRNAs with cotranscriptionally added CleanCap (**c**) or with post-transcriptionally enzymatically (VCE + MTA) added cap (**d**).

**Extended Data Fig. 9:**
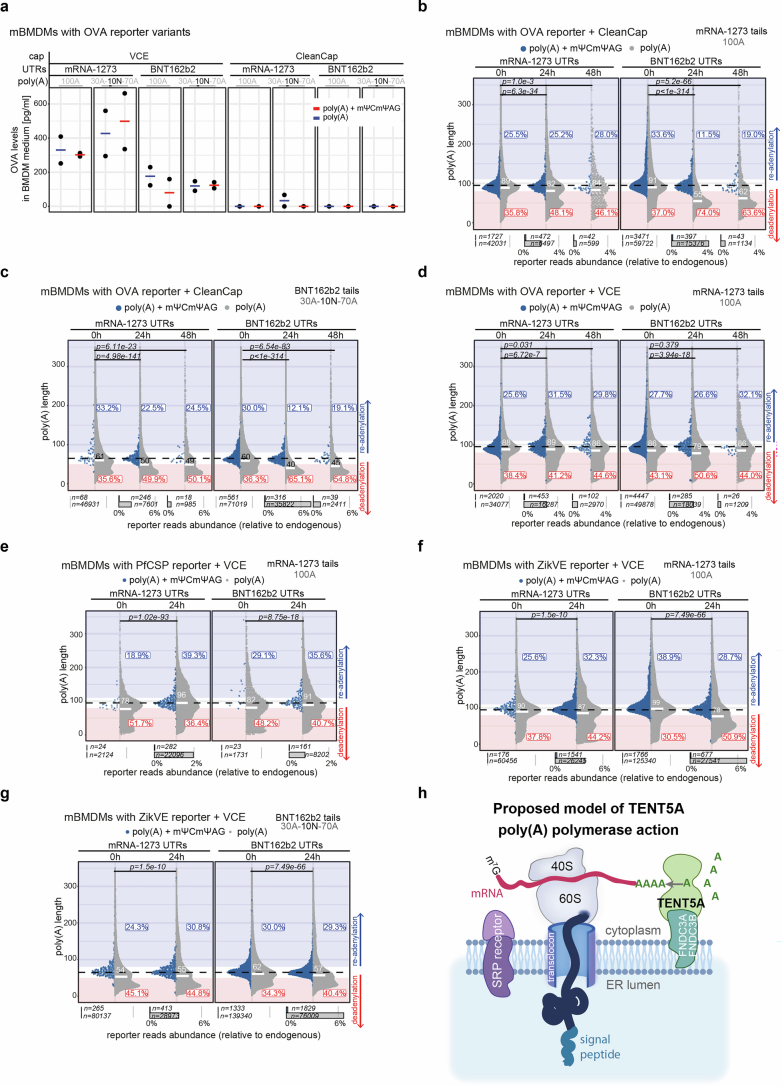
TENT5-mediated re-adenylation of synthetic mRNAs. Related to Fig. 5. **a**, Amount of OVA (assessed with ELISA) in the culture medium, 24 h after administration of OVA reporter mRNA to mBMDMs. Type of capping, UTRs, poly(A) tails and presence (or lack) of terminal pentamer are indicated. 2 independent biological replicates shown. **b**,**c**, CleanCap-equipped OVA reporter poly(A) length distribution in mBMDMs transfected with mRNAs possessing mRNA-1273- (**b**) or BNT16b2-like (**c**) poly(A) tail (both with terminal mΨCmΨAG) for up to 48 h. The type of UTRs in the reporter is indicated at the top. **d**, VCE-capped OVA reporter poly(A) length distribution in mBMDMs transfected with mRNAs possessing mRNA-1273-like poly(A) tail (with terminal mΨCmΨAG) for up to 48 h. The type of UTRs in the reporter is indicated at the top. **e**, VCE-capped PfCSP reporter poly(A) length distribution in mBMDMs transfected with mRNAs possessing mRNA-1273-like poly(A) tail (with terminal mΨCmΨAG) for up to 24 h. The type of UTRs in the reporter is indicated at the top. **f**,**g**, VCE-capped ZikVE reporters poly(A) length distribution in mBMDMs transfected with mRNAs possessing mRNA-1273- (**f**) or BNT16b2-like (**g**) poly(A) tail (both with terminal mΨCmΨAG) for up to 24 h. The type of UTRs and poly(A) tails in the reporter are indicated at the top. **h**, Model of of TENT5A poly(A) polymerase’s action, indicating ER-localization as a prerequisite for efficient mRNA adenylation by TENT5A. For **b**–**g**, poly(A) tail lengths measured with nanopore cDNA sequencing are shown with reads containing (blue) or lacking (grey) mΨCmΨAG sequence, with median values and fractions of elongated or shortened tails shown. Bottom panels: relative abundance of each group, with group sizes indicated. *P* values calculated with two-sided Wilcoxon test (on reads lacking mΨCmΨAG) with Benjamini–Hochberg correction. Data represent 2 independent experiments.

Next, to systematically evaluate the influence of the capping strategy, UTRs and poly(A) tail composition (including the presence of mΨCmΨAG) on mRNA re-adenylation in mBMDMs, we generated a series of mΨ-containing reporter mRNAs (Supplementary Note 6 and Extended Data Fig. 8a,b). First, 16 variants of ER-targeted codon-optimized ovalbumin (OVA)-coding mRNAs (Extended Data Fig. 8c,d) were used to evaluate protein production using an enzyme-linked immunosorbent assay (ELISA). Notably, we observed that mBMDMs transfected with the enzymatically VCE + MTA-capped mRNA (Cap 1) produced several folds more OVA than did mBMDMs that were transfected with mRNA cotranscriptionally equipped with CleanCap (Extended Data Fig. 9a). Besides the capping method, mRNA-1273 UTRs had a beneficial effect on OVA production, compared with those derived from BNT162b2 (Extended Data Fig. 9a). Poly(A) tail and mΨCmΨAG sequence seemed to have a minor role despite being synthesized from different DNA templates.

For the analysis of poly(A) lengths, we focused on mRNAs ending with mΨCmΨAG, which, as shown before, does not affect expression and is usually removed before re-adenylation (Extended Data Fig. 3h), thus allowing us to analyse the processed mRNAs without fractionation, unlike the original BNT162b2. Consistent with the correlation of protein expression and translation efficiency, re-adenylation was completely absent in OVA mRNAs equipped with CleanCap, as revealed by nanopore cDNA sequencing (Extended Data Fig. 9b,c). At the same time, a certain degree of re-adenylation was observed in all variants of enzymatically capped RNAs (Fig. 5c and Extended Data Fig. 9d), despite CDS optimization being significantly different from that of SARS-CoV-2 vaccines (Extended Data Fig. 7c–f and Supplementary Note 7). Re-adenylation was slightly more pronounced in reporters with mRNA-1273 UTRs and BNT162b2 poly(A) tails.

Finally, to check the re-adenylation potency of other CDSs, we designed reporters encoding an ER-targeted circumsporozoite protein from *Plasmodium falciparum* (PfCSP; Extended Data Fig. 7h) and the Zika virus envelope structural protein (ZikVE), considered antigens in the mRNA vaccines against malaria^9^ and Zika virus^8^, respectively. Again, cDNA sequencing of RNA isolated from mBMDMs 24 h after transfection revealed that all enzymatically capped variants were re-adenylated, with no clear trend with regard to the superiority of particular UTRs or poly(A) tail sequences (Fig. 5d and Extended Data Fig. 9e–g). Notably, reanalysis of the PfCSP reporter with BNT162b2 UTRs and BNT162b2 tail using eDRS, which is more accurate than cDNA sequencing on long poly(A) tails, revealed massive re-adenylation in mBMDMs (Fig. 5e).

Overall, we show that TENT5A-mediated re-adenylation stabilizes a broad range of enzymatically capped ER-targeted IVT mRNAs, including therapeutic ones (Extended Data Fig. 9h). This further indicates that the potency of re-adenylation is affected by several factors, such as the capping method and the coding and UTR sequences, whereas poly(A) tail composition has a relatively minor role.

## TENT5A enhances the efficacy of mRNA vaccines

**Extended Data Fig. 10:**
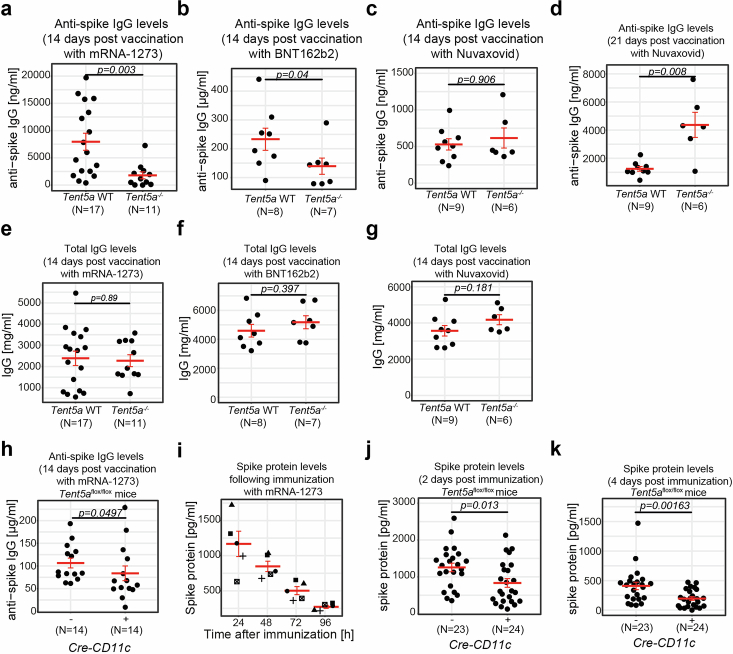
mRNA-1273 polyadenylation by TENT5s is essential for the efficient production of antigen and immune response. **a**,**b**, Anti-spike IgG levels in serum of wild-type (WT) and *Tent5a*^*−/−*^ mice 14 days after immunization with mRNA-1273 (WT n = 17, KO n = 11) (**a**) or BNT162b2 (WT n = 8, KO n = 7) (**b**). **c**,**d**, Anti-spike IgG levels in serum of wild-type and *Tent5a*^−/−^ mice 14 (**c**) or 21 (**d**) days after immunization with Nuvaxovid (WT n = 9, KO n = 6), **e**–**g**, Total IgG levels in serum of mRNA-1273 (**e**), BNT162b2 (**f**), or Nuvaxovid (**g**) immunized mice, wild-type and *Tent5a*^−/−^, measured 14 days after immunization. **h**, Anti-spike IgG levels in CD11c-CRE(−); *Tent5a* and CD11c-CRE(+);*Tent5a*^*flox/flox*^ mice 14 days after immunization with mRNA-1273 (CD11c-CRE(−) n = 14, CD11c-CRE(+) n = 14). **i**, Spike protein levels in the serum of wild-type immunized mice, 24 h, 48 h, 72 h and 96 h after immunization with mRNA-1273. *n* = 5. **j**,**k**, Spike protein levels in serum of immunized mice, CD11c-CRE(−); *Tent5a* and CD11c-CRE(+);*Tent5a*^*flox/flox*^, measured 48 h (**j**) or 96 h (**k**) after immunization. For all panels values were obtained with the ELISA assay. Mean values ± s.e.m. indicated in red. *P* values were calculated using two-sided Wilcoxon test. Source data

DCs are major antigen-presenting cells and are expected to have a key role in the development of immunity after vaccination. However, DCs express low levels of TENT5A (Fig. 3f,g), raising the question of whether TENT5-mediated stabilization of vaccine mRNA in macrophages affects immune response. *Tent5a*-knockout mice, despite having substantially lower body mass and serious skeletal malformations^5^, do not exhibit major immune defects, which might have an effect on immune response, as revealed by data from the International Mouse Phenotypic Consortium^27^ (www.mousephenotype.org). Specifically, they show no phenotypes related to T cells, B cells or DCs, only slightly elevated monocyte, neutrophil and granulocyte counts in females. Thus, we immunized wild-type and *Tent5a*^−/−^ mice with mRNA-1273, BNT162b2 or a Nuvaxovid (Novavax) protein-based COVID-19 vaccine, and measured antibody production (14 days after vaccination). The level of serum anti-spike IgG 14 days after immunization with mRNA-1273 was significantly lower in *Tent5a*^−/−^ than in wild-type mice (Extended Data Fig. 10a), with no significant change in the total level of IgGs in the sera (Extended Data Fig. 10e). Despite the differences in re-adenylation efficiency in the in vitro system, slightly lower serum anti-spike IgG levels were observed in *Tent5a*^−/−^ mice after BNT162b2 vaccination (Extended Data Fig. 10b,f). Of note, no negative effect of *Tent5a*^−/−^ on serum anti-spike IgG levels was observed for protein-based Nuvaxovid vaccine (Extended Data Fig. 10c,d,g). To more specifically address the role of vaccine uptake by macrophages in muscle, we immunized conditional knockout mice with Cre-induced ablation of *Tent5a* in CD11c^+^ cells. Similar to what was seen in *Tent5a*^−/−^ mice, the level of anti-spike IgG was lower at 14 days after immunization in *Tent5a*^*flox/flox*^ mice than in controls (Extended Data Fig. 10h). Next, we measured the levels of spike protein in the serum at 48 h and 96 h after immunization (Extended Data Fig. 10i), and found that the levels were significantly lower in *Tent5a*^*flox/flox*^ mice than in control mice (Extended Data Fig. 10j,k). Such an effect confirms the major role of CDC11c^+^ cells expressing *Tent5a* in antigen production. Thus, TENT5A-mediated re-adenylation, which occurs mainly in macrophages, has a direct effect on antigen production and vaccine efficacy.

## Discussion

The COVID-19 pandemic accelerated the development of vaccine technology, and the timely introduction of mRNA vaccines was key to slowing the spread of the causative virus, SARS-CoV-2. This report provides evidence that therapeutic mRNAs can be modified in cells, such that their poly(A) tails are extended by the cellular machinery. Re-adenylation leads to increased stability of targeted mRNAs and increased production of coded antigens, providing a possible explanation for the efficacy of existing mRNA vaccines (mRNA-1273 and BNT162b2 and their further generations). Our findings could have profound implications for the development of next-generation mRNA therapeutics, including some that are already in preclinical studies (for instance, mRNA-1325 and mRNA-1895 for Zika, encoding the premembrane and envelope proteins^8^). Moreover, our work highlights and reinforces the physiological role of the ER in cellular RNA metabolism, showing that the ER acts as a central hub not only for cellular mRNA translation but also for its post-transcriptional regulation. Mechanistically, the cytoplasmic poly(A) polymerase TENT5A, acting in concert with ER-associated FNDC3A and FNDC3B, is responsible for the re-adenylation of ER-targeted mRNA therapeutics. The exact mechanism of substrate selection by TENT5A has yet to be established, but the re-adenylation efficiency is affected by several factors. Notably, the capping strategy considerably influences re-adenylation. In primary macrophages, among OVA-coding mRNAs, only those with enzymatically incorporated Cap 1 underwent efficient poly(A) tail extension, whereas those cotranscriptionally capped with CleanCap did not. This correlated with significantly higher production of the encoded protein. Although the precise mechanism has yet to be elucidated, enzymatically capped mRNA-1273 was expressed at a higher level and underwent more robust adenylation than BNT162b2, which used CleanCap. However, the observed effect was less pronounced than it was for OVA mRNAs, suggesting that factors beyond the chemical cap structure are involved. Furthermore, UTR sequences can affect re-adenylation efficiency, although they do not have a dominant role. This observation aligns with the fact that TENT5A substrates lack distinct sequence motifs identifiable within the non-protein-coding regions of mRNAs^5–7^. Further research is needed to uncover all the details of TENT5-mediated re-adenylation.

Once administered, mRNA vaccines generate complex immune responses. Before COVID-19, research on mRNA vaccines focused mainly on the development of anti-cancer agents^21,28^. Although mRNA design has typically been optimized in cell lines, the aim has been to target DCs, which were thought to produce and present the antigen to generate both humoral and cellular adaptive immune responses. Here we show that macrophages are responsible for antigen production and thus have a crucial role in the development of immunity. Macrophages are not only the major cell population that takes up the vaccine mRNA^15,19^, but they also express TENT5A, which is induced after immunization and re-adenylates them, increasing stability and protein output. Accordingly, in mice that lack TENT5A in CD11C^+^ cells, a substantial reduction in antigen production is observed, associated with the impaired development of an antigen-specific humoral response.

The development of therapeutic mRNAs extends beyond vaccines, and intensive efforts are being made to use mRNA to treat Mendelian diseases or to induce the production of therapeutic proteins. In these cases, the main target is the liver, because transfecting hepatocytes after intravenous administration of LNP-encapsulated mRNAs is very efficient. There are also attempts to target other organs and cell types. Here we show that different cell types metabolize mRNA vaccines differently. We expect that only cells that express high levels of TENT5s stabilize the mRNA by re-adenylation and are thus desirable targets for therapeutic mRNAs that encode secreted proteins. In addition, a better understanding of substrate recognition will enable the rational design of mRNAs that are more efficiently polyadenylated by TENT5 and are, therefore, more stable.

In the future, other tissue-specific mechanisms of mRNA stabilization might be uncovered. All of these observations emphasize the point that the design of therapeutic mRNAs must be tailored to the target cells and the target site of the protein product.

## Methods

### Mouse lines

All mouse lines were generated by CRISPR–Cas9 at the Genome Engineering Unit (https://crisprmice.eu/) as described previously^3,5,7^. A conditional *Tent5a*^*flox/flox*^ and double-knockout *Tent5a*^*flox/flox*^*Tent5c*^−/−^ have been described previously^7^. The Cd11c-Cre (B6N.Cg-Tg(Itgax-cre)1-1Reiz/J) mouse strain was purchased from The Jackson Laboratory and crossed with *Tent5a*^*flox/flox*^. The *FKBP12*^*F36V*^*-HA-Tent5a* mouse line was generated by inserting the sequence of the dTag degron and the 2×HA tag at the N terminus of the endogenous *Tent5a*. B6CBAF1 zygotes were microinjected with a mixture containing *Cas9* mRNA, gRNA targeting *Tent5a* (AAAAGTATCTCTGATGCATC) and double-stranded repair template (TAGAAGGGGCGGCCGCCTCCAGGTGACACAGACGGGACTCTCGCTTGTGCTTTCCAGATGGGAGTGCAGGTGGAAACCATCTCCCCAGGAGACGGGCGCACCTTCCCCAAGCGCGGCCAGACCTGCGTGGTGCACTACACCGGGATGCTTGAAGATGGAAAGAAAGTTGATTCCTCCCGGGACAGAAACAAGCCCTTTAAGTTTATGCTAGGCAAGCAGGAGGTGATCCGAGGCTGGGAAGAAGGGGTTGCCCAGATGAGTGTGGGTCAGAGAGCCAAACTGACTATATCTCCAGATTATGCCTATGGTGCCACTGGGCACCCAGGCATCATCCCACCACATGCCACTCTCGTCTTCGATGTGGAGCTTCTAAAACTGGAAGGCGGCTACCCCTACGACGTGCCCGACTACGCCGGCTATCCGTATGATGTCCCGGACTATGCAGGCGGACATCAGAGATACTTTTGGTGCGGGGCTGCTCTGCGGGGCGCGGCGCGCGGCTGGGCATTG). Zygotes were transferred to surrogate mothers and pups were screened for the presence of insertion using a 3-primer approach (Fw: GCAGCTCCTGCGAAGTGTG, Rv1: TGCCATGGCAAAGTACCCTTC, Rv2: CCCACACTCATCTGGGCAAC). Potential founders were sequenced by Sanger sequencing. Mice were bred in the animal facility of the Faculty of Biology, University of Warsaw, and maintained under conventional conditions^3,5,7^ in open polypropylene cages filled with woodchip bedding enriched with nesting material and paper tubes. Mice were fed ad libitum with a standard laboratory diet (Labofeed B, Morawski). Room humidity was maintained at 55 ± 10%, the temperature was kept at 22 °C  ± 2 °C, the air was changed at least 15 times per hour and a 12-h–12-h light regime was used (lights on from 06:00 to 18:00). Regular health monitoring was performed at the IDEXX laboratory. Mice of different genotypes were assigned with individual numerical tags in the database and they were used for tissue collection and throughout the subsequent processing as the only identifiers.

Discarded residual vaccine material: mRNA-1273 (Moderna, monovalent or bivalent original/Omicron BA.4-5 spike), BNT162b2 (Pfizer monovalent or bivalent original/Omicron BA.4-5), Nuvaxovid (Novavax, recombinant spike protein, adjuvanted) were used within the manufacturer’s stability guidelines. Because this was not available for purchase at the time and because only residual (otherwise discarded) material could be used, we obtained approval from the Polish Ministry of Health (MMI.454.1.2021.TM).

All animal experiments were approved by the II Local Ethical Committee in Warsaw (approval numbers: WAW2/71/2021, WAW2/129/2021, WAW2/95/2022, WAW2/127/2022 and WAW2/007/2023) and were performed according to Polish law (act number 653 266/15.01.2015) and in agreement with the corresponding European Union directive.

### Immunizations

Six-to-fourteen-week-old mice were immunized by intramuscular (*Vastus lateralis* region) injection with 0.9% NaCl (controls), 1 µg mRNA-1273 (Moderna) or BNT162b2 mRNA (Pfizer) or 50 µl Nuvaxovid vaccine (Novavax) at 40 ng µl^−1^ protein concentration. Tissues were collected at set time points for subsequent analyses. Mice of both sexes were used, with equal or similar ratios of both sexes in directly compared groups. The sex of the mice was not considered in the immunization experiments, only the genotype.

No formal sample-size calculations were performed a priori. Sample sizes were determined on the basis of established practices in similar published studies and the 3Rs principles (replacement, reduction and refinement) for ethical animal research. We used the minimum numbers necessary to achieve statistical significance while maintaining scientific rigour, on the basis of our previous experience and comparable studies. Details on sample sizes are provided for each experiment. Mice with different genotypes could not be distinguished by the experimenter (except for *Tent5a*^−/−^ mice, which have a distinguishable phenotype). However, vaccination is a routine procedure and therefore knowledge about the genotype of mice at the time of vaccination was highly unlikely to affect the results of immunization.

### ELISA

Concentrations of spike protein receptor-binding domain, anti-spike IgG and total IgG in mice serum were measured according to the manufacturer’s recommendations with commercially available ELISA kits: Invitrogen (EH492RB; 1222062723, 1222110123 and 1222110723), Krishgen Biosystems (KBVH015-14; MACOVSPQ1023, MACOVSPQ0223 and MCOV2SS0822) and Eagle Biosciences (OGG11-K01; 25Q1 and 26), respectively. The level of OVA was measured using an ELISA kit from MyBioSource (MBS2000240; L240916286). Sample dilution was determined experimentally to fit within the standard curve of each ELISA. Owing to intervals between immunizations, different versions and lots of both vaccines and ELISA kits were used, depending on availability. Therefore, ELISA results should be interpreted as semi-quantitative between experiments. A single experiment was always performed simultaneously for all conditions studied, and can therefore be interpreted as quantitative, allowing direct comparisons between experimental conditions.

### Sorting infiltrating macrophages and DCs from muscles

#### Muscle dissociation and isolation of immune cells

Muscles from anterior thigh were isolated 6 h, 12 h, 24 h or 48 h after vaccine injection, finely minced using surgical scissors and resuspended in 2 ml of serum-free RPMI supplemented with 1,000 U ml^−1^ collagenase from *Clostridium histolyticum* (Sigma-Aldrich, C9407), 250 µg ml^−1^ DNAse I (Sigma-Aldrich, 10104159001) and 8 µg ml^−1^ dispase I (Sigma-Aldrich, D4693). Samples were incubated for 30 min at 37 °C with 140-rpm agitation. Digested muscle suspensions were put on ice and processed at 4 °C from then on. Ten millilitres of ice-cold FACS buffer (0.2% BSA in PBS) was added to muscle suspensions, followed by straining with a 70-µm nylon strainer (Greiner, 542070). With the strainer on, the suspension was centrifuged at 300*g* for 7 min. The crude pellet was treated twice with ACK (150 mM ammonium chloride, 1 mM potassium bicarbonate and 0.1 mM disodium EDTA, pH 7.2) for 2 min on ice, followed by washing with 10 ml FACS buffer; each time, centrifugation was performed at 300*g* for 7 min. The crude pellet was then resuspended in 1 ml FACS buffer and strained through the 30-μm cap strainer of a FACS tube. With caps on, the samples were centrifuged at 800*g* for 3 min at 4 °C and the supernatant was discarded, followed by resuspension in 2 ml FACS buffer. Equal volumes of samples were mixed thoroughly with 33% Percoll (GE17-0891-01) in PBS and spun at 800*g* for 30 min at 4 °C. The supernatant was discarded and the cell pellet was subjected to staining.

#### Cell staining and sorting

Cells were washed once with FACS buffer and incubated with Fc block anti-CD16/32 (clone 2.4G2) (BD Biosciences, 553142) for 5 min on ice, washed with FACS buffer and incubated with anti-CD45–PerCP–Cy5.5, anti-CD11b–BV786, anti-F4/80–PE–Cy7, anti-I-A/I-E–BV605, anti-CD11c–PE and anti-CD64–APC–Fire750 for 40 min in 4 °C, protected from light (detailed information on antibodies and dilutions is provided in Supplementary Table 11). After staining, cells were washed with FACS buffer and then stained with the LIVE/DEAD Fixable Violet Dead Cell Stain Kit (Thermo Fisher Scientific, L34964) for 15 min at 4 °C, protected from light. Cell sorting was performed in a CytoFlex SRT cell sorter (Beckman Coulter) operated by CytExpert SRT v.1.1 software. A restrictive gating strategy was used to sort M0 macrophages and DCs (Supplementary Fig. 5). The gating strategy was based on gating live singlets, next cells identified as CD45^+^, CD11c^+^ and I-A/I-E (MHCII)^+^, with the exclusion of cells that were double positive for CD64 and F4/80, were sorted as DCs. Live singlets identified as CD45^+^, CD11c^+^, I-A/I-E (MHCII)^low/−^, F4/80^+^ and CD64^+^ were sorted as M0 macrophages. For post-sort analysis, cells were sorted to 100 µl of PBS. Post-sort analysis was performed on the first sample, to verify the purity of sorted populations. For RNA isolation, cells were sorted directly into 100 μl or 250 µl of extraction buffer (Applied Biosystems, KIT0204), depending on the cell amount, followed by centrifugation at 14,000*g* for 5 min and freezing until further processing.

### Cell cultures

A549 and HEK293 Flp-In T-REx cell lines and their derivatives were cultured in Dulbecco’s modified Eagle’s medium (DMEM; Gibco) supplemented with 10% FBS (Gibco) and penicillin–streptomycin (Sigma-Aldrich) at 37 °C in a 5% CO_2_ atmosphere until 80% confluency. To produce the HEK293 Flp-In T-REx (R78007, Thermo Fisher Scientific) cell line with conditional knockdown of *CNOT1*, we used a strategy previously described by us^29^. A tri-miRNA construct (listed below; stem–loop sequences ATGGAAGAGCTTGGATTTGAT, CTCCCTCAATTCGCCAACTTA and AGGACTTGAAGGCCTTGTCAA) was designed, and cloned at the BspTI (AflII) + NotI restriction sites in a pKK-RNAi vector (pKK-BI16 nucCherry EGFP-TEV). A total of 1 × 10^6^ HEK293 Flp-In T-REx cells, grown in DMEM high-glucose medium (Gibco) supplemented with 10% FBS on six-well plates, were transfected with 300 ng of the *CNOT1*-bearing construct mixed with 1 μg of pOG44 plasmid and 250 μl of Opti-MEM medium, supplemented with 2 µl TransIT-2020 transfection reagent (Mirus, MIR5400). The cells were seeded in a 60-mm plate and cultured in DMEM high-glucose medium supplemented with 40 µg ml^−1^ hygromycin and 8 µg ml^−1^ blasticidin for the first six to seven days. Cells were grown until single colonies appeared four weeks later. A control of cells that were not transfected with the pKK-BI16 plasmid was included to ensure the specific selection of the cell line. Expression of exogenous miRNA genes was induced by the addition of doxycycline (Thermo Fisher Scientific) at a final concentration of 100 ng ml^−1^. Cell enumeration was performed by crystal violet (Sigma, C3886) staining described by the laboratory of X. Chen (UCSF). In brief, cell medium was aspirated (or collected for subsequent ELISAs if necessary) and cells were washed with 1 ml PBS. Staining solution (0.25% crystal violet in 20% methanol) was then added to each well and cells were incubated for 10 min at room temperature. The staining solution was then removed, and the cells were rinsed 6 times with 2 ml PBS, after which the remaining solution was evaporated, and the plates were scanned.

### mBMDM cultures

The primary mBMDM cultures were established from the bone-marrow monocytes isolated from *Tent5a*^*flox/flox*^*Tent5c*^−/−^, *FKBP12*^*F36V*^*-HA-Tent5a* and wild-type mice. Young adult mice (12–25 weeks) of both sexes were euthanized by cervical dislocation. The femurs and tibias were isolated and bone marrow was collected using a centrifugation-based protocol^24,30^. Material was mixed from several individual mice (siblings of the same sex) to obtain sufficient cells for subsequent analyses. The sex of the mice used as a source of bone marrow cells was not considered in further analyses. Bone-marrow cells were plated in IMDM medium (Thermo Fisher Scientific, 21980065) supplemented with 10% FBS (Gibco), 100 U ml^−1^ penicillin and 0.1 mg ml^−1^ streptomycin solution (Sigma-Aldrich) and 10 ng ml^−1^ macrophage colony-stimulating factor (M-CSF; PeproTech, 315-02), and cultured at 37 °C in 5% CO_2_. For conditional *Tent5a* gene targeting, mBMDMs were transduced on the eighth day after isolation with lentivirus carrying Cre recombinase (pCAG-Cre-IRES2-GFP). Lentivirus production, cell transduction and genotyping were performed as described previously^3,7^. Cells were used for experiments on the fourteenth day after isolation.

To induce depletion of TENT5A, 0.7 × 10^6^ mBMDMs obtained from wild-type and *FKBP12*^*F36V*^*-HA-Tent5a* mice were seeded in six-well plates and 24 h later, degron was induced by the addition of 1 µM dTAGv-1 or dimethyl sulfoxide (DMSO) as a control^31^. The vaccine was added to cells at the same time and cells were collected in time courses at set time points.

### Analysis of translation dynamics in mBMDMs

mBMDMs were reversely transfected with IVT mRNA (with uridines or mΨ) encoding high-RSCU EGFP (ref. ^32^) using MessengerMAX (Invitrogen, LMRNA001) according to the manufacturer’s instructions in five technical replicates. In brief, the mRNA used for transfection was diluted in Opti-MEM (Gibco, 31985047) to obtain 40 ng of mRNA in 10 µl of Opti-MEM, and MessengerMAX (Invitrogen, LMRNA001) was diluted in Opti-MEM equivalent to 0.1 µl of reagent in 10 µl of Opti-MEM per well. Diluted mRNA was mixed with diluted transfection reagent on 96-well plates, left for 40 min and then transferred to 384-well plates (Perkin Elmer, 6057300). mBMDMs were counted in a Countess 3L counter (Invitrogen, AMQAF2000) using dedicated chamber slides (Invitrogen, C10283). Finally, cells were diluted to 5,000 cells per well and supplemented with dTAG-V1 (Bio-Techne, 7374/5) to a final concentration of 2 µM. Control cells were seeded without degron inductor. Cells were then seeded at a volume of 100 µl onto prepared plates with transfection mixes using the Multipette E3x (Eppendorf, 4987000029) and Combitip 5 ml Biopur (Eppendorf, 0030089669). The imaging was started 4 h after transfection in Opera Phenix from Perkin Elmer using three channels: 488 nm (time: 50 ms, power: 20%, height: −8.0 µm); bright-field (time: 20 ms, power: 20%, height: −0.0 µm); digital phase contrast (time: 20 ms, power: 20 %, height: −1.0 µm); water objective 20× non-confocal; binning 2 × 2. Live images were taken every 2 h for 72 h.

### Isolation of human CD14^+^ cells

Buffy coats were obtained commercially from the Regional Blood Centre in Warsaw. All donors were 18–45-year-old healthy men. Peripheral blood mononuclear cells (PBMCs) were isolated within 1–2 h after donation by density-gradient centrifugation using Lymphoprep (STEMCELL Technologies). Next, CD14^+^ PBMCs (monocytes) were isolated immunomagnetically with anti-human CD14 antibody-coated microbeads using LS columns (all from Miltenyi Biotec), strictly following the manufacturer’s protocol. CD14+ cells were counted, and viability was checked in 0.1% trypan blue solution (Sigma-Aldrich) and used for subsequent hMDM differentiation. Each isolation resulted in a viability of over 95%.

### In vitro culture of hMDMs

CD14^+^ PBMCs were seeded in a 10-cm tissue-culture-treated Petri dish at a density of 1 × 10^6^–2 × 10^6^ cells per ml in 10 ml of RPMI-1640 medium (Sigma-Aldrich) supplemented with 10% heat-inactivated FBS (HyClone), 2 mM l-glutamine (Sigma-Aldrich) 100 U ml^−1^ penicillin and 100 μg ml^−1^ streptomycin (both Sigma-Aldrich), 1% (v/v) MEM non-essential amino acids solution (Thermo Fisher Scientific) and 1 mM sodium pyruvate (Sigma-Aldrich) supplemented with 50 ng ml^−1^ of recombinant human M-CSF (for macrophage differentiation). Adherent cells collected from the M-CSF-supplemented culture were checked for markers of macrophages. hMDMs were stained with Zombie UV or Zombie Aqua Fixable Viability Kit (BioLegend) and antibodies detecting membrane markers CD14, CD83, CD86, CD163, CD206 and HLA-DR (listed in Supplementary Table 11). The antibody panel was optimized using isotype and fluorescence minus one (FMO) controls and a built-in compensation algorithm. Flow cytometry was performed using a Fortessa X-20 Analyzer (BD Biosciences) operated by FACSDiva 8.3 software. FlowJo v.10.6.1 software (BD Biosciences) was used for data analysis.

### Administration of mRNA LNPs and mRNA to in vitro-cultured cells

The 0.5 × 10^6^–1 × 10^6^ cells were seeded the day before on a six-well plate in medium as described above. The required amount of vaccine mRNA in original LNP formulation was diluted in 150 µl of Opti-MEM medium (Thermo Fisher Scientific) at room temperature, and after 10 min was added to the cells in a drop-wise manner and gently mixed. Cells were collected at time points specified for the individual experiments.

In the dose-dependent experiment, cells were transfected with varying volumes of original LNPs, with the quantity of RNA required (0.2 µg, 1 µg, 2 µg, 5 µg or 10 µg) diluted in OPTI-MEM medium. For the control cells (those not treated with the specified agent), an appropriate volume of OPTI-MEM was added. All transfections of purified vaccine mRNAs were performed with Lipofectamine MessengerMAX (Thermo Fisher Scientific), according to the manufacturer’s instructions. Cells were collected at time points specified for the individual experiments for subsequent analyses.

### Cell fractionation

Macrophages were fractionated using the Subcellular Protein Fractionation Kit for Cultured Cells (Thermo Fisher Scientific, 78840), according to the manufacturer’s instructions. All buffers used were supplemented with RiboLock (Thermo Fisher Scientific, EO0382) to a final concentration of 1 U µl^−1^. The collected fractions for RNA isolation were immediately combined with TRIzol LS, incubated for five minutes at room temperature and subsequently frozen at –80 °C. For western blot analyses, samples were frozen in liquid nitrogen.

### Gene silencing by siRNA

siRNA-mediated knockdowns in hMDMs were performed using validated stealth siRNAs: HSS124645, HSS124646 and HSS183139 (all for *TENT5A*). Knockdowns in mBMDMs were performed using validated stealth siRNAs: MSS279478 (*Tent3a*; also known as *Tut4* and *Zcchc11*), MSS279479 (*Tent3a*), MSS210722 (*Tent3b*; also known as *Tut7* and *Zcchc6*), MSS210723 (*Tent3b*), MSS200078 (*Tent2*; also known as *Papd4*), MSS200079 (*Tent2*), MSS209990 (*Tent4a*; also known as *Papd7*), MSS209991 (*Tent4a*), MSS277931 (*Tent4b*; also known as *Papd5*), MSS277932 (*Tent4b*), MSS220103 (*Fndc3a*) and MSS291871 (*Fndc3b*).

Transfections were performed with Lipofectamine RNAiMAX (Invitrogen) according to the manufacturer’s instructions, as previously described^24,33^. Recommended negative controls (Invitrogen, 12935100) were used.

### General molecular biology techniques

#### Western blots

An equal number of cells were lysed in PBS supplemented with 0.1% NP40, protease inhibitors and viscolase (final concentration 0.1 U ml^−1^; A&A Biotechnology, 1010-100) for 30 min at 37 °C with shaking at 1,200 rpm. Then 3× SDS sample buffer (187.5 mM Tris-HCl pH 6.8, 6% SDS, 150 mM DTT, 0.02% bromophenol blue, 30% glycerol and 3% 2-mercaptoethanol) was added and samples were boiled for 10 min. Samples were resolved on 12–15% SDS–PAGE gels and then proteins were wet-transferred to Protran nitrocellulose membranes (GE Healthcare) at 400 mA at 4 °C for 1.5 h in 1× transfer buffer (25 mM Tris base, 192 mM glycine and 20% methanol (v/v). Next, the proteins were visualized by staining with 0.3% w/v Ponceau S in 3% v/v acetic acid and digitalized. Membranes were blocked by incubation in 5% milk in TBST buffer for 1 h followed by overnight incubation at 4 °C with specific primary antibodies (listed in Supplementary Table 11) diluted 1:2,500 (spike protein, CD80, GRP94, HA, SSR1, PERK, calreticulin and PDI) or 1:5,000 (α-tubulin, β-actin, actinin, eIF2a and GAPDH). Membranes were washed three times in TBST buffer for 10 min each and incubated with HRP-conjugated secondary antibodies (anti-mouse (Millipore, 401215) diluted 1:5,000 and anti-rabbit (Millipore, 401393) diluted 1:5,000) for 2 h at room temperature. Membranes were washed three times in TBST buffer and proteins were visualized using X-ray films or ChemiDoc (BioRad) or iBright (Thermo Fisher Scientific) imaging systems. Blots were quantified using Multi Gauge v.3.0 (Fujifilm Life Sciences). Unprocessed scans are shown as source data.

#### RNA isolation

Total RNA was isolated from cells or vaccine samples with TRIzol reagent or TRIzol LS reagent (both from Thermo Fisher Scientific), respectively, according to the manufacturer’s instructions, dissolved in nuclease-free water and stored at −20 °C (short term) or −80 °C (long term). RNA from frozen muscles and lymph nodes was isolated by tissue homogenization in TRI reagent (Sigma, T9424) pre-heated to 60 °C, using Omni Tissue Homogenizer equipped with a 7 × 115-mm Saw Tooth (Fine) Generator Probe. Homogenous mixtures were further processed according to the manufacturer’s instructions. For qRT–PCR, RNA-seq and DRS library preparation, the RNA was treated with TURBO DNase (Thermo Fisher Scientific, AM1907; or Invitrogen, AM2238). To assess the integrity of isolates, each RNA sample after DNAse treatment was analysed with the Agilent 2200 TapeStation system, using Agilent High Senitivity RNA ScreenTape (Agilent, 5067-5579).

RNA from cells acquired by fluorescence-activated cell sorting (FACS) was isolated using the Arcturus PicoPure RNA Isolation Kit (Applied Biosystems, KIT0204) following guidelines for RNA isolation from cell pellets with on-column DNAse treatment (Qiagen, 79256), with some modifications. For qRT–PCR analyses, we sorted 1,000 cells of each type, from separate mice, directly to 100 µl extraction buffer. In the case of cDNA sequencing, we sorted 20,000 cells of each type, pooled from three mice, directly to 250 µl extraction buffer. During the precipitation step, we used a 1:1 (v:v) 70% ethanol:extraction buffer ratio. RNA was eluted in 15 µl elution buffer in every case.

### qRT–PCR for mRNA-1273 quantification and relative gene expression

cDNA was synthesized using 500 ng of DNAsed total RNA as a template with SuperScript III Reverse Transcriptase (Invitrogen, 18080093) according to the manufacturer’s instructions. The final cDNA concentration was kept at 2.5 ng µl^−1^ (converted from total RNA). In the case of RNA from FACS-sorted cells, 11 µl of the entire eluate was used for cDNA synthesis.

To determine the concentration of Moderna’s mRNA-1273 in mouse tissues or FACS-sorted cells, a custom-made TaqMan probe together with TaqMan Gene Expression Master Mix (Applied Biosystems, 4369016) was used and normalized with a pre-designed gene-expression assay for β-actin (Applied Biosystems, Mm01205647_g1). The reaction mix was contained in a total volume of 10 µl with 1× concentration of master mix and gene-expression assay; 5 ng cDNA was used per reaction (converted from total RNA). In the case of FACS-sorted cells, 2 µl of 1.5× diluted cDNA was used, which roughly translates to 60 cells per 384-well plate, assuming 100% recovery of material during RNA isolation and 100% efficiency of cDNA synthesis. A thermal cycling program for TaqMan Gene Expression Master Mix was used as instructed by the manufacturer.

To estimate the relative expression of *Tent5a* and *Tent5c* at injection sites or in FACS-sorted cells, and the levels of mRNA-1273 in HEK293T and A549 cells with varying amounts of vaccine, we used Platinum SYBR Green qPCR SuperMix-UDG (Invitrogen, 11733046) following the general protocol for ABI instruments recommended by the manufacturer.

For all qRT–PCR analyses, the QuantStudio 5 Real-Time PCR System, 384-well (Applied Biosystems, A28140) was used.

#### Custom TaqMan probe design and absolute quantification of vaccines

The primer blast algorithm was used to find a unique amplicon for mRNA-1273 in the mouse transcriptome (Refseq mRNA). Within the amplified region, a 15-mer of optimal GC content (53.3%) was selected as the target site for probe hybridization. We selected FAM dye and MGB-NFQ quencher as 5′ and 3′ modifications for the probe. The probe was synthesized by the Thermo Fisher Scientific Custom TaqMan probes service. To test the specificity and sensitivity of the designed assay, we performed a serial dilution experiment in which we spiked 500 ng of DNAsed mouse total RNA with 50 ng of DNAsed mRNA-1273 and performed cDNA synthesis. Then we performed 10× serial dilutions of that mix with unspiked mouse cDNA of the same concentration. qRT–PCR analysis showed that the designed assay is specific and can detect up to 10 ag of mRNA-1273 per ng of total RNA, which is equivalent to roughly 25 molecules. Similarly, we prepared a standard curve with 10× serial dilutions of mRNA-1273 to estimate the absolute concentration of vaccine in FACS-acquired cells. The concentration of vaccine before cDNA synthesis was determined with the Agilent 2200 TapeStation system using Agilent High Sensitivity RNA tape. Five nanograms of vaccine was added to 500 ng yeast total RNA and cDNA was synthesized; the final sample volume was 200 µl. Next, we performed eight serial dilutions (10×) using yeast cDNA of 2.5 ng µl^−1^ concentration. Recorded *C*_t_ values for each concentration served as data points for plotting an exponential curve, and we used this equation to convert *C*_t_ values to molecules of mRNA-1273, assuming its molecular mass is 1321.81 kDa. Next, the calculated number of molecules was normalized to the number of cells in a 384-well plate (translated from the % of eluate used for cDNA synthesis, assuming 100% recovery of cells during sorting and RNA isolation as well as cDNA synthesis efficiency).

#### qRT–PCR for ER stress assessment

The relative expression of genes associated with ER stress, we assessed *CHOP* (also known as *DDIT3*), *GRP94* (*HSP90B1*), *PERK* (*EIF2AK3*) and *XBP1* mRNA splicing in mRNA-1273-transfected hMDMs (wild-type and *TENT5A* knockdown) using the Platinum SYBR Green qPCR SuperMix-UDG (Invitrogen, 11733046), following the general protocol for ABI instruments recommended by the manufacturer. Gene expression for each sample was normalized to *ACTB*.

Primers and probe sequences are listed in Supplementary Table 12.

### 3′-RACE sequencing

To examine the 3′-UTR and terminal sequence of the mRNA-1273, RNA was freshly isolated from the vaccine sample vial. Then, 1 µg of vaccine mRNA was ligated to 20 pmol RA3_7N adaptor: 5rApp/CTGACNNNNNNNTGGAATTCTCGGGTGCCAAGG/3ddC with 10 U of T4 KQ227 RNA ligase 1 (NEB, M0204S) in the presence of 20 U RNase OUT (Thermo Fisher Scientific, 10777019), 1× T4 RNA Ligase Reaction Buffer (NEB, M0204S), 1 mM ATP and 20% PEG8000 in a total reaction volume of 20 µl at 25 °C for 4 h. Ligase was inactivated at 65 °C for 20 min. The ligation product was purified with a 0.8× ratio of KAPA Pure Beads and eluted with 15 µl RNase-free water, according to the manufacturer’s protocol. The cleaned ligation product was subjected to reverse transcription with 40 pmol of Illumina index adapter: 5′-CAAGCAGAAGACGGCATACGAGATATCAGTGTGACTGGAGTTCCTTGGCACCCGAGAATTCCA-3′ with SuperScript III (Thermo Fisher Scientific, 18080093), 1× First Strand Buffer (Thermo Fisher Scientific, 18080093), 0.25 mM dNTP mix, 5 mM DTT and 20 U RNAse OUT (Thermo Fisher Scientific, 10777019). The reaction mix was incubated at 45 °C for 1 h and 70 °C for 20 min in a thermocycler. The reverse transcription product was cleaned with a 1× ratio of KAPA Pure Beads and eluted with 19 µl RNase-free water. Prepared cDNA was diluted in a 1:3 proportion. In the library amplification step, 0.5 µl of cDNA was mixed with 0.4 pmol of gene-specific starter 5′-AATGATACGGCGACCACCGAGATCTACACGTTCAGAGTTCTACAGTCCGACGATCAGAAGGAGATCGATCGGCTG-3′, 0.2 pmol of RP universal starter 5′-CAAGCAGAAGACGGCATACGAGAT-3′, 0.25 mM dNTP, Phusion High-Fidelity DNA Polymerase (Thermo Fisher Scientific, F530S), 1× Phusion HF Buffer (Thermo Fisher Scientific, F530S) and 3% DMSO. For amplification, a standard Phusion program was used at 65 °C for annealing and 25 cycles. The 50 µl of PCR product was separated in 2.5% agarose gel. A band of 450 bp was cut out from the gel and purified with Gel-OUT (A&A Biotechnology, 023-50) according to the kit protocol. The library was cleaned twice with a 1.0× ratio of KAPA Pure Beads. TapeStation analysis of the sample was performed as a quality control. The library was sequenced on an Illumina NovaSeq 6000 sequencer.

### Analysis of data from 3′-RACE sequencing

RA37_N adapter sequence was trimmed from the R2 read (containing poly(A) tail) with cutadapt^34^ (options -g CCTTGGCACCCGAGAATTCCANNNNNNNGTCAG –discard-untrimmed). Then only reads containing poly(A) tail were identified with cutadapt (options -a TTTTTT --discard-untrimmed --fasta). Obtained sequences were reverse complemented using fastx_reverse_complement from the fastx toolkit (0.0.13) and loaded into R with the BioStrings package. To get rid of unwanted trimming artefacts, sequences with length between 0 and 10 were chosen, four A letters (representing the poly(A) tail) were pasted before each sequence for visualization purposes and a sequence logo representing the nucleotide composition of the 3′ end of mRNA-1273 was produced with the ggseqlogo package^35^.

### Analysis of translation in vitro

For in vitro translation, the Retic Lysate IVT Kit (Invitrogen, AM1200M) was used. Reactions were assembled using the default buffer suggested by the producer. The translation mix (–met) was supplemented with unlabelled methionine to a final concentration of 50 μM. Either 1 μg or 3 μg of purified capped RNA was used as template in each reaction. The reaction was run for 180 min. Spike protein was detected in the reaction mixture using ELISA.

### Preparation of standards with predefined poly(A) lengths

Spike-in RNAs were IVT from a set of double-stranded DNA fragments. Templates for transcription were prepared in two consecutive PCR reactions. First, a desired fragment of Renilla luciferase from pRL5Box plasmid was amplified using RLuc_F1/R1-specific primers containing an overhang common for all primers used in the second round of PCR (Supplementary Table 12). PCR products were verified through gel electrophoresis. Correct amplicons were used as templates in the second PCR reaction with RLuc_T7_F2 primer, hybridizing to the overhang sequence from RLuc_F1 and containing the T7 promoter, and backward primer RLuc_Ax_R2, hybridizing to the overhang sequence from RLuc_R1 oligo and introducing a poly(A) tail of a defined length (from 10 to 120 A). The resulting PCR products were assessed and purified by gel electrophoresis. The in vitro transcription reaction was performed at 37 °C for 1.5 h in a 50-µl reaction volume containing 600 pmol T7 template, 10 µl of 5× transcription buffer (200 mM Tris-HCl, 30 mM MgCl_2_, 10 mM spermidine and 50 mM NaCl), 5 µl of rNTP mix (20 mM each), 5 µl of 100 mM DTT, 0.5 µl of 1% Triton X-100, 80 U ribonuclease inhibitor and 100 U T7 RNA polymerase. Then, DNA template was removed with TURBO DNase (Ambion) for the next 15 min. Spike-in RNAs were phenol–chloroform extracted, precipitated, visually assessed by denaturing electrophoresis, purified on RNA purification beads and used as controls in DRS runs. In each DRS run, a mixture of spike-ins representing RNAs with poly(A) tail of defined lengths (10 A, 15 A, 30 A, 45 A, 60 A, 90 A and 120 A) was included.

### EGFP and reporter mRNA synthesis

The EGFP and reporter mRNAs were IVT from plasmid template and double-stranded DNA fragments, respectively. Template for transcription of EGFP mRNA was prepared as follows: the high-RSCU EGFP^32^ CDS was purchased from Invitrogen and cloned into the plasmid vector pJAZZ (Lucigen) using BigEasy-TSA competent bacteria (Lucigen), providing it with the 5′-UTR and 3′-UTR of the mRNA-1273, a poly(A) tail of 90 A and the T7 promoter Φ6.5 (TAATACGACTCACTATAGGG). The plasmid was digested at the poly(A) tail terminus (PaqCI, NEB) and was verified to be completely digested through agarose gel electrophoresis. DNA template was purified by buffered phenol–chloroform extraction and ethanol precipitation. Templates for transcription of reporter mRNAs were prepared in a PCR reaction (Supplementary Note 6 and Extended Data Fig. 8a,b) as follows: full-length CDSs of *Plasmodium falciparum* circumsporozoite (CS) protein (PfCSP_3D7; NCBI reference sequence: XP_001351122.1; Extended Data Fig. 7h), OVA (sequence available at https://www.trilinkbiotech.com/) and Zika virus protein E (ZIKVE; NCBI reference sequence: YP_002790881.1) with UTRs from either mRNA-1273 or BNT162b2 were synthesized using the GeneArt service (Thermo Fisher Scientific). For OVA and ZIKVE constructs, an N-terminal ER signal peptide sequence was added (MFVFLVLLPLVSSQCV). All CDSs were optimized for expression in mouse cells using software from Benchling (https://www.benchling.com/). DNAs were PCR-amplified using primers complementary to UTRs with overhangs introducing the RNA T7 promoter Φ6.5 (TAATACGACTCACTATAGGG) at the 5′ end and a poly(A) tail from either mRNA-1273 or Pfizer BNT162b2 and either with or without a TCTAG pentamer at the 3′ end (Supplementary Table 12). In the case of OVA DNAs, an additional set was PCR-amplified as described above, but with primers with a point mutation in the T7 promoter sequence, allowing for efficient cotranscriptional RNA capping (TAATACGACTCACTATAAGG). PCR products were verified through agarose gel electrophoresis and purified with KAPA Pure Beads (KAPA Biosystems).

The composition of the in vitro transcription reaction was: 40–80 ng µl^−1^ of DNA template, transcription buffer (40 mM Tris-HCl, 26 mM MgCl_2_, 10 mM NaCl, 2 mM spermidine and 10 mM DTT), T7 RNA polymerase^36^ (0.04 µg µl^−1^, in-house prepared), 5 mM ATP/CTP (Thermo Fisher Scientific), 5 mM N^1^meΨTP (Advent Bio) or UTP (Thermo Fisher Scientific) and 4 mM GTP (in the case of EGFP and OVA reporter mRNAs intended for cotranscriptional capping; Thermo Fisher Scientific) or 5 mM GTP (in the case of all other reporter mRNAs; Thermo Fisher Scientific). The reaction mixtures were supplemented with 1 U µl^−1^ RiboLock RNase inhibitor (Thermo Fisher Scientific), 0.002 U µl^−1^ inorganic pyrophosphatase (Thermo Fisher Scientific) and, in the case of EGFP and OVA reporter mRNAs intended for cotranscriptional capping, 10 mM of the trinucleotide Cap1 analogue m_7_GpppA_m_pG^37^ (in-house prepared). The reactions were performed for 120 min at 37 °C. In vitro transcription products were verified through denaturing agarose gel electrophoresis and FPLC-purified using a 0.2 ml GoPure column with POROS Oligo (dT)25 Affinity Resin (Thermo Fisher Scientific) according to the manufacturer’s protocol. The concentration of purified mRNAs was determined using UV absorbance measurements. The purity and integrity of the RNA preparations were determined by the automated electrophoresis system (TapeStation 2200, Agilent Technologies; Extended Data Fig. 8c). The reporter RNAs (except EGFP and cotranscriptionally capped OVA) were then provided with Cap1 using the Vaccinia Capping System (NEB) and Cap 2′-*O*-methyltransferase (NEB) according to the manufacturer’s protocol with the replacement of the manufacturer’s VCE with the in-house prepared one^38^. Capped mRNAs were purified with KAPA Pure Beads (KAPA Biosystems). The purity and integrity of the mRNA preparations were determined by automated electrophoresis (TapeStation 2200, Agilent Technologies; Extended Data Fig. 7d).

### Inosine tailing and nanopore sequencing

Two micrograms of RNA isolated from a sample of mRNA-1273 vaccine was denatured in the presence of 20 U RNAse OUT (10777019, Thermo Fisher Scientific) at 65 °C for 3 min, and immediately placed on ice. An inosine (I)-tailing reaction was performed with 0.5 mM ITP (inosine triphosphate), 1× NEB 2.0 buffer (B7002, NEB) and 2 U poly(U) polymerase (M0337S, NEB) at 37 °C for 45 min, and was terminated by snap-freezing in liquid nitrogen. I-tailed RNA was cleaned twice on KAPA Pure Beads (7983298001, Roche) in a 1× ratio and used for DRS library preparation. I-tailed samples require ligation of a special adaptor RTA_C10, which contains 10 cytosines at the 3′ end: 5′-GAGGCGAGCGGTCAATTTTCCTA AGAGCAAGAAGAAGCCCCCCCCCCCC-3′. To make the I-tailing-specific RTA adaptor, we followed the ONT Direct RNA Sequencing—Sequence-Specific protocol; 0.5 µg of I-tailed mRNA-1273 was taken to the first ligation step of DRS library preparation. Library preparation was performed as described in the Direct RNA Sequencing (ONT, SQK-RNA002) protocol.

### Nanopore sequencing

#### DRS

DRS was performed as described previously^3^. For raw vaccine isolate, up to 0.5 µg of RNA was used for library preparation. For RNA isolates obtained from cell cultures or mouse tissues, 3.5–5 µg of total mRNA was mixed with 50–200 ng oligo-(dT)_25_-enriched mRNA from *Saccharomyces cerevisiae* and standards with predefined poly(A) lengths, and was processed with a Direct RNA Sequencing Kit (SQK-RNA002, ONT) according to the manufacturer’s instructions.

#### cDNA sequencing

For cDNA sequencing, 400 ng of total RNA was used for library preparation using the cDNA-PCR Sequencing Kit (SQK-PCS111) or PCR-cDNA Barcoding Kit (SQK-PCB111.24 or SQK-PCB114.24) according to the manufacturer’s instructions. Libraries were amplified with 14 cycles of PCR reaction, and the DNA concentration was measured using the Qubit 1× dsDNA High Sensitivity Kit (Invitrogen, Q33231). Then, if multiplexed, they were diluted and mixed equally to reach a final concentration of 60–100 fmol.

cDNA libraries for sequencing with an additional amplification step of mRNA-1273 (applicable for samples with an expected low prevalence of vaccine mRNA, such as cells sorted from vaccine injection sites) were prepared using the standard SQK-PCS111 protocol with the following modification. After synthesis of the first cDNA strand, samples from the reverse transcription reaction were pre-amplified in a 12-cycle PCR reaction with RTP and SSPII_Mod_2 (specific for mRNA-1273, with UMI sequence TTTCTGTTGGTGCTGATATTGCTTTVVVVTTVVVVTTVVVVTTVVVVTTTCCACCGACAACACCTTCGTGAGCGG) primers using the LongAmp Hot Start Taq Master Mix. The reaction products were then purified on Kappa beads, washed with SFB buffer, eluted with H_2_O and used for the subsequent PCR reaction with barcoded primers, performed according to the SQK-PCB111-24 protocol (14 cycles, 5 min amplification). Finally, cDNA concentrations were measured with Qubit and 12 libraries containing a total of around 60 ng cDNA were used for ligation with the RAPT adapter and sequenced.

Sequencing was performed using R9.4 (for DRS and v.11 DNA chemistry) or R10.4 (for v.14 DNA chemistry) flow cells on a MinION device (ONT) and with the Flow Cell Priming Kit (EXP-FLP002). Raw data were basecalled using Guppy (ONT) in the case of DRS, or dorado (ONT) in the case of cDNA sequencing. Raw sequencing data (fast5 or pod5 files) were deposited at the European Nucleotide Archive (ENA, project PRJEB53190; Supplementary Table 13).

### Determining poly(A) lengths from DRS

Basecalled nanopore reads were mapped to the respective transcriptome references (Gencode 26 or Gencode 38 for mouse and human samples, respectively) using Minimap2 2.17 with options -k 14 -ax map-ont –secondary=no, and processed with Samtools 1.9 to filter out supplementary alignments and read mapping to reverse strand (Samtools view -b -F 2320). The poly(A) tail lengths for each read were estimated using the nanopolish 0.13.2 polya function^23^.

For the analysis of mRNA-1273-originating reads, the nanopolish polya algorithm was modified (Supplementary Note 2) to (1) include unmapped reads enabling the analysis of poly(A) lengths for sDTW-identified reads (Supplementary Note 1) and (2) detect mΨCmΨAG at the 3′ end of the poly(A) tail and report its presence in the output.

For the analysis of BNT162b2-originating reads, the nanopolish polya algorithm was modified analogously (Supplementary Note 3) to (1) include unmapped reads enabling the analysis of poly(A) lengths for sDTW-identified reads and (2) detect two poly(A) segments interleaved with a 10-nt linker and report their lengths in the output.

### Determining poly(A) lengths from cDNA sequencing

For cDNA barcoded libraries (SQK-PCB111-24), raw sequencing data were basecalled with either dorado 0.5.3 or dorado 0.7.0 with parallel mapping to relevant reference (with option –secondary=no to exclude secondary alignments) and poly(A) detection turned on with the option –estimate-poly-a. Basecalled bam files were demultiplexed with dorado demux and sorted with samtools sort. Poly(A) lengths for each sequencing read were extracted from the pt:i tag (in the basecalled bam file) with a Python script. In the case of mRNA-1273 samples, the read sequence was also used for identifying the terminal pentamer from the basecalled sequence using regular expressions.

### Statistical analysis of poly(A) lengths

*P* values for each transcript were estimated using the Kruskal–Wallis test and adjusted for multiple comparisons using the Benjamini–Hochberg method. Transcripts were considered as having a significant change in poly(A) tail length if the adjusted *P* value was less than 0.05 and if there were at least 20 supporting reads for each condition.

### Differential expression analyses

Illumina RNA-seq reads were mapped to the mouse reference genome (GRCm38, ENSEMBL, release 94) using the STAR aligner (v.2.7.6a)^39^. Read counts were assigned to genes using featureCounts from the Subread package (v.2.0.1) with options -Q 10 -p -B -C -s 2 -g gene_id -t exon and the respective annotation file (Gencode v.M25). Multimappers and reads overlapping multiple features were not counted. Nanopore read counts were derived from the nanopolish polya output files, used for the analyses of poly(A) tail lengths. Reads assigned for each gene were summarized and obtained counts were used for subsequent analyses.

Differential expression analysis was performed with the DESeq2 (v.1.22) Bioconductor package^40^, using a likelihood ratio test for data from time-course experiments. Pair-wise comparisons of each time point were calculated with the Wald test, with log fold change values shrunk using the apeglm method. Genes with similar expression patterns were clustered with hierarchical clustering using the ward.D method and *z*-scored expression values for each gene. Heat maps were drawn with the ComplexHeatmap package^41^. g:Profiler^42^ or ClusterProfiler were used for Gene Ontology (GO) enrichment analysis. Genes were assigned as immune response genes (Fig. 4c and Supplementary Tables 6 and 7) if they were annotated to GO (Biological Process) terms GO:0140374, GO:0090714, GO:0034340, GO:0034341, GO:0034342, GO:0045087 or GO:0035455.

### Codon usage analysis

Codon usage indices were analysed using the coRdon (https://github.com/BioinfoHR/coRdon) and cubar (https://github.com/mt1022/cubar) R packages. To compute the codon adaptation index, a reference set of 500 highly expressed genes in mBMDMs, based on a previous study^7^, was provided. For calculating the effective number of codons, codon bias or preference within the vertebrate context was used. The frequency of optimal codons was determined on the basis of the occurrence frequency of each amino acid in reference sequences. The mRNA vaccines BNT162b2 and mRNA-1273 were compared against the SARS-CoV-2 spike protein reference sequence from GenBank (accession: NC_045512.2:21563-25384), mouse endogenous transcripts (protein-coding), transcript groups selected by GO terms (for example, associated with the membrane or endoplasmic reticulum) and potential substrates of TENT5A and TENT5C, selected as per a previous study^7^, as well as IVT reporter sequences for OVA, PfCSP and ZikVE. GC content and other sequence features were assessed using functions available from Bioconductor and the GenRCA Rare Codon Analysis Tool (https://www.genscript.com/tools/rare-codon-analysis), providing mouse as the host organism and the Kazusa database as the codon usage reference. The results of the analyses were visualized with ggplot2.

### Statistics and reproducibility

No statistical methods were used to predetermine sample sizes. Statistical analysis was performed on data from two or more biologically independent experimental replicates using the R environment or Prism 6 software unless otherwise stated. The statistical tests used in each instance are provided in the figure legends.

Data from the analyses of poly(A) tail length are presented as scatter dot plots, as indicated in the figure legends, and individual data points are shown. The median of poly(A) length is shown on the plots (as a horizontal bar and a numeric value) for mRNA populations with processed poly(A) tails (that is, without the terminal pentamer in the case of mRNA-1273, OVA, PfCSP and ZikVE reporters, or for all the reads in the case of BNT162b2); the mean value would be biased because poly(A) lengths do not follow a normal distribution. Plots indicate fractions of reads with elongated (blue-shaded) or shortened (red-shaded) tails. Classification thresholds (deadenylation and re-adenylation) were estimated as values close to the 0.2 and 0.8 quantiles (respectively) from crude vaccine RNA (with pentamer in the case of mRNA-1273). In the case of DRS data, the thresholds were set at 85 and 115 for mRNA-1273 and 95 and 125 for BNT162b2. For cDNA sequencing analysis with dorado, which usually gives shorter estimates of poly(A) tail length, the thresholds were set at 80 and 110 for mRNA-1273 tails and 50 and 80 for BNT162b2 tails (analysis was performed without additional segmentation, so the length of the last poly(A) segment was reported).

Most of the experiments were repeated at least twice, leading to comparable results, with the exception of the mRNA-1273 treatment of A549 cells and the viability assays, which were repeated once. Samples with clear technical failures during tissue collection, cell isolation, processing or data collection were excluded from the analyses.

### Reporting summary

Further information on research design is available in the Nature Portfolio Reporting Summary linked to this article.

## Online content

Any methods, additional references, Nature Portfolio reporting summaries, source data, extended data, supplementary information, acknowledgements, peer review information; details of author contributions and competing interests; and statements of data and code availability are available at 10.1038/s41586-025-08842-1.

## Supplementary information

Supplementary Information This file contains Supplementary Notes 1–7, Supplementary References, descriptions of the Supplementary Tables, Supplementary Table 14 and Supplementary Figures 1–7.

Reporting Summary

Supplementary Tables This file contains Supplementary Tables 1–6, 10, 12 and 13.

Supplementary Table 7 Differential expression of genes in hMDMs upon mRNA-1273 delivery

Supplementary Table 8 GO (Biological Process) terms for WT mBMDMs upon mRNA-1273 delivery.

Supplementary Table 9 GO (Biological Process) terms for hMDMs upon mRNA-1273 delivery.

Supplementary Table 11 List of antibodies used in this work

## Source data

Source Data Figs. 1 and 2 and Source Data Extended Data Figs. 1, 2, 6 and 10.

## Data Availability

Nanopore direct RNA sequences have been deposited at the European Nucleotide Archive (ENA) under accession number PRJEB53190 (all sequencing datasets with corresponding ENA accession numbers are also listed in Supplementary Table 13). Illumina sequencing data have been deposited at the Gene Expression Omnibus (GEO) under accession number GSE233059. Raw data underlying the figures are provided as source data or supplementary files or are available from the corresponding authors upon reasonable request. Source data are provided with this paper.

## Code availability

The dynamic time warping script is available at https://github.com/LRB-IIMCB/DTW_mRNA-1273. Nanopolish polya for identification of mΨCmΨAG is available at https://github.com/LRB-IIMCB/nanopolish_mRNA-1273_BNT162b2.
