# Supplementary material for: Re-adenylation by TENT5A enhances efficacy of SARS-CoV-2 mRNA vaccines
Source: Nature. 2025 Apr 16;641(8064):984–92. doi: 10.1038/s41586-025-08842-1 (PMC12095053; doi:10.1038/s41586-025-08842-1)

nature portfolio

https://doi.org/10.1038/s41586-025-08842-1

Supplementary information

Re-adenylation by TENT5A enhances efficacy of SARS-CoV-2 mRNA vaccines

In the format provided by the authors and unedited

Supplementary Information for:

Re-adenylation by TENT5A enhances efficacy of SARS-CoV-2 mRNA vaccines

Paweł S Krawczyk[1], Michał Mazur[1,*], Wiktoria Orzeł[2,1*], Olga Gewartowska[3,*], Sebastian Jeleń[1,2], Wiktor Antczak[1,2], Karolina Kasztelan[1], Aleksandra Brouze[1,2], Katarzyna Matylla-Kulińska[1,2], Natalia Gumińska[1], Bartosz Tarkowski[1], Ewelina P. Owczarek[1], Kamila Affek[1], Paweł Turowski[4], Agnieszka Tudek[5], Małgorzata Sroka[6], Tomasz Śpiewla[7], Monika Kusio-Kobiałka[1], Aleksandra Wesołowska[8], Dominika Nowis[9] , Jakub Golab[10], Joanna Kowalska[4], Jacek Jemielity[11], Andrzej Dziembowski[1,12,#], and Seweryn Mroczek[2,1,#]

[1] Laboratory of RNA Biology, International Institute of Molecular and Cell Biology, 4 Ks. Trojdena, 02-106 Warsaw, Poland.

[2] Institute of Genetics and Biotechnology, Faculty of Biology, University of Warsaw, 5a Pawińskiego, 02-106, Warsaw, Poland

[3] Genome Engineering Facility, International Institute of Molecular and Cell Biology, 4 Ks. Trojdena, 02-106 Warsaw, Poland.

[4] ExploRNA Therapeutics, 101 Żwirki i Wigury, 02-089, Warsaw, Poland

[5] Institute of Biochemistry and Biophysics, 5A Pawińskiego, 02-106 Warsaw, Poland

[6] Laboratory of Protein Structure, International Institute of Molecular and Cell Biology, 4 Ks. Trojdena, 02-106 Warsaw, Poland.

[7] Faculty of Physics, University of Warsaw, Pasteura 5, 02-093, Warsaw, Poland

[8] Department of Medical Biology, Medical University of Warsaw, Litewska 14/16, 00-575, Warsaw, Poland

[9] Laboratory of Experimental Medicine, Medical University of Warsaw, 5 Nielubowicza, 02-097, Warsaw, Poland

[10] Department of Immunology, Medical University of Warsaw, 5 Nielubowicza, 02-097, Warsaw, Poland

[11] Centre of New Technologies, University of Warsaw, Banacha 2c, 02-097, Warsaw, Poland

[12] Department of Embryology, Faculty of Biology, University of Warsaw, 1 Miecznikowa, 02-096 Warsaw, Poland

* These authors contributed equally to this work

# corresponding authors

Table of Contents

Supplementary Note 1 Direct RNA sequencing of mΨ-containing mRNAs 3

Supplementary Note 2 Analysis of mΨCmΨAG-containing poly(A) tails of mRNA-1273 with Nanopore Direct RNA Sequencing 5

Supplementary Note 3 Analysis of composite poly(A) tail from BNT162b2 vaccine with Nanopore Direct RNA Sequencing 6

Supplementary Note 4 Cytotoxicity of mRNA vaccines 7

Supplementary Note 5 Flow cytometry analysis of muscle-infiltrating immune cells following mice vaccination with mRNA-1273 8

Supplementary Note 6 Design of reporter mRNAs encoding OVA, PfCSP and ZikVE 10

Supplementary Note 7 Comparison of mRNA-1273 and BNT162b2 vaccines and mRNA reporters used in this work 11

Supplementary References 13

Supplementary Tables description 16

Supplementary Table 14 18

Supplementary Figures 19

Supplementary Note 1 Direct RNA sequencing of mΨ-containing mRNAs

Therapeutic mRNA resembles normal mRNA, having both a cap structure and poly(A) tail[1], but it is generated through *in vitro* transcription, usually by T7 polymerase on a DNA template. A breakthrough in the development of RNA therapeutics came with the discovery that replacement of uridine with N1-methyl-pseudouridine (mΨ) decreases the innate immune response and enhances mRNA stability[2–5]. Although most currently synthesized mRNA therapeutics contain this modification, exceptions show it is not essential[6,7].

Replacing uridine with mΨ in mRNA therapeutics presents additional challenges. Direct RNA sequencing (DRS, Oxford Nanopore Technologies[8]) is based on detecting electric current during the passage of single ssRNA molecules through a protein pore. However, mΨ in mRNA therapeutics perturbs the current signal recorded during DRS and can result in imprecise translation into a sequence (basecalling)[9]. Our experiments confirmed that mΨ indeed affects the basecalling process (Extended Data Fig. 1a,b). Only 35.74% of reads aligned to the reference vaccine sequence and those aligned reads had only 74.1% identity to the reference. Therefore, the data analysis pipeline is unsuitable for mΨ modified mRNAs.

Identification of vaccine-originating reads using subsequence Dynamic Time Warping

To enhance our ability to identify desired reads, we have developed a subsequence Dynamic Time Warping (sDTW) approach (Extended Data Fig. 1c) to detect ionic current signatures unique to the 3' end of mRNA-1273 or BNT162b2. This method, in principle, is similar to targeted nanopore sequencing[10] and is independent of basecalling and mapping.

In detail, the DTAIDistance library[11] (version 2.3.5) was used to compare raw signals from nanopore sequencing. Reference signals were obtained from the reads from the DRS run of crude mRNA-1273 (read id: df408ab3-7418-4ee4-9a67-92743257b20a) or BNT162b2 (read id: 045d4b9c-9222-425b-a7a4-9abb3597f9dd) material, which were giving good coverage of 3'end of either mRNA-1273 or BNT162b2 reference. The 5000 data points, covering part of poly(A) tail (1000 or 500 data points for mRNA-1273 and Pfizer, respectively) and 3' end of transcript (4000 or 4500 data points for mRNA-1273 and Pfizer, respectively) were selected for further processing (Extended Data Fig. 1c). Such fragment of raw current readout was smoothed using Savitzky-Golay filter (savgol_filter function from Scipy Python library), with window length set to 51 and polynomial order set to 3, and normalized using zscore function from stats python package. Raw sequencing data were read from fast5 files using ONT Fast5 API (version 4.0.0), first 20000 data points (which usually cover the 3' end of sequenced RNA) were selected, then smoothed and normalized in the same way as the reference signal. Then, the subsequence_alignment function from the DTAIdistance library was used to find a region in raw data that best matched the reference signal. As the output for each sequencing read the location of match and

distance score, calculated using the distance_fast function from DTAIDistance library, was reported. The python script for all described operations is available at https://github.com/LRB-IIMCB/DTW_mRNA-1273.

When applied to data obtained from DRS of RNA samples from HEK293T cells incubated with mRNA-1273, distance scores calculated with sDTW clearly separated vaccine-originating reads from the others (Extended Data Fig. 1d). Based on the density plots it was decided that in the subsequent analyses, only reads that were mapping to mRNA-1273 or BNT162b2 reference, or unmapped and having sDTW calculated distance <10 (in case of mRNA-1273) or <11 (in case of BNT162b2) were considered as originating from the respective vaccine.

Compared to mapping alone, the combination of sDTW with mapping enabled the identification of twice as many sequences, both from crude vaccine sample (Extended Data Fig. 1e) as well as from mixed RNA material from cells incubated with the vaccine (Extended Data Fig. 1f), validating the feasibility and potential of this approach. This method was particularly useful for identifying shorter, lower-quality 3'-terminal reads (Extended Data Fig. 1g), which would have been overlooked otherwise. Reads identified with sDTW approach also had the same poly(A) lengths distribution as those identified with the standard mapping-based approach (Extended Data Fig. 2e,g).

Supplementary Note 2 Analysis of mΨCmΨAG-containing poly(A) tails of mRNA-1273 with Nanopore Direct RNA Sequencing

The visual inspection of raw signals obtained from DRS of mRNA-1273 revealed the presence of unexpected perturbation at the 3'end of poly(A) tail (Fig. 1b in the main manuscript), which was further shown to be a mΨCmΨAG pentameric sequence. Consequently, it became necessary to develop a computational method for the automatic detection of this feature, eliminating the requirement for manual analysis.

For this purpose, we modified the original segmentation algorithm that was implemented in the nanopolish-polya program[12]. Additional segment was included between adaptor and poly(A) (Supplementary Fig. 1a), with mixed Gaussian distribution emission, manually estimated using MLE on representative set of raw nanopore signals (similarly as it was done by developers of nanopolish-polya[12]). As a result, 4 additional columns are added to the nanopolish-polya output, containing localization of mΨCmΨAG in the raw signal, its length, summed poly(A) + mΨCmΨAG length, and information if mΨCmΨAG was detected. Detection of mΨCmΨAG was run with the nanopolish polya-moderna function. Additionally, the algorithm was modified to include unmapped reads, enabling analysis of poly(A) lengths for sDTW-identified reads. The source code of the modified nanopolish is available at https://github.com/LRB-IIMCB/nanopolish_mRNA-1273_BNT162b2.

Notably, the presence of mΨCmΨAG is unique and enables easy discrimination between intact and processed poly(A) tails, facilitating detailed analysis of poly(A) metabolism. It also enhances the visualization capabilities, highlighting the dynamic population of poly(A) (on plots showing mRNA-1273 shown in green), and, hence, changes in the poly(A) length. Simultaneously, the comparative abundance of each group can be assessed, providing further insight into poly(A) processing dynamics. Moreover, the distribution of intact poly(A) lengths (in blue on plots showing mRNA-1273 shown) may be used as a control and a measure of poly(A) tail length estimation accuracy, giving its consistency across analyzed conditions (please consult main figures: 1c, 1e, 2a, 2b, 2c, 4a, 4b, 4c, 4e, 5a, 5b, 5c, 5d, 5e). Of note, combined visualization of these two poly(A) populations makes analysis of the poly(A) dynamics less straightforward (Supplementary Fig. 1b, a reiteration of main figure 2b, but without the intact vs. processed poly(A) discrimination). Notably, this observation may also apply to results obtained for BNT162b2, which lacks such a feature at its 3' end. Considering the possibility of a high proportion of unprocessed poly(A) tails in BNT162b2 samples, the chance to observe the dynamic poly(A) population may be limited. Nevertheless, as demonstrated in Supplementary Fig. 1b, in case of significant re-adenylation, it can be observed regardless of the visualization method used.

Supplementary Note 3 Analysis of composite poly(A) tail from BNT162b2 vaccine with Nanopore Direct RNA Sequencing

mRNA-1273 and BNT162b2, although they target the same antigen, the SARS-CoV-2 coronavirus, and encode the same antigen, they differ in their cap structures, UTR sequences, and 3' tails[13]. The BNT162b2 vaccine has a composite poly(A) tail of 30As followed by 10 other nucleotides (GCAmΨAmΨGACmΨ), and then 70 additional As[14] (Extended Data Fig. 1c), which makes its analysis using DRS challenging, as standard nanopolish-polya algorithm fails to segment the raw current signal correctly, rarely outputting the full poly(A) tail but rather its final, 70A part. The same problem arises from the analysis of cDNA-originating reads with another algorithm implemented in dorado, where only the last, 70A-long segment is reported (Extended Data Fig. 1g, right panel, and bottom panels of Fig. 5b and Extended Data Fig. 1l).

For the analysis of BNT162b2-originating reads from DRS the nanopolish polya algorithm was modified (analogously as in the case of mRNA-1273 mΨCmΨAG) to (1) include unmapped reads enabling analysis of poly(A) lengths for sDTW-identified reads (2) detect two poly(A) segments interleaved with 10nt linker and report their lengths in the output.

The original segmentation algorithm was modified to include additional segments between adaptor and transcript (Supplementary Fig. 1c). During the analysis of the raw current signal, the standard poly(A) signal is expected after the adaptor, followed by the segment with the emissions matching the transcript segment (to catch the 10nt linker), another poly(A) signal, and finally, the transcript (as in the original algorithm). As a result, additional columns are added to the nanopolish-polya output, containing localization of each of poly(A) segments in the raw signal, their lengths, and summed poly(A) length. The results of the segmentation of a crude BNT162b2 sample are shown on Extended Data Fig. 1d. For DRS samples containing BNT162b2, the segmentation was run using the nanopolish polya-pfizer function. The source code of the modified nanopolish is available at https://github.com/LRB-IIMCB/nanopolish_mRNA-1273_BNT162b2.

Supplementary Note 4 Cytotoxicity of mRNA vaccines

To determine the optimal amount of vaccine RNA that is non-toxic to cells and, at the same time allows for efficient protein production, we distributed mRNA-1273 or BNT162b2 to HEK293T, A549 or mBMDMs in amounts ranging from 0.2µg to 10µg per well of 6-well plates. and performed the crystal violet (Sigma, C3886) staining described by Xin Chen Lab (UCSF). Briefly, cell medium was aspirated (or harvested for subsequent ELISAs if necessary), and cells were washed with 1 ml PBS. A staining solution (0.25% crystal violet in 20% methanol) was then added to each well, and cells were incubated for 10 minutes at room temperature. The staining solution was then removed and the cells were rinsed 6 times with 2 ml PBS, after which the remaining solution was evaporated, and the plates were scanned. The amount in which antigen protein was produced at an easily detectable level without severe toxic effects on cell growth in HEK293T or A549 cells (Supplementary Fig. 2a) and mBMDMs (Supplementary Fig. 2b,c) was determined to be 1µg per well.

Supplementary Note 5 Flow cytometry analysis of muscle-infiltrating immune cells following mice vaccination with mRNA-1273

Selection of muscle regions for sort experiments

For all the experiments requiring immunization of mice, we always aimed for anterior thigh muscles. However, interestingly, when isolating tissues, we observed spread/scattered hematomas (Supplementary Fig. 3a), possibly originating from tissue damage during injection, in both anterior and posterior thigh muscle regions. As this suggested that vaccine LNPs may be present in both muscles, we sorted cells from joint samples of said regions (Supplementary Fig. 3b). Later, we found that regardless of the hematomas' presence or appearance, $CD45^+$ cells infiltrated both anterior and posterior muscle regions when vaccinating mice in the anterior thigh muscles (Supplementary Fig. 3c,d). However, cells infiltrating the anterior muscle group were of different immunophenotypes (mostly $CD11C^+$, high prevalence of cells expressing both F4/80&CD64). At the same time, they were less abundant than those found in the posterior thigh muscle (mostly $CD11C^-$, low abundance of cells expressing both F4/80&CD64). We also observed that in the case of vaccinating anterior thigh muscles, cells sorted from this region had, on average, 27-fold more vaccine than those from the posterior thigh (not shown). Such analysis may lead to significantly biased results when dissociating and analyzing posterior and anterior muscles together, especially when sorting low vaccine content cells from the posterior muscle as DCs (this muscle group has a clearly separating population of $MHCII^{hi}$, $CD11C^{hi}$ as opposed to anterior muscles). In contrast, high vaccine content cells were most likely originating from anterior (vaccinated) muscle group which has much more cells positive for both F4/80 and CD64.

Thus, we decided to proceed with the vaccinated muscle only for further experiments. We have performed time course experiments (6 h, 12 h, 24 h and 48 h post-administration) to gain insight into both immune cells and vaccine mRNA dynamics (Supplementary Fig. 4a, b).

Looking at the general cell abundance upon vaccination, we observed substantial mice-to-mice variability, with no clear trend between time points (Supplementary Fig. 4b). However, the upper limit of recovered live cells was never more than 1.2×10^6. In the case of unvaccinated muscles or muscles vaccinated with 0.9% NaCl we observed very low numbers of live cells ($<2\times10^5$) in which $CD45^+$ accounted for only ~20% (Supplementary Fig. 4c).

The amount of CD45+ cells among live cells is more or less static between time points. However, the immunophenotypes were dynamic. The first change we spotted in time course experiments was the behavior of CD11c for which the number of positive events in CD45 population increased with time. The majority of muscle infiltrating $CD45^+$ cells are also positive for both F4/80 and CD64, indicating that macrophage-like cells are mostly recruited to the injection site. 24h post-vaccination, the majority

of cells display high levels of $CD11C^+$, which implies that infiltrating cells, both macrophages and DCs, gain adhesion to ECM over time after infiltration (the presence of CDC11c on macrophage and monocyte type cells is well known phenomenon[15]).

Looking at cells expressing both CD64 and F4/80, we can see that the geometric Mean Fluorescence Intensity (gMFI) for CD11C and MHCII increases over time, suggesting higher expression per cell in consecutive time points. Populations identified as DCs display orders of magnitude higher expression of both markers at 6h post-vaccination (Supplementary Fig. 4a).

Since we observed obvious time-dependent expression of CD11C in $CD45^+$ cells infiltrating the muscles post-injection, we have analyzed the mRNA-1273 abundance in CD11C- and $CD11C^+$ populations of $CD45^+$, $F4/80^{+,}$ $CD64^+$, $MHCII^{-/low}$ cells (considered as macrophages). Notably, $CD11C^+$ cells had, on average, more vaccine than $CD11C^-$ (Supplementary Fig. 4d).

All these findings have some implications for the sorting strategy. Often CD11c and MHCII are primary markers that distinguish between DCs and Macrophages. However, after vaccine administration, the expression of both markers is rising over time. The trajectory suggests that macrophages overlap with $MHCII^+$ and $CD11C^+$ events, especially at later time points. Additionally, the events usually assumed as macrophages ($CD11C^-$; $F4/80^+$&$CD64^+$) are likely less adhesive macrophages/monocytes with lower uptake potential than $CD11C^+$; $F4/80^+$&$CD64^+$. Finally, the majority of $CD45^+$ cells infiltrating the muscle are $MHCII^{-/low}$; $F4/80^+$&$CD64^+$, indicating that macrophage-like cells dominate the cellular landscape of muscle infiltrating cells. To better discriminate between those cell types, we utilised the gating strategy (Supplementary Fig. 5) for acquiring DCs and M0s by FACS in which:

1. DCs are MHCII+/high, CD11C+ sorted from CD45+ cells with the additional exclusion of cells double positive for F4/80 and CD64 markers.
2. Macrophages are MHCII-/low, CD11C+, F4/80+, CD64+ sorted from CD45+ cells.

Analysis of dynamics of muscle-resident immune cells following intramuscular immunization with mRNA-1273 is presented on Extended Data Fig. 3c and Supplementary Fig. 6.

Supplementary Note 6 Design of reporter mRNAs encoding OVA, PfCSP and ZikVE

A series of reporter mRNAs was generated to investigate the potential roles of the capping technique, mRNA-1273/BNT162b2 5' and 3'UTRs and poly(A) tail composition (Extended Data Fig. 8a) on susceptibility to re-adenylation by TENT5A. 5' and 3'UTR sequences are the same as described previously[16]. CDS sequences were derived from full-length sequences of:

- Plasmodium falciparum circumsporozoite (CS) protein (PfCSP_3D7; NCBI reference sequence: XP_001351122.1)[17],
- ovalbumin (OVA; sequence available at https://www.trilinkbiotech.com/)
- Zika virus protein E (ZikVE; NCBI reference sequence: YP_002790881.1)[18].

Sequences were optimized for expression in mouse cells using software available at benchling.com. It should be noted that for PfCSP the optimalization was challenging and possibly leading to suboptimal expression due to multiple repeated sequences (Extended Data Fig. 7h).

To get better control on poly(A) lengths of produced reporter RNAs, we decided to produce DNA templates for IVT reactions with PCR using primers complementary to UTRs (Extended Data Fig. 8b). On the primer matching the 5'UTR the RNA T7 promoter Φ6.5 (TAATACGACTCACTATAGGG) was introduced on the overhang. On the primer matching the 3'UTR the poly(A) tail from either mRNA-1273 or Pfizer BNT162b2 and either with or without pentamer was introduced on the overhang. PCR products were verified through agarose gel electrophoresis and purified with KAPA Pure Beads (KAPA Biosystems).

The *in vitro* transcription was carried out with T7 RNA polymerase[20] (in-house prepared) in the presence of N^1meΨTP (Advent Bio). For the enzymatic capping synthesized RNAs were provided with Cap-1 using Vaccinia Capping System (homemade) and Cap 2′-O-Methyltransferase (NEB) according to the manufacturer's protocol with the replacement of the manufacturer's Vaccinia Capping Enzyme with the in-house prepared one[21]. The purity and integrity of the mRNA preparations were determined by the automated electrophoresis (TapeStation 2200, Agilent Technologies, Extended Data Fig. 8c,d), indicating good quality and reproducibility of the synthesis.

Supplementary Note 7 Comparison of mRNA-1273 and BNT162b2 vaccines and mRNA reporters used in this work

The unexpected observation of limited re-adenylation of BNT162b2 prompted us to look for factors that might distinguish the two vaccines and influence their metabolism. Even if both mRNAs encode the same protein, their sequences were optimized through different approaches, leading to notable variations in GC content, codon usage, cap types, poly(A) composition, and secondary structure, as previously reported[13].

BNT162b2 vaccine is capped co-transcriptionally with a trinucleotide Cap1 analog (CleanCap® AG featuring 3' O-methylation on m7G, $(m_2^{7,3'-O})$Gppp$(m^{2'-O})$ApG, Extended Data Fig. 7a), whereas mRNA-1273 is capped posttranscriptionally to generate natural Cap1 by vaccinia virus capping enzyme (VCE) and mRNA Cap 2′-O-Methyltransferase[22] (Extended Data Fig. 7b). Chemically, the only difference between both is the 3' O-methylation on m7G in the cap used for BNT16b2 manufacturing, and although the manufacturer claims it provides better translation efficiency, it is unclear how this can influence the susceptibility to re-adenylation by TENT5s.

Another important difference in both vaccine designs lies in the UTR sequences. BNT162b2 5' UTR is derived from the human α-globin (*HBA1*), whereas the source of 5'UTR of mRNA-1273 is unclear[16]. For the 3′ UTR, the BNT162b2 has 2-segment UTR composed of human mitochondrial 12S rRNA (*mtRNR1*) fragment and the other from human mRNA AES/TLE5 gene[13,16], while in the mRNA-1273 shorter 3'UTR, originating from human α-globin (*HBA1*) gene, was used. The influence of both UTRs designs on translation was analyzed elsewhere[16], indicating similar translation efficiencies, despite different secondary structures.

Both mRNA-1273 and BNT162b2 sequences have high GC content compared to endogenous mammalian transcripts. Moreover, it is strikingly high (~60%) compared to the reference spike protein sequence (~40%), but it is the result of elimination of most of the uridines in 3rd codon position, to limit the interferon response.

We also performed an in-depth analysis of codon usage based on several metrics, such as Effective Number of Codons (ENC), Codon Adaptation Index (CAI) and Frequency of Optimal Codons (FOP). The ENC indicates the extent to which codon usage deviates from a uniform distribution. Lower ENC values reflect a preference towards specific codons, while higher values suggest a more balanced utilization of synonymous codons. Both mRNA-1273 (22.79) and BNT162b2 (29.52) exhibit a strong bias toward certain codons (Extended Data Fig. 7c) compared to the original spike protein sequence (44.3), murine protein-coding transcripts (51.72), and IVT-transcribed reporter sequences coding for ovalbumin as well as antigens from Zika virus and *Plasmodium falpicarum* described in this study (60.06).

The remaining indices, CAI and FOP rely on the comparison to the codon frequency in a reference set of genes, in our case: 500 highly expressed genes in mBMDMs, according to one of our previous work[23]. The CAI measures how closely a gene's codon usage aligns with optimal codons. Higher CAI values signify better adaptation, suggesting enhanced translation efficiency and potentially greater protein expression. Both mRNA-1273 and BNT162b2 exhibit CAI scores approaching 1 (0.98 and 0.93, respectively, Extended Data Fig. 7d), indicating nearly ideal adaptation. These values are consistent with those reported previously. In contrast, the coronaviral spike protein scored significantly lower (0.63) than typical values for host transcripts and reporter sequences (both approximately 0.75). The two vaccines also differ in their FOP values (Extended Data Fig. 7e), with mRNA-1273 scoring 0.94 and BNT162b2 scoring 0.79. The FOP is a simplified measure of codon usage bias, providing a lower-resolution assessment compared to CAI. We also performed a sliding-window analysis of codon usage to test whether vaccine sequences exhibit distinct codon usage patterns, again revealing that mRNA-1273 is more optimized compared to BNT162b2 and reporters described in this study (Extended Data Fig. 7f). Finally, we also compared the frequency of individual codons in the two vaccines, which proved subtle differences (e.g. ACA, CCT, TCT, GAA, AGA, TCC almost absent in mRNA-1273, unlike BNT162b2, Extended Data Fig. 7g). More statistical properties of coding sequences used in this work are provided in the Supplementary Table 14.

Overall, all metrics applied suggest that both vaccines are highly optimized, with mRNA-1273 exhibiting even greater optimization.

Supplementary References

1. Sahin, U., Karikó, K. & Türeci, Ö. mRNA-based therapeutics — developing a new class of drugs. *Nat. Rev. Drug Discov.* **13**, 759–780 (2014).
2. Karikó, K. *et al.* Incorporation of Pseudouridine Into mRNA Yields Superior Nonimmunogenic Vector With Increased Translational Capacity and Biological Stability. *Mol. Ther.* **16**, 1833–1840 (2008).
3. Svitkin, Y. V. *et al.* N1-methyl-pseudouridine in mRNA enhances translation through eIF2α-dependent and independent mechanisms by increasing ribosome density. *Nucleic Acids Res.* **45**, 6023–6036 (2017).
4. Anderson, B. R. *et al.* Incorporation of pseudouridine into mRNA enhances translation by diminishing PKR activation. *Nucleic Acids Res.* **38**, 5884–5892 (2010).
5. Andries, O. *et al.* N1-methylpseudouridine-incorporated mRNA outperforms pseudouridine-incorporated mRNA by providing enhanced protein expression and reduced immunogenicity in mammalian cell lines and mice. *J. Controlled Release* **217**, 337–344 (2015).
6. Gebre, M. S. *et al.* Optimization of non-coding regions for a non-modified mRNA COVID-19 vaccine. *Nature* **601**, 410–414 (2022).
7. Lutz, J. *et al.* Unmodified mRNA in LNPs constitutes a competitive technology for prophylactic vaccines. *Npj Vaccines* **2**, 1–9 (2017).
8. Garalde, D. R. *et al.* Highly parallel direct RNA sequencing on an array of nanopores. *Nat. Methods* **15**, 201–206 (2018).
9. Fleming, A. M. & Burrows, C. J. Nanopore sequencing for N1-methylpseudouridine in RNA reveals sequence-dependent discrimination of the modified nucleotide triphosphate during transcription. *Nucleic Acids Res.* **51**, 1914–1926 (2023).

10. Kovaka, S., Fan, Y., Ni, B., Timp, W. & Schatz, M. C. Targeted nanopore sequencing by real-time mapping of raw electrical signal with UNCALLED. *Nat. Biotechnol.* **39**, 431–441 (2021).
11. Meert, W., Hendrickx, K., van Craenendonck, T. & Robberechts, P. DTAIDistance (Version v2.3.5). Zenodo https://doi.org/10.5281/zenodo.5901139 (2022).
12. Workman, R. E. *et al.* Nanopore native RNA sequencing of a human poly(A) transcriptome. *Nat. Methods* 1–9 (2019) doi:10.1038/s41592-019-0617-2.
13. Xia, X. Detailed Dissection and Critical Evaluation of the Pfizer/BioNTech and Moderna mRNA Vaccines. *Vaccines* **9**, (2021).
14. Vogel, A. B. *et al.* BNT162b vaccines protect rhesus macaques from SARS-CoV-2. *Nature* **592**, 283–289 (2021).
15. Sándor, N. *et al.* CD11c/CD18 Dominates Adhesion of Human Monocytes, Macrophages and Dendritic Cells over CD11b/CD18. *PLOS ONE* **11**, e0163120 (2016).
16. Zhang, L. *et al.* Effect of mRNA-LNP components of two globally-marketed COVID-19 vaccines on efficacy and stability. *Npj Vaccines* **8**, 1–14 (2023).
17. Hayashi, C. T. H. *et al.* mRNA-LNP expressing PfCSP and Pfs25 vaccine candidates targeting infection and transmission of Plasmodium falciparum. *Npj Vaccines* **7**, 1–14 (2022).
18. Essink, B. *et al.* The safety and immunogenicity of two Zika virus mRNA vaccine candidates in healthy flavivirus baseline seropositive and seronegative adults: the results of two randomised, placebo-controlled, dose-ranging, phase 1 clinical trials. *Lancet Infect. Dis.* **23**, 621–633 (2023).
19. Sikorski, P. J. *et al.* The identity and methylation status of the first transcribed nucleotide in eukaryotic mRNA 5′ cap modulates protein expression in living cells. *Nucleic Acids Res.* **48**, 1607–1626 (2020).

20. Dousis, A., Ravichandran, K., Hobert, E. M., Moore, M. J. & Rabideau, A. E. An engineered T7 RNA polymerase that produces mRNA free of immunostimulatory byproducts. *Nat. Biotechnol.* **41**, 560–568 (2023).

21. Fuchs, A.-L., Neu, A. & Sprangers, R. A general method for rapid and cost-efficient large-scale production of 5′ capped RNA. (2016) doi:10.1261/rna.056614.116.

22. Corbett, K. S. *et al.* SARS-CoV-2 mRNA Vaccine Design Enabled by Prototype Pathogen Preparedness. *Nature* **586**, 567–571 (2020).

23. Liudkovska, V. *et al.* TENT5 cytoplasmic noncanonical poly(A) polymerases regulate the innate immune response in animals. *Sci. Adv.* **8**, eadd9468 (2022).

Supplementary Tables description

Supplementary Table 1

Mean poly(A) lengths for genes expressed in WT and *Tent5a*$^{Flox/Flox}$/*Tent5c*$^{-/-}$ mBMDMs. Statistics for change in poly(A) lengths were calculated using the Kruskall-Wallis test for WT samples and genes with at least 20 reads in each group. Multiple testing correction (adjusted P value) was done using Benjamini-Hochberg's method. Effect size is calculated as rank epsilon squared. For transcripts with the most pronounced poly(A) change (adjusted P value < 0.001) their cluster membership is indicated.

Supplementary Table 2

GO Terms (Biological Process) enriched genes in clusters exhibiting clear patterns of poly(A) length change in WT mBMDMs upon mRNA-1273 delivery.

Supplementary Table 3

Mean poly(A) lengths for genes expressed in hMDMs. Statistics for change in poly(A) lengths were calculated using the Kruskall-Wallis test for WT samples and genes with at least 20 reads in each group. Multiple testing correction (adjusted P value) was done using the Benjamini-Hochberg method. Effect size is calculated as rank epsilon squared. For transcripts with the most pronounced poly(A) change (adjusted P value < 0.001) their cluster membership is indicated.

Supplementary Table 4

Differential expression of genes at mRNA-1273 injection sites after mice immunization. DESeq2 LRT test statistics and clustering of significantly changed genes are shown.

Supplementary Table 5

GO Terms (Biological Process) enriched genes in clusters exhibiting clear patterns of expression change at mRNA-1273 injection sites after mice immunization.

Supplementary Table 6

Differential expression of genes in WT BMDMs upon mRNA-1273 delivery. DESeq2 LRT test statistics and clustering of significantly changed genes shown.

Supplementary Table 7

Differential expression of genes in hMDMs upon mRNA-1273 delivery. DESeq2 LRT test statistics and clustering of significantly changed genes are shown. In separate sheets differential expression statistics at individual timepoints (compared to time 0 h) are provided.

Supplementary Table 8

GO Terms (Biological Process) enriched for genes in the individual clusters (please refer to Extended Data Fig. 4c) or genes upregulated in WT BMDMs upon mRNA-1273 delivery (results for individual timepoints are provided in separate sheets).

Supplementary Table 9

GO Terms (Biological Process) enriched for genes upregulated in hMDMs upon mRNA-1273 delivery. In separate sheets results for individual timepoints are provided.

Supplementary Table 10

Differential expression of genes in *Tent5a*$^{Flox/Flox}$/*Tent5c*$^{-/-}$ BMDMs upon mRNA-1273 delivery. DESeq2 LRT test statistics are shown, and WT BMDMs cluster membership is indicated.

Supplementary Table 11

List of antibodies used in this work. Separate shets for antibodies used for Western Blot or cytrometric analyses.

Supplementary Table 12

List of oligonucleotides used in this work.

Supplementary Table 13

List of nanopore sequencing datasets, with their accession numbers at European Nucleotide Archive (ENA).

Supplementary Table 14

Coding sequence statistic properties of mRNA-127, BNT162b2, and reporters used in this work.

Supplementary Table 14

parameter	SARS-CoV2 spike	BNT162b2	mRNA-1273	Ova	PfCSP	ZikVE
**Coding sequence length**	3819	3819	3819	1206	1191	1548
**GC Content**	0.37	0.57	0.62	0.5	0.48	0.54
**Codon Adaptation Index**	0.63	0.93	0.98	0.75	0.78	0.75
**Relative Synonymous Codon Usage**	1.35	2.34	3.08	1.13	1.1	1.12
**Effective Number of Codons**	44.3	29.52	23.68	61	61	58.19
**Relative Codon Bias Strength**	0.14	0.6	0.56	0.16	0.42	0.14
**Directional Codon Bias Score**	1.42	2.19	2.06	1.55	2.11	1.49
**Codon Deviation Coefficient**	0.07	0.21	0.2	0.1	0.33	0.08
**Measure Independent of Length and Composition**	-0.33	0.1	0.4	-0.52	-0.54	-0.49
**Intrinsic Codon Deviation Index**	0.17	0.47	0.86	0.06	0.07	0.07
**Synonymous Codon Usage Order**	0.16	0.49	0.79	0.06	0.05	0.07
**Weighted Sum of Relative Entropy**	0.82	0.49	0.19	0.88	0.93	0.88
**Codon Preference**	1.05	1.48	1.42	1.06	1.05	1.06
**Maximum-likelihood Codon Bias**	0.72	2.68	4.1	0.28	0.16	0.29
**Codon Frequency Distribution**	0.05	0	0	0.04	0.03	0.04
**Frequency of Optimal Codons**	0.25	0.79	0.94	0.55	0.55	0.56
**Codon Usage Similarity Index**	-1.26	3.4	4.75	1.09	1.11	1.2
**Codon Bias Index**	-0.2	0.64	0.83	0.2	0.17	0.21
**Mean Dissimilarity-based Index**	0.99	0.19	0.32	0.04	0.15	0.04
**Relative Codon Adaptation**	1.05	1.22	1.19	1.12	1.03	1.1
**Codon Usage Frequency Similarity**	0.36	0.41	0.56	0.04	0.09	0.05
**Codon Usage Bias**	0.62	0.67	0.97	0.09	0.07	0.09
**tRNA Adaptation Index**	0.14	0.18	0.19	0.16	0.17	0.16
**Genetic tRNA Adaptation Index**	0.17	0.2	0.21	0.19	0.2	0.19
**P2 Index**	0.48	0.58	0.6	0.52	0.53	0.49
**GC Content at the Third Position of Synonymous Codons**	0.27	0.81	0.96	0.61	0.57	0.62
**GC Content at the First Position of Synonymous Codons**	0.45	0.5	0.51	0.53	0.48	0.55
**GC Content at the Second Position of Synonymous Codons**	0.4	0.4	0.4	0.37	0.38	0.44
**Effective Number of Codon Pairs**	36.05	26.24	21.13	28.78	61	31.31
**Codon Pair Score**	0.09	-0.07	-0.05	-0.11	-0.07	-0.04
**Codon Volatility**	0.77	0.76	0.76	0.76	0.78	0.75

Supplementary Figures

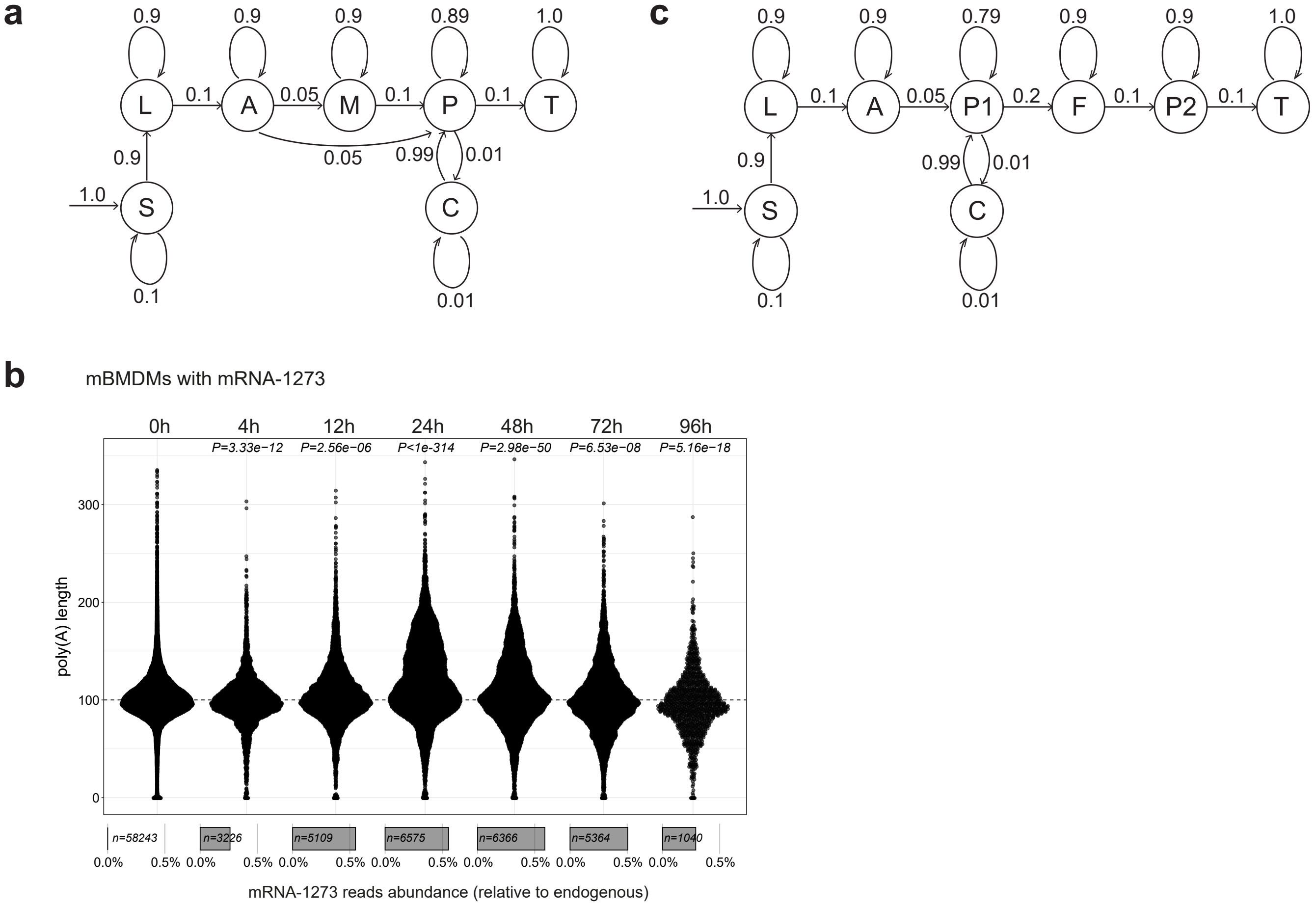

Supplementary Figure 1

a, The state transitions of the segmentation HMM implemented in nanopolish-polya for the analysis of DRS reads originating from mRNA-1273. S - start, L- leader, A - adaptor,
M - mΨCmΨAG, P - poly(A), C - cliff, T -transcript

b, mRNA-1273 poly(A) lengths distribution in mBMDMs incubated with vaccine LNP for up to 96 h. Average distributions for 3 replicates are shown, without dividing the reads into intact and processed poly(A) tails (please compare Main Figure 2e).

c, The state transitions of the segmentation HMM implemented in nanopolish-polya for the analysis of DRS reads originating from BNT162b2. S - start, L- leader, A - adaptor,
P1 - first segment of poly(A), C - cliff, F - poly(A) linker, P2 - second segment of poly(A), T -transcript

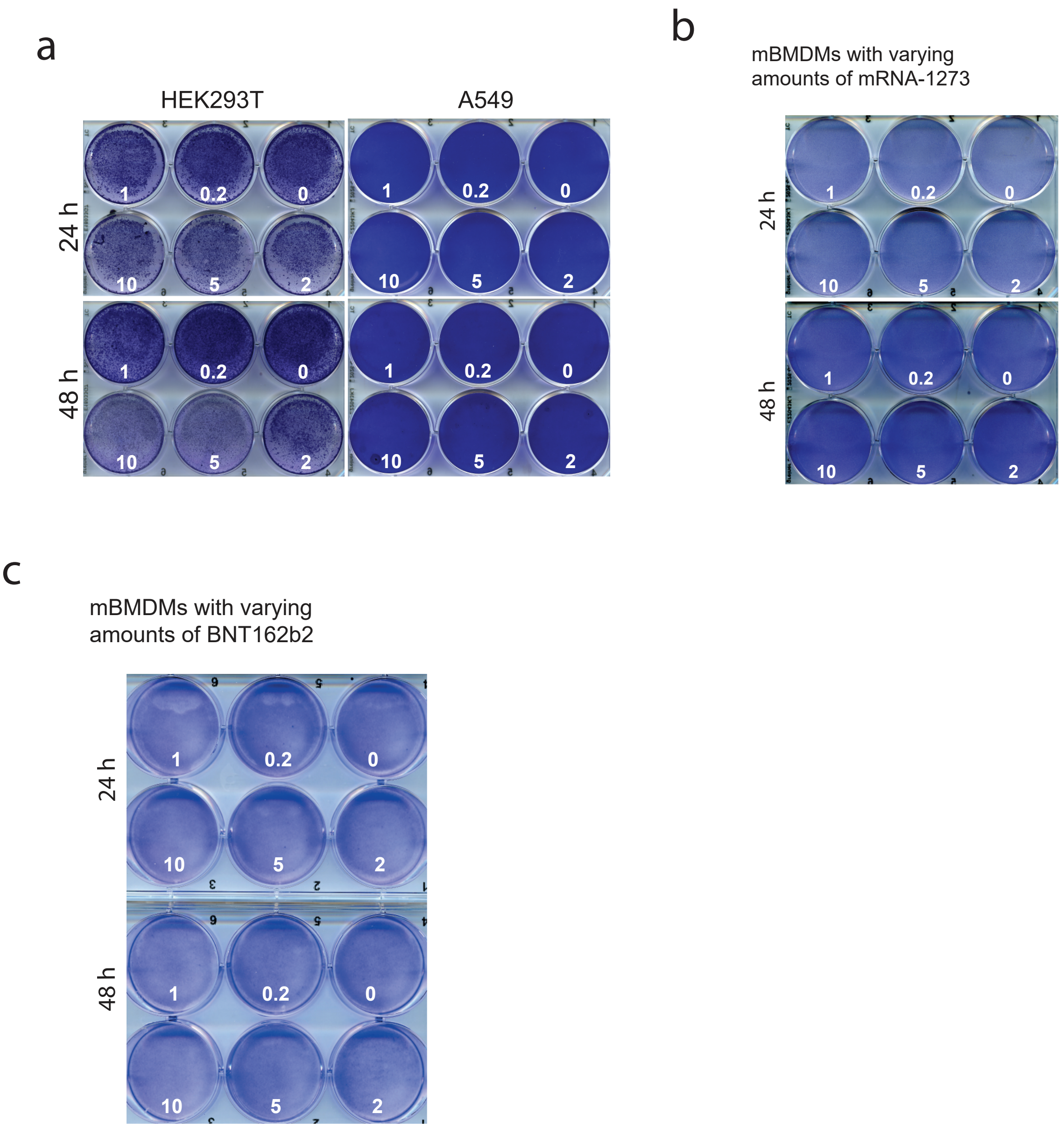

Supplementary Figure 2

a-b, Viability of **(a)** HEK293T or A549 cells, or **(b)** mBMDMs incubated with 0.2-10 µg of mRNA-1273 per well of 6-well plate for 24h or 48h, assessed with crystal violet staining.

c, Viability of mBMDMs incubated with 0.2-10 µg of BNT162b2 per well of 6-well plate for 24h or 48h, assessed with crystal violet staining.

Amounts of RNA [ug] indicated for each well (white font numbers).

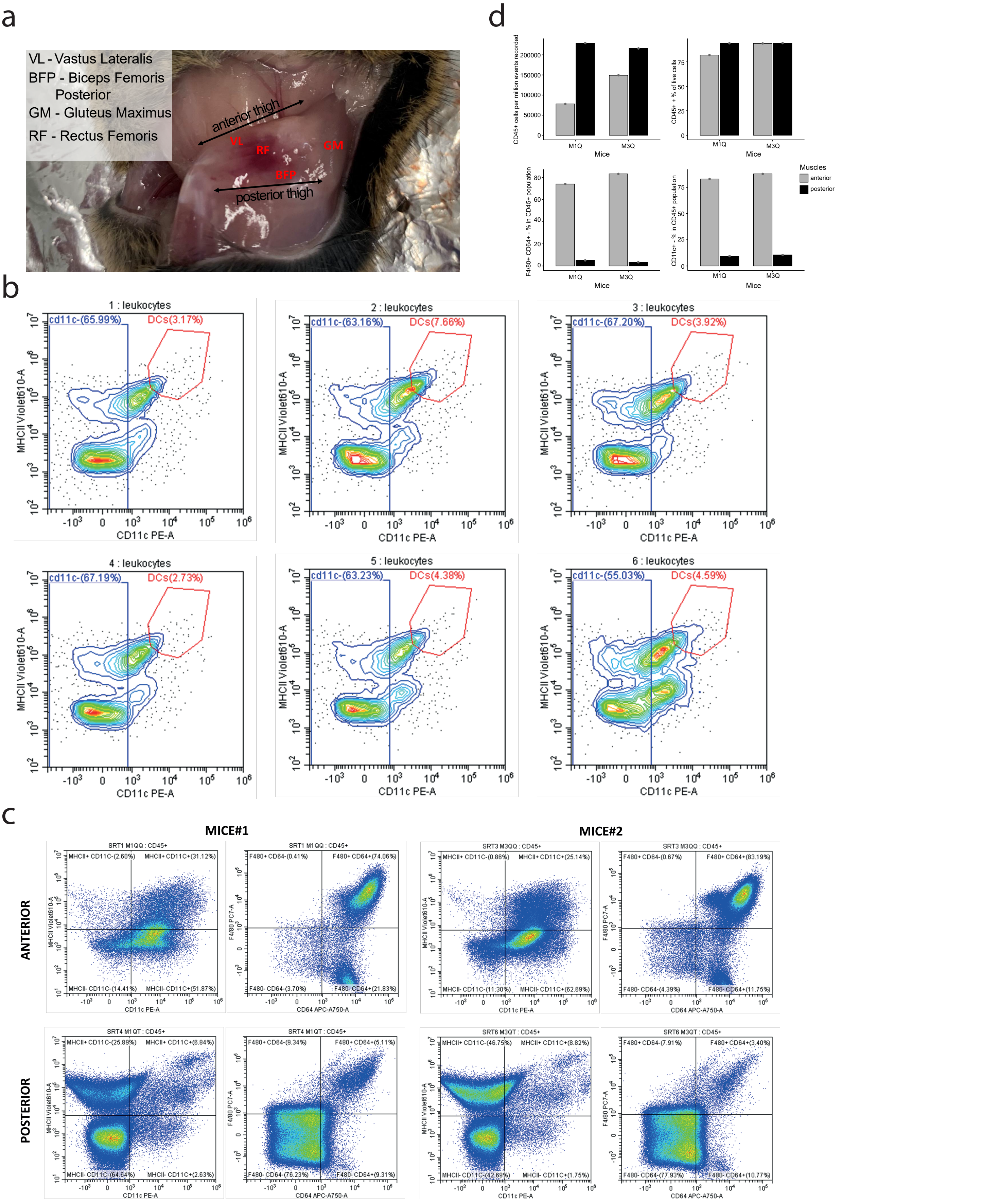

Supplementary Figure 3

a, Representative picture showing hematoma in the muscle following mice immunization.

b, Mixed anterior and posterior muscle samples – immunophenotypes of CD45+ cells with respect to MHCII and CD11C, 24h post-vaccination with Moderna

c, Immunophenotypes of CD45+ cells with respect to MHCII, CD11C, CD64 and F4/80, comparison of anterior and posterior thigh muscle regions. Here both mice were vaccinated in the anterior thigh muscle region with Moderna vaccine. Analysed 24h post-vaccination.

d, Flow cytometry statistics of cells isolated from anterior or posterior regions of thigh isolated from mice vaccinated to anterior region of the thigh, 24h post vaccination with Moderna. Quantification of CD45+ cells infiltrating muscles (top left). Percent of CD45+ in live cells (top right). Percent of F4/80+, CD64+ cells in CD45+ (bottom left). Percent of CD11C+ cells in CD45+ population (bottom right).

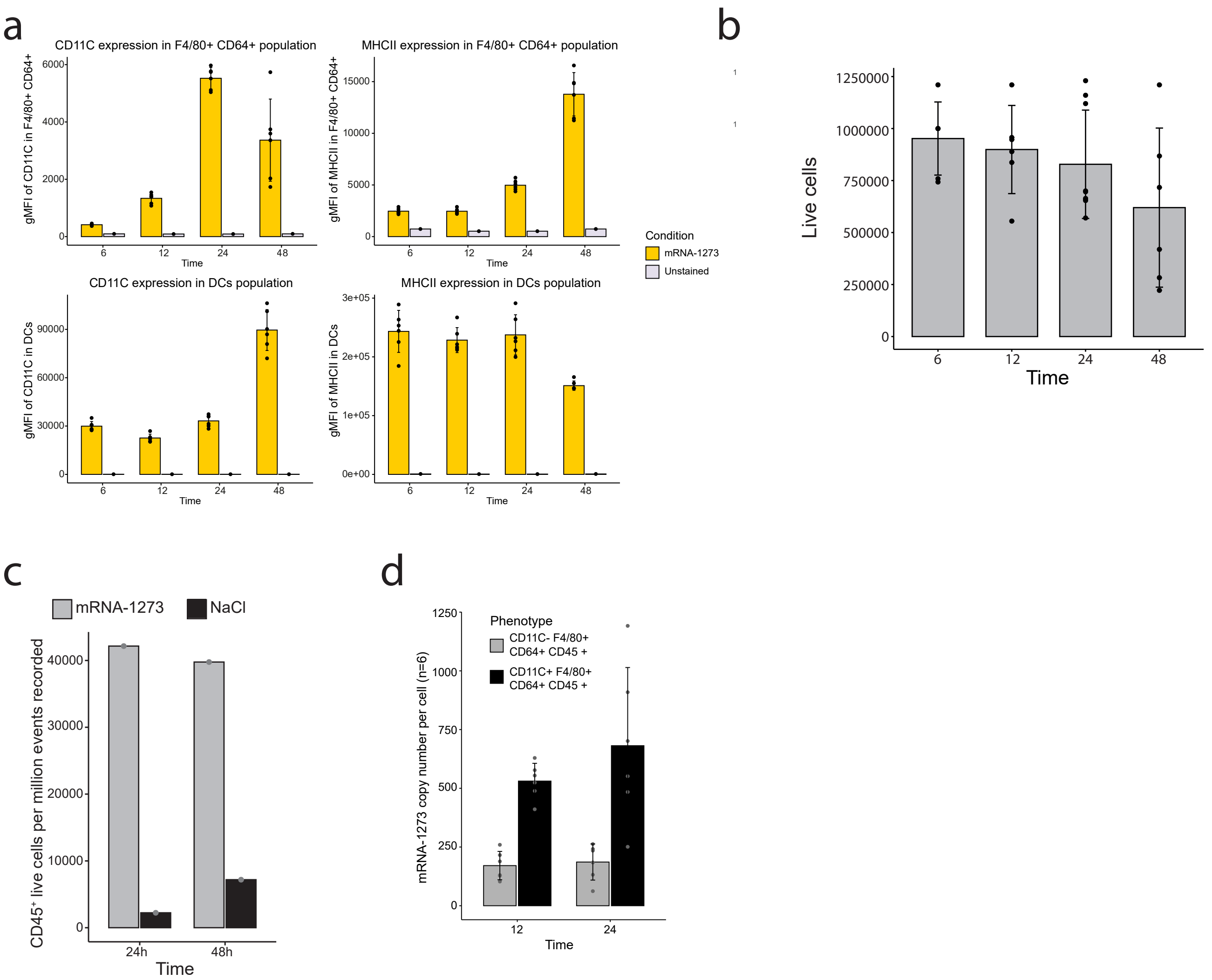

Supplementary Figure 4

a, Geometric Mean Fluorescence Intensity (gMFI) calculated for CD11C and MHCII in selected populations of cells infiltrating the anterior thigh muscles at given time after IM injection (n= 6 for each timepoint, unstained shown for reference). CD11C gMFI based expression in F4/80+ CD64+ population (top left). MHCII gMFI based expression in F4/80+ CD64+ population (top right). CD11C gMFI based expression in DCs population (bottom left) MHCII gMFI based expression in F4/80+ CD64+ population (bottom right)

b, Cells infiltrating anterior muscles (vaccinated with Moderna), counted with countess cell counter (trypan blue corrected).

c, Abundance of live CD45+ cells following immunization with mRNA-1273 (yellow) or injection of NaCl (red)

d, RT-qPCR from sorted cells – average copy number of Moderna vaccine per cell in CD11C- or CD11C+ subpopulations of F4/80+, CD64+, CD45+, MHCII-/low cells sorted 12h or 24h post-vaccination.

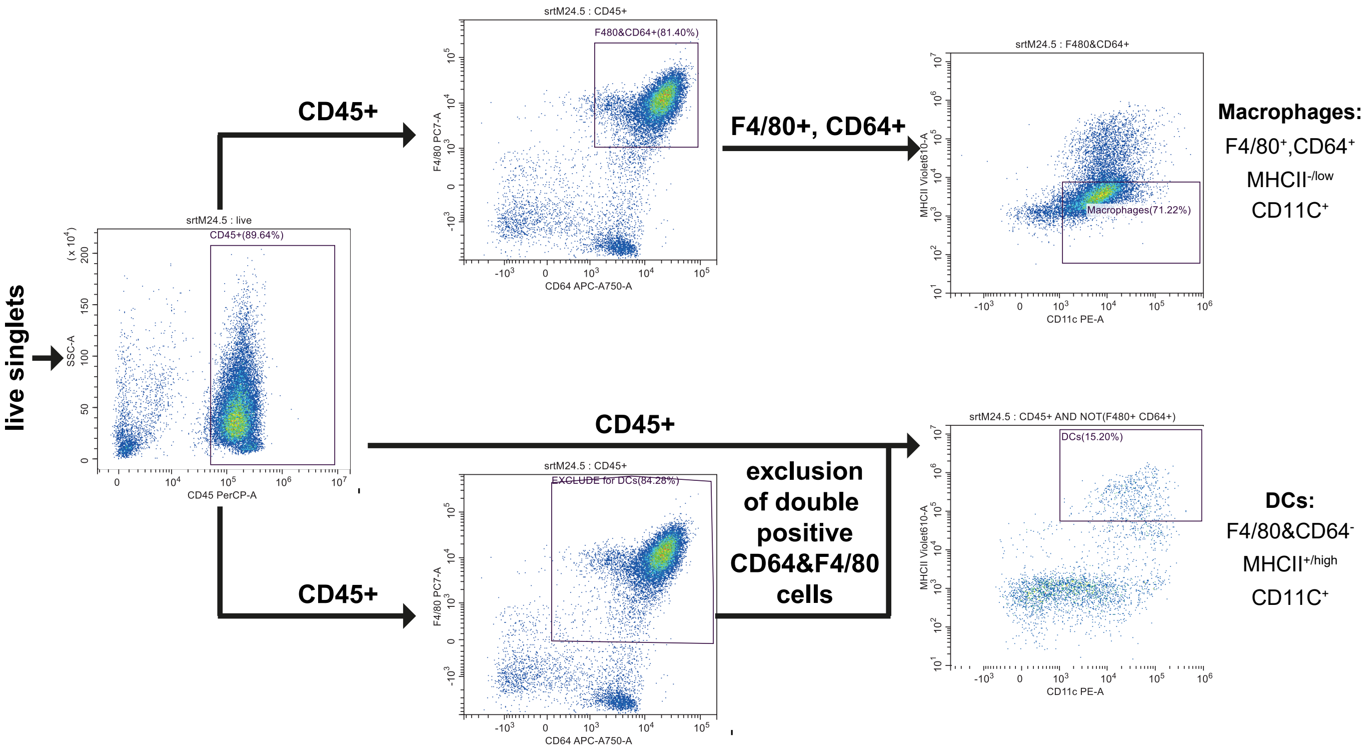

Supplementary Figure 5.

Gating strategy for sorting macrophage and DCs population from injection sites (related to Extended Data Figure 3c).

The 7-colour flow cytometry staining was used for pure sort of macrophages and dendritic cells. Cells were stained with fluorochrome-conjugated antibodies against: CD45–PerCP-Cy5.5, CD11b–BV786, F4/80–PE-Cy7, I-A/I-E (MHCII)–BV605, CD11c–PE, CD64–APC-Fire750. To exclude dead cells LIVE/DEAD™ Fixable Violet Dead Cell Stain. To sort pure macrophages and dendritic cells a restrictive gating strategy was used. Gating strategy was based on gating live singlets, next we gated leukocytes (CD45-positive cells), and cells identified as $CD11c^+$, $F4/80\&CD64^+$, I-A/I-E $(MHCII)^{-/low}$ were sorted as macrophages. $CD11c^+$ double positive for F4/80 and CD64 were excluded and remaining cells, $CD11c^+$, $F4/80\&CD64^-$, I-A/I-E $(MHCII)^+$ were sorted as dendritic cells (DCs).

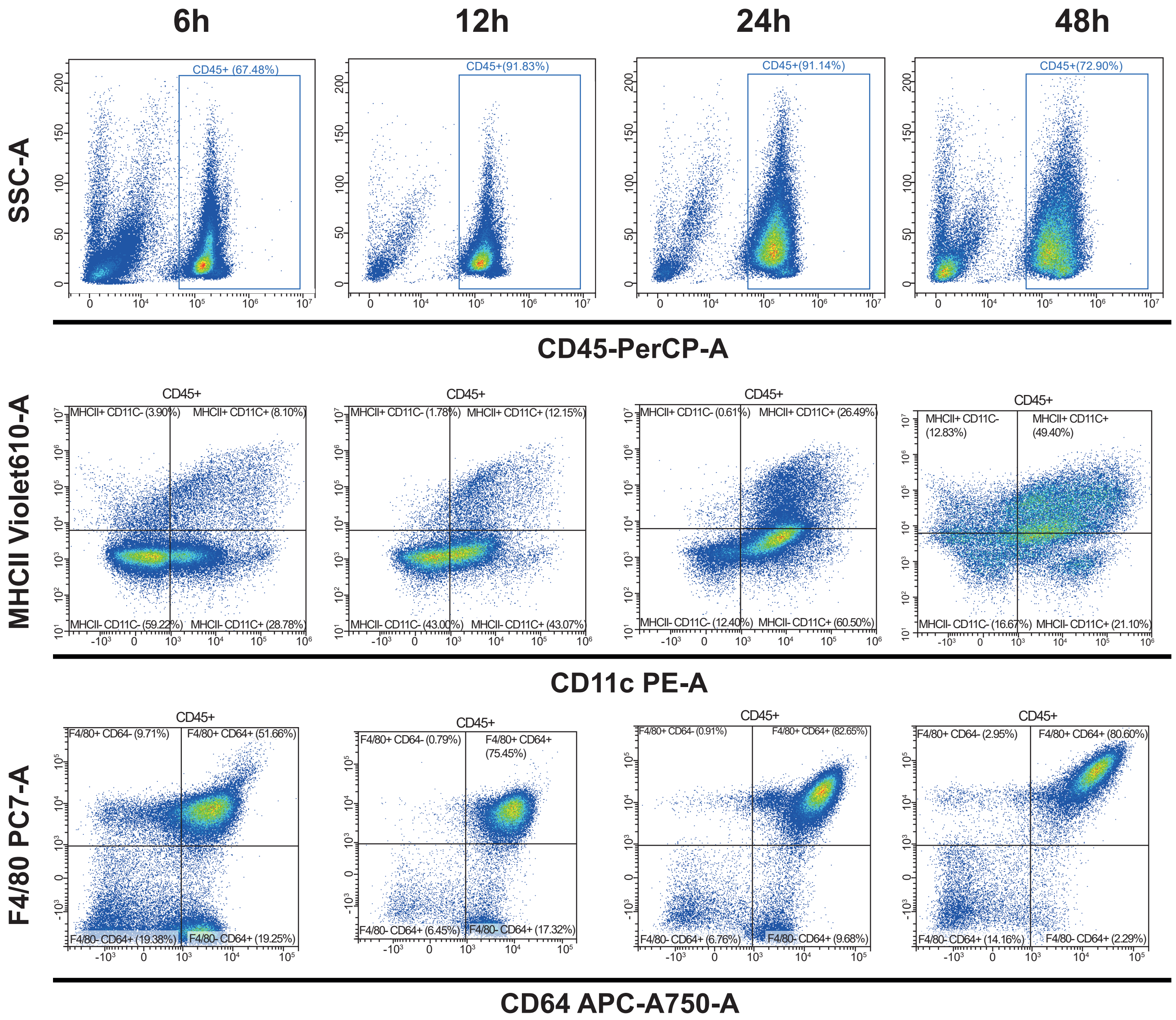

Supplementary Figure 6.

Dynamics of muscle-resident immune cells following intramuscular immunization with mRNA-1273 (related to Figure 2)

Immunophenotypes of cells isolated from muscle tissues surrounding mRNA-1273 injection site 6, 12, 24 or 48h following immunization of WT mice. Immunophenotypes for CD45 (top panel), CD11c and MHCII (middle panel), and CD64 and F4/80 (top panel) are shown.

Supplementary Information Figure 7 Source Data (scans of uncropped blots 1 of 6)

a. Scans related to Figure 1d

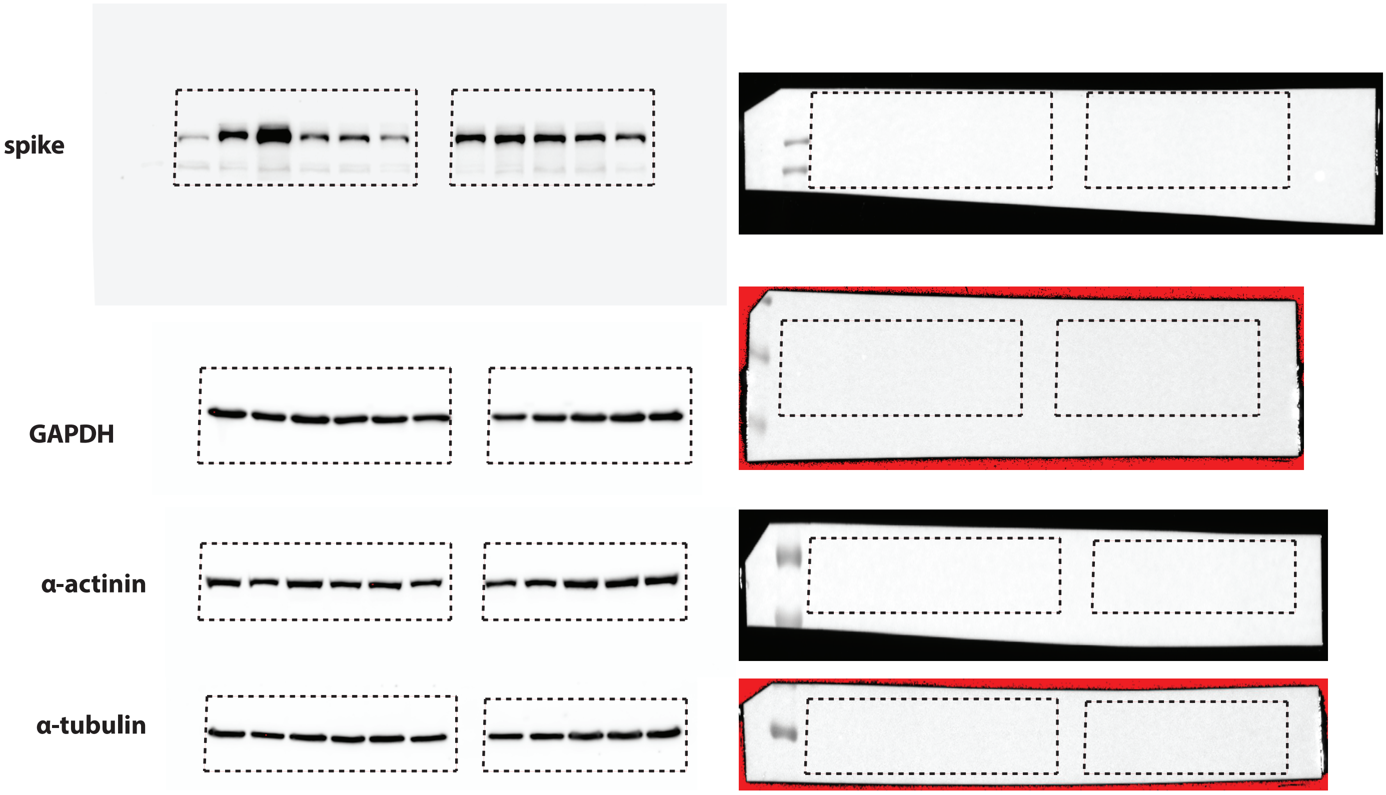

b. Scans related to Figure 3e

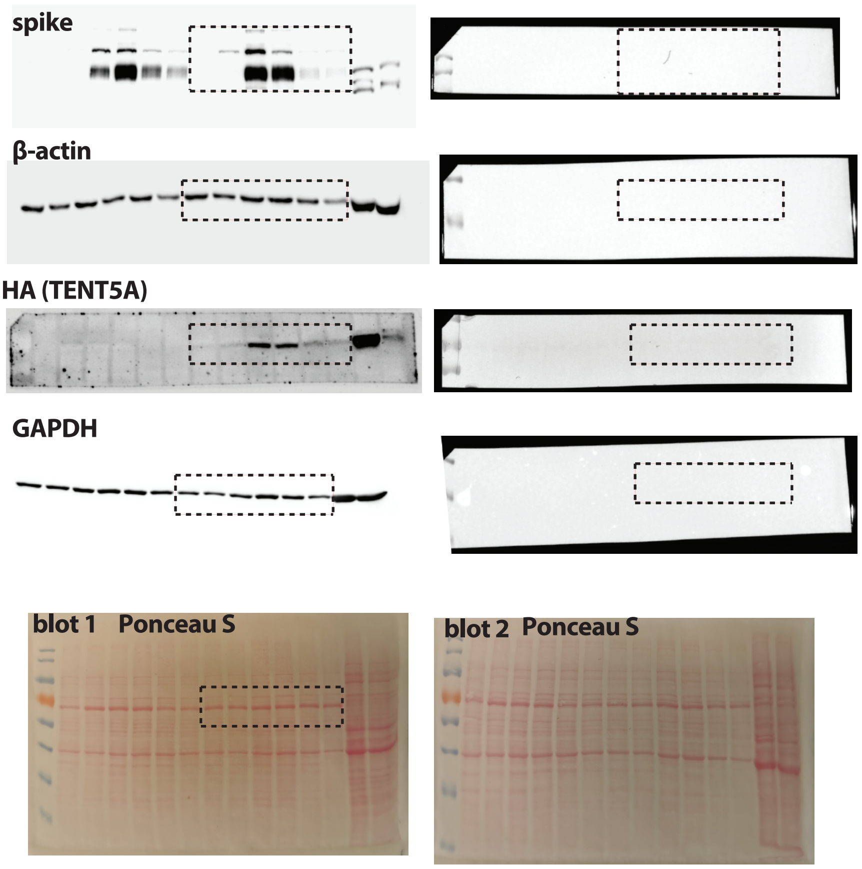

Supplementary Information Figure 7 Source Data (scans of uncropped blots 2 of 6)

c. Scans related to Extended Data Figure 1i

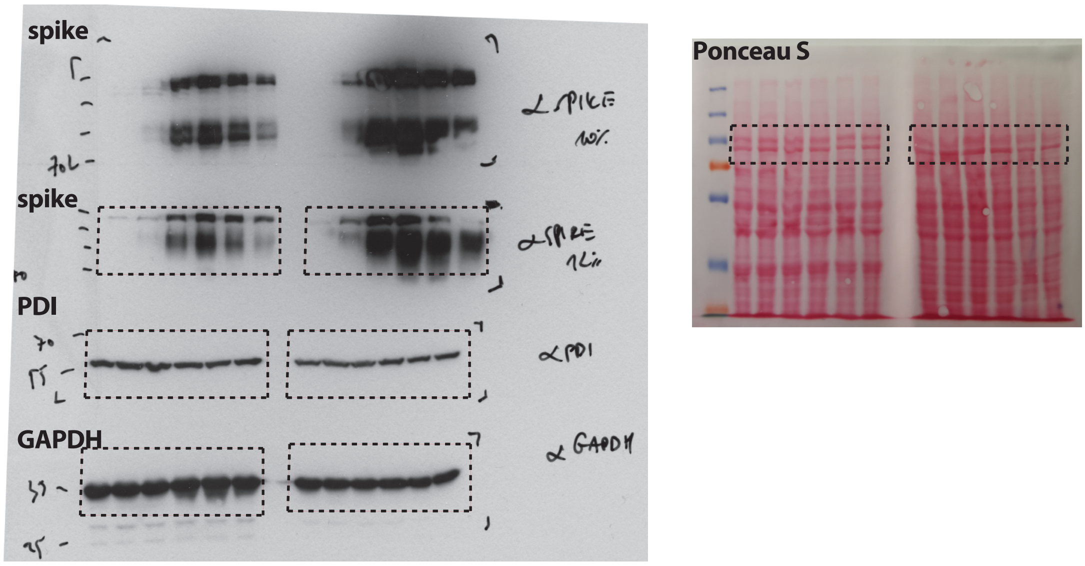

d. Scans related to Extended Data Figure 1k

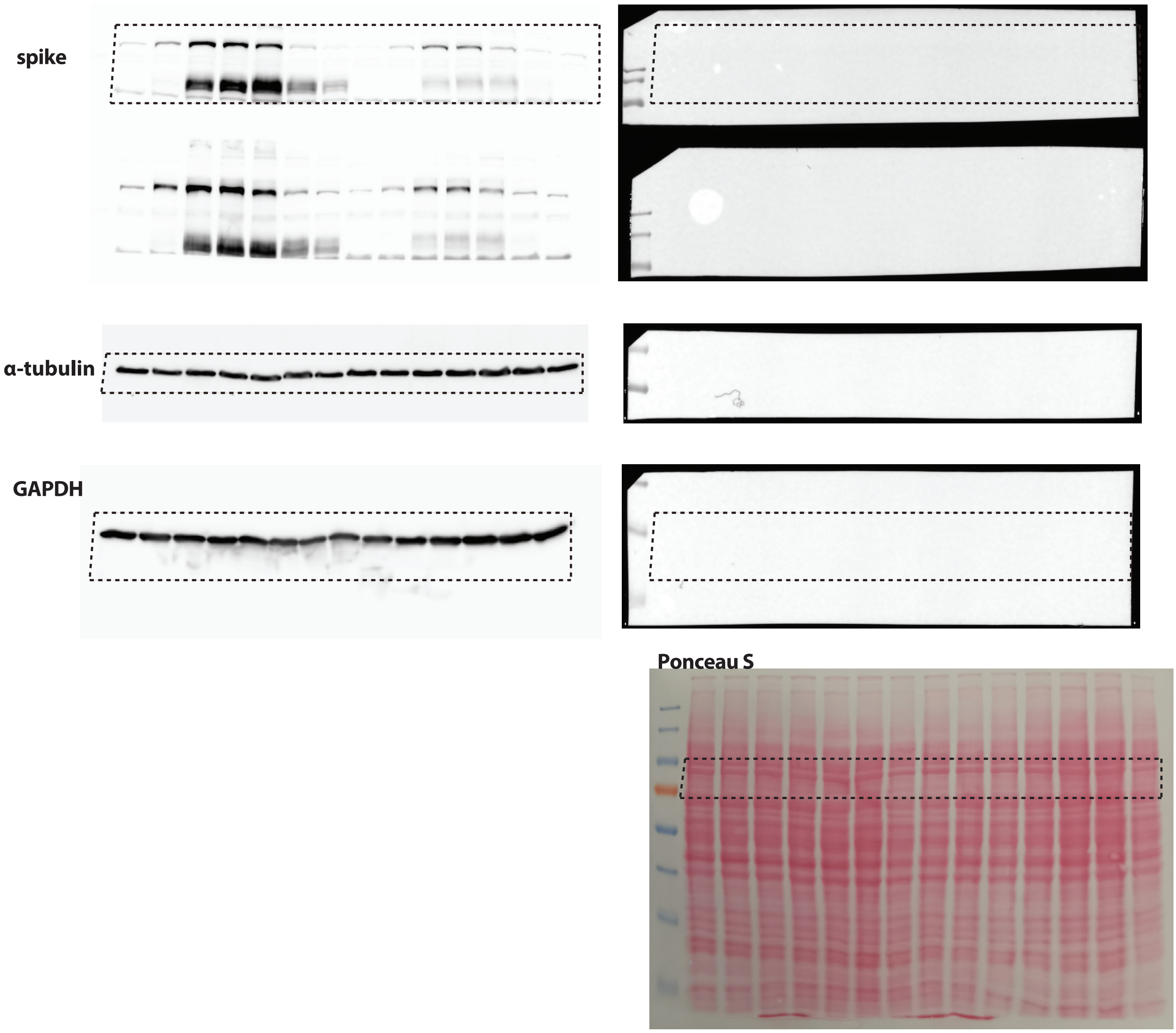

Supplementary Information Figure 7 Source Data (scans of uncropped blots 3 of 6)

e. Scans related to Extended Data Figure 2f

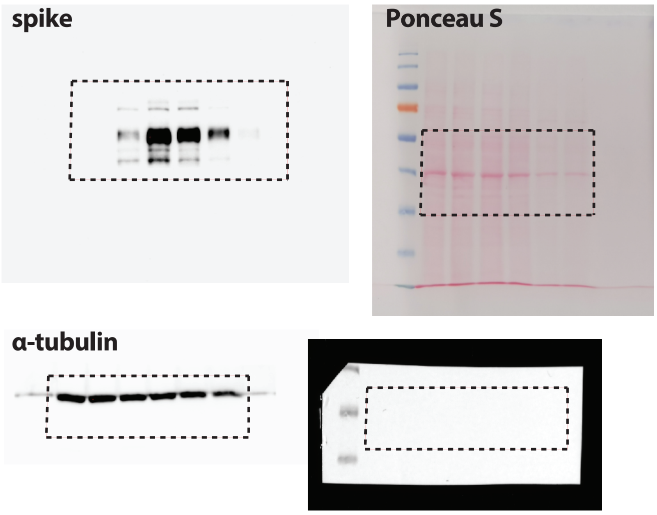

f. Scans related to Extended Data Figure 2g

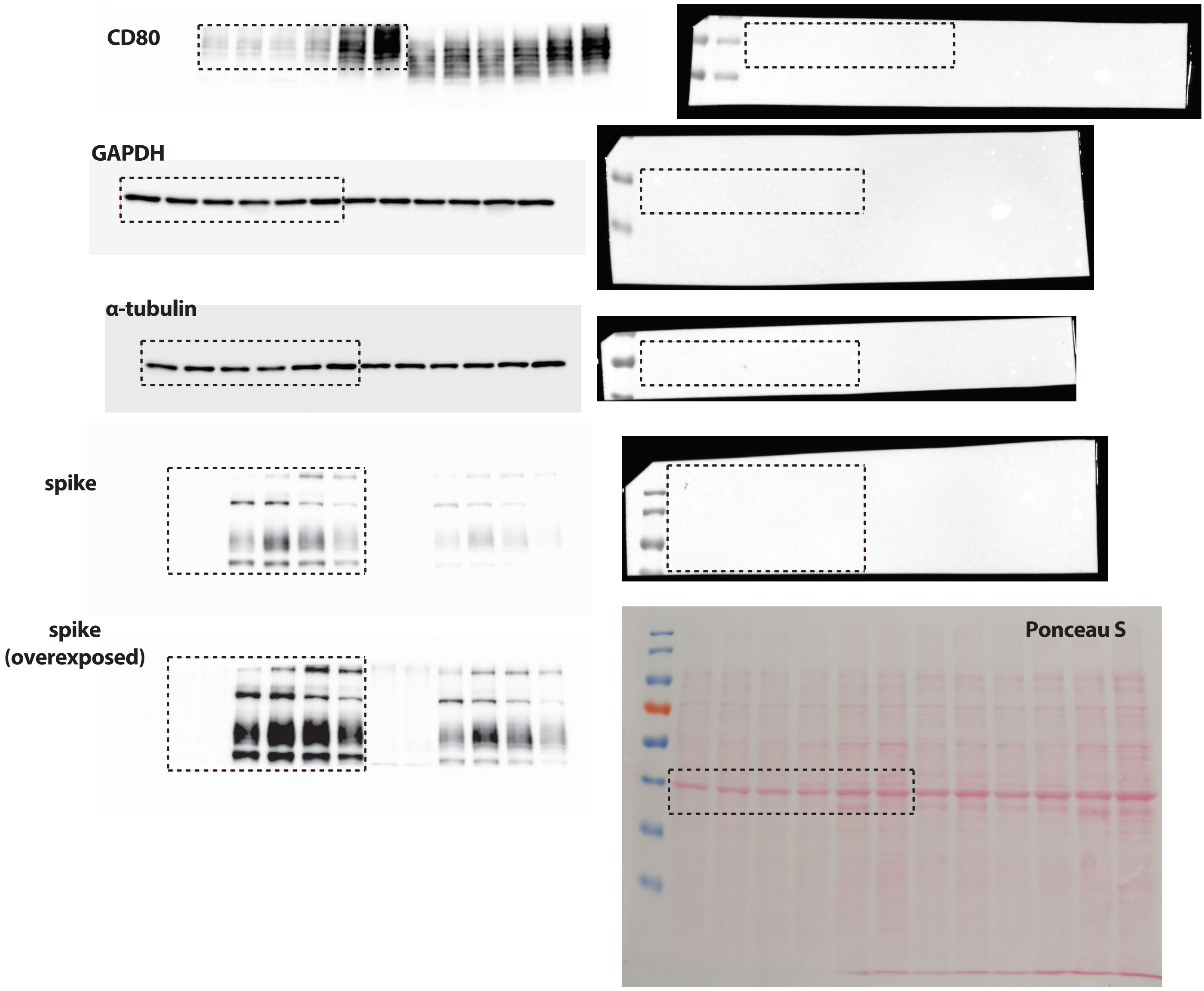

Supplementary Information Figure 7 Source Data (scans of uncropped blots 4 of 6)

g. Scans related to Extended Data Figure 2j

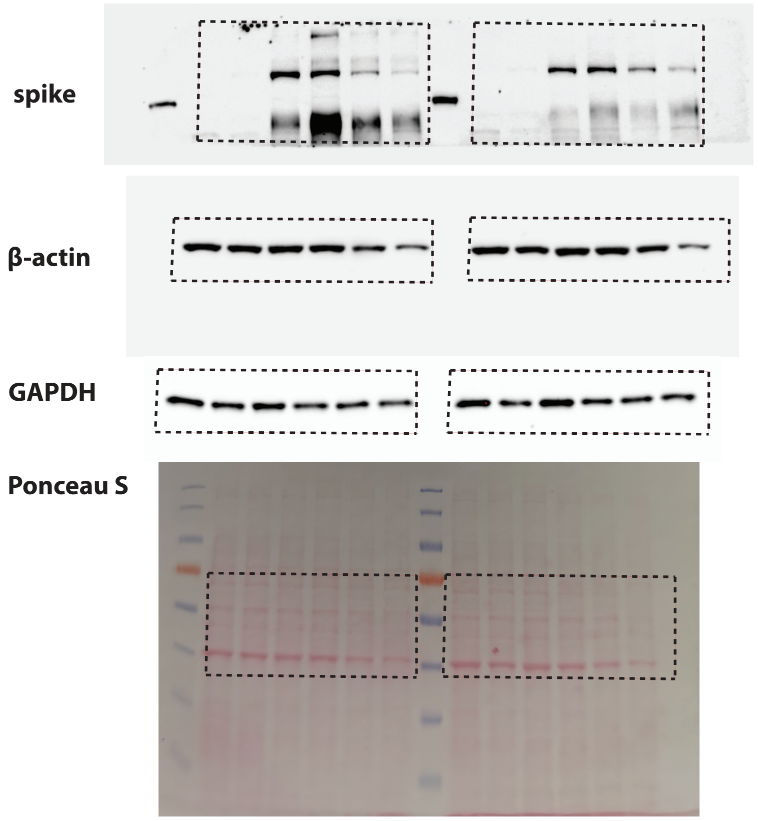

h. Scans related to Extended Data Figure 5a

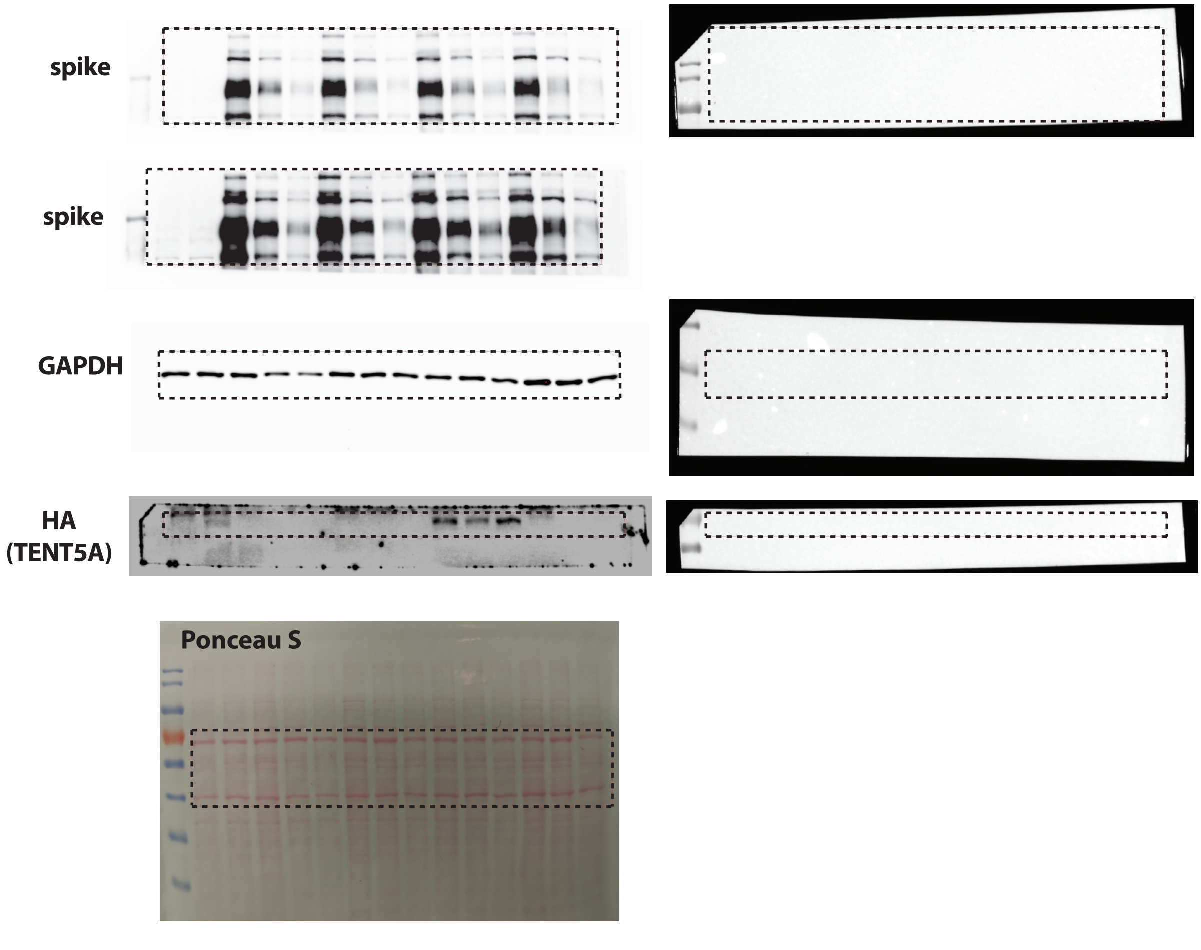

Supplementary Information Figure 7 Source Data (scans of uncropped blots 5 of 6)

i. Scans related to Extended Data Figure 5b

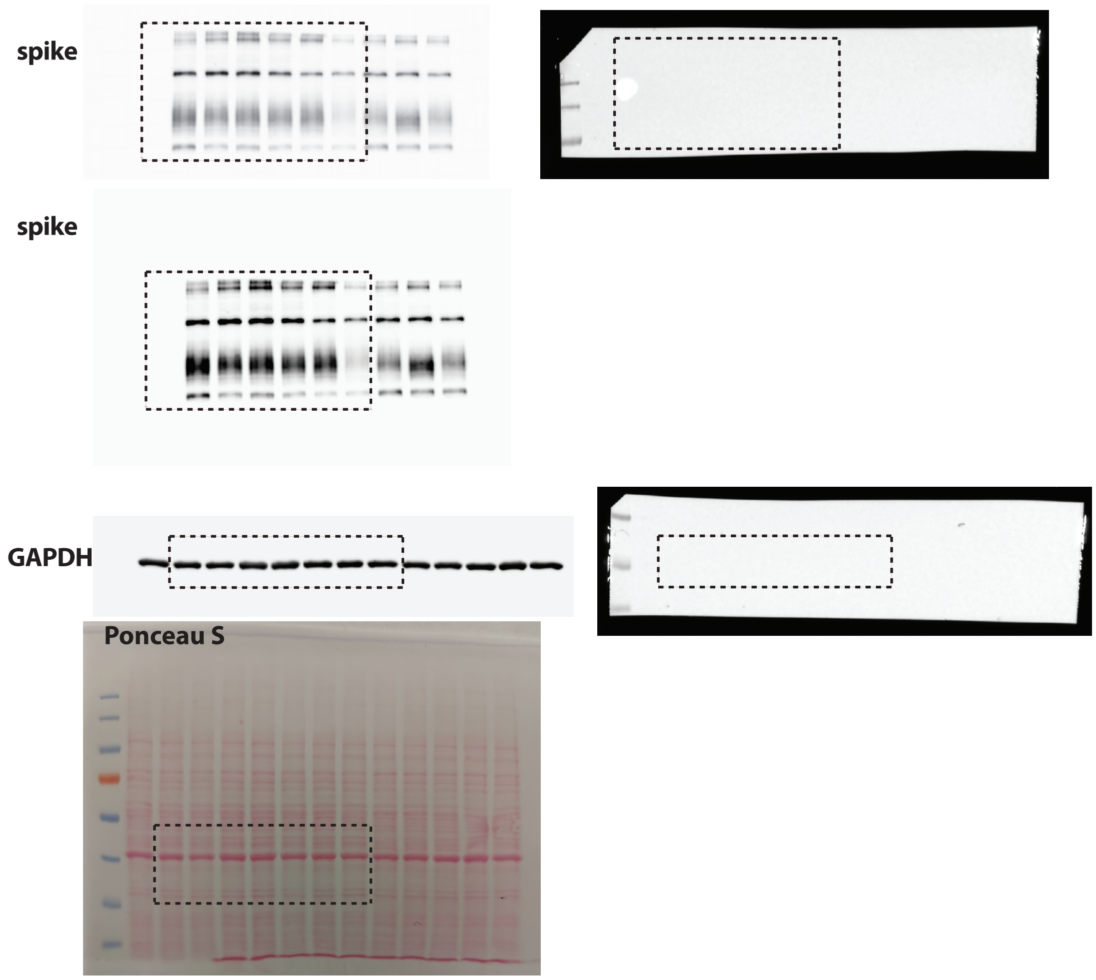

j. Scans related to Extended Data Figure 6d

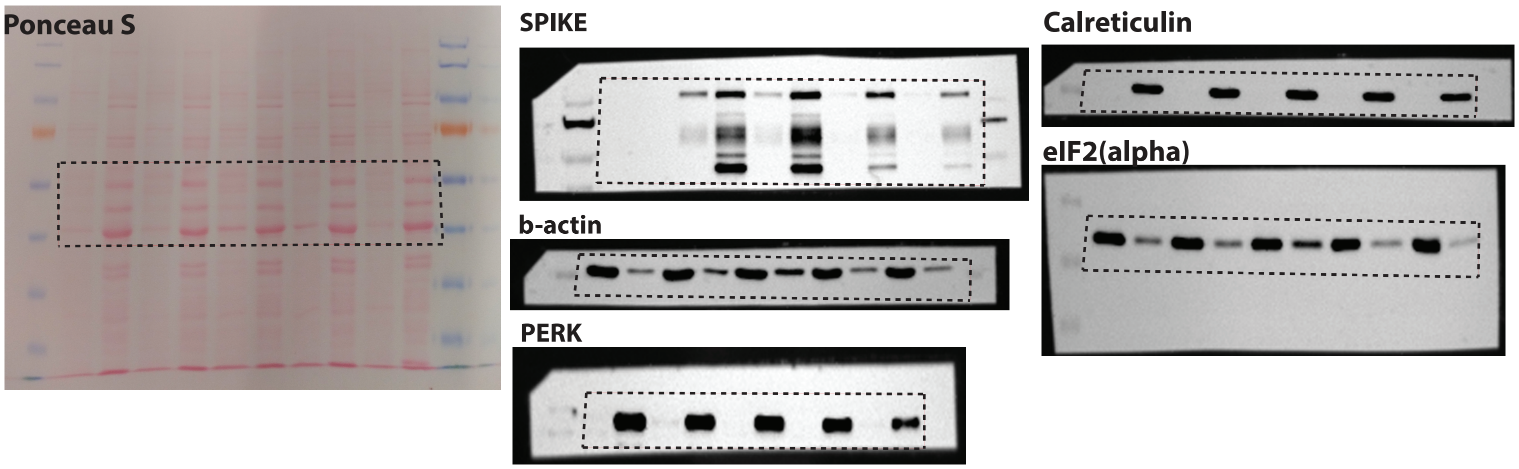

Supplementary Information Figure 7 Source Data (scans of uncropped blots 6 of 6)

k. Scans related to Extended Data Figure 6g

β-actin

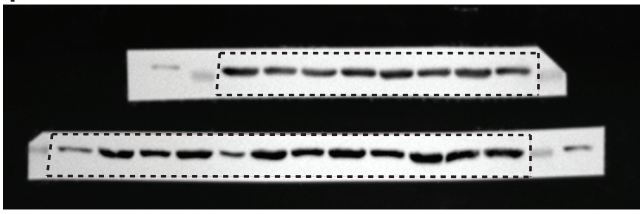

α-tubulin

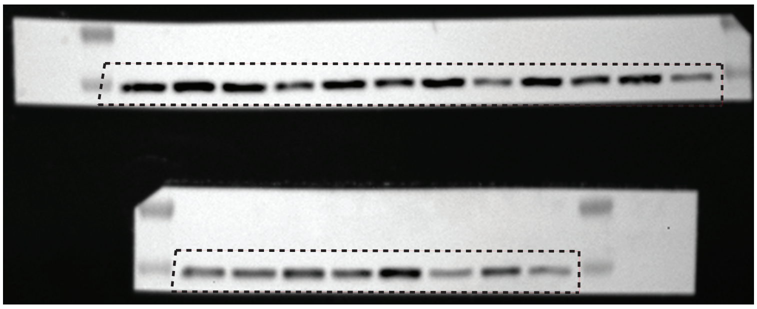

SSR1 (TRAP1)

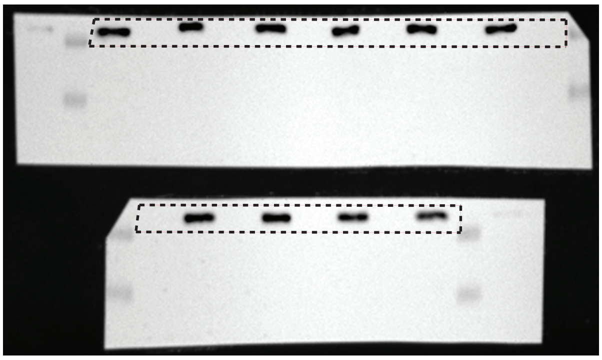

SPIKE

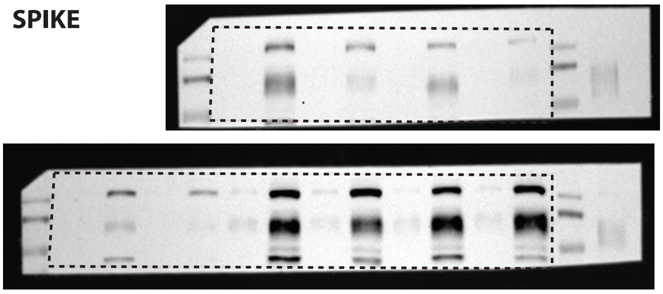

SPIKE (overexposed)

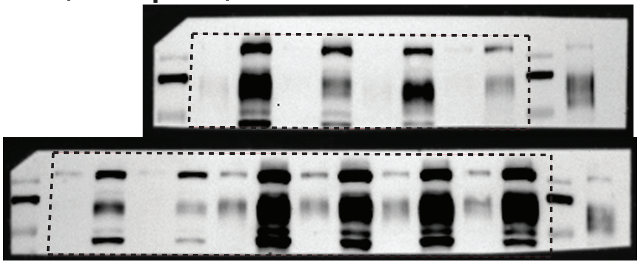

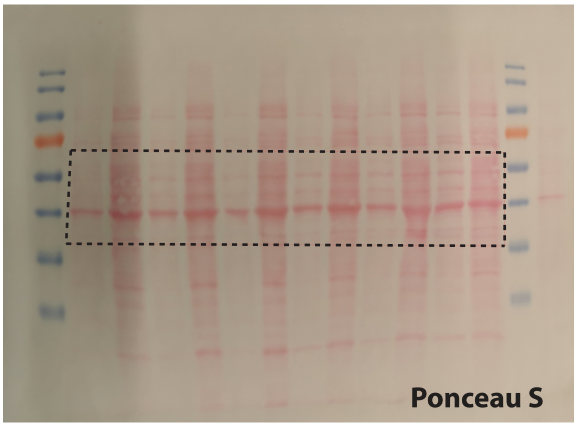

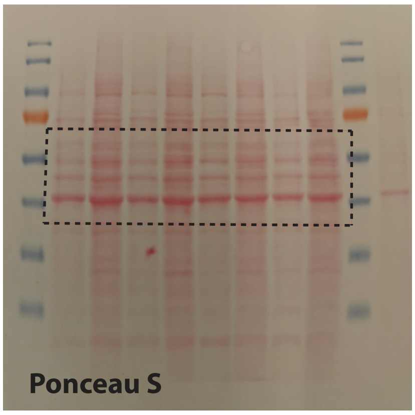

Supplement: Supplementary file 1 — This file contains Supplementary Notes 1–7, Supplementary References, descriptions of the Supplementary Tables, Supplementary Table 14 and Supplementary Figures 1–7. [file 41586_2025_8842_MOESM1_ESM.pdf]
