# Supplementary material for: Re-adenylation by TENT5A enhances efficacy of SARS-CoV-2 mRNA vaccines
Source: Nature. 2025 Apr 16;641(8064):984–92. doi: 10.1038/s41586-025-08842-1 (PMC12095053; doi:10.1038/s41586-025-08842-1)
Supplement: Supplementary file 2 — Reporting Summary [file 41586_2025_8842_MOESM2_ESM.pdf]

nature portfolio

Corresponding author(s): Seweryn Mroczek and Andrzej Dziembowski

Last updated by author(s): Jan 30, 2025

Reporting Summary

Nature Portfolio wishes to improve the reproducibility of the work that we publish. This form provides structure for consistency and transparency in reporting. For further information on Nature Portfolio policies, see our Editorial Policies and the Editorial Policy Checklist.

Statistics

For all statistical analyses, confirm that the following items are present in the figure legend, table legend, main text, or Methods section.

n/a	Confirmed	
☐	☒	The exact sample size (*n*) for each experimental group/condition, given as a discrete number and unit of measurement
☐	☒	A statement on whether measurements were taken from distinct samples or whether the same sample was measured repeatedly
☐	☒	The statistical test(s) used AND whether they are one- or two-sided<br>*Only common tests should be described solely by name; describe more complex techniques in the Methods section.*
☐	☒	A description of all covariates tested
☐	☒	A description of any assumptions or corrections, such as tests of normality and adjustment for multiple comparisons
☐	☒	A full description of the statistical parameters including central tendency (e.g. means) or other basic estimates (e.g. regression coefficient) AND variation (e.g. standard deviation) or associated estimates of uncertainty (e.g. confidence intervals)
☐	☒	For null hypothesis testing, the test statistic (e.g. *F*, *t*, *r*) with confidence intervals, effect sizes, degrees of freedom and *P* value noted<br>*Give P values as exact values whenever suitable.*
☒	☐	For Bayesian analysis, information on the choice of priors and Markov chain Monte Carlo settings
☒	☐	For hierarchical and complex designs, identification of the appropriate level for tests and full reporting of outcomes
☐	☒	Estimates of effect sizes (e.g. Cohen's *d*, Pearson's *r*), indicating how they were calculated

Our web collection on statistics for biologists contains articles on many of the points above.

Software and code

Policy information about availability of computer code

Data collection	MinKNOW (Oxford Nanopore Technologies, multiple versions, updated frequently, from 18.12 to 22.03.5) was used for data colection during ONT RNA sequencing. Guppy (Oxford Nanopore Technologies, versions 4.0.11, 4.4.1, 5.0.11, 6.0.0) or dorado (Oxford Nanopore Technologies, version 0.5.3, 0.7.0) were used for basecalling of RNA sequencing data. QuantStudio Design and Analysis Software v1.5.2 for qPCR analyses. CytExpert SRT v1.1, FACSDiva 8.3 and BD FACSuite™ software for cell sorting and cytometry.
Data analysis	Minimap 2.17; Nanopolish 0.13.2; Samtools 1.9; Guppy (versions 4.0.11, 4.4.1, 5.0.11, 6.0.0); dorado 0.5.3 and 0.7.0; R 4.1.2; R 4.2.1; STAR v.2.7.6a;cutadapt 2.10; Subread 2.0.1; Prism 6; FlowJo v10.6.1 software (BD Biosciences); Multi Gauge v3.0 (Fujifilm Life Sciences); The code for subsequence Dynamic Time Warping is deposited at https://github.com/LRB-IIMCB/DTW_mRNA-1273. The code for modified nanopolish-polya for detection of poly(A) terminal pentamer of mRNA-1273 and composite poly(A) tail of BNT162b2 is deposited at: https://github.com/LRB-IIMCB/nanopolish_mRNA-1273_BNT162b2;

For manuscripts utilizing custom algorithms or software that are central to the research but not yet described in published literature, software must be made available to editors and reviewers. We strongly encourage code deposition in a community repository (e.g. GitHub). See the Nature Portfolio guidelines for submitting code & software for further information.

Data

Policy information about availability of data

All manuscripts must include a data availability statement. This statement should provide the following information, where applicable:

- Accession codes, unique identifiers, or web links for publicly available datasets
- A description of any restrictions on data availability
- For clinical datasets or third party data, please ensure that the statement adheres to our policy

Nanopore direct RNA sequences are deposited at the European Nucleotide Archive; accession number PRJEB53190 (all sequencing datasets with corresponding ENA accession numbers are also listed in the Supplementary Information Table 13). Illumina sequencing data are deposited at the Gene Expression Omnibus; accession number GSE233059. Raw data underlying figures are provided as Source Data and Supplementary Datasets or are also available from the corresponding authors upon reasonable request.

Dynamic Time Warping script is available at https://github.com/LRB-IIMCB/DTW_mRNA-1273. Nanopolish-polya for identification of mΨCmΨAG is available at https://github.com/LRB-IIMCB/nanopolish_mRNA-1273_BNT162b2.

Research involving human participants, their data, or biological material

Policy information about studies with human participants or human data. See also policy information about sex, gender (identity/presentation), and sexual orientation and race, ethnicity and racism.

Reporting on sex and gender	Not applicable.
Reporting on race, ethnicity, or other socially relevant groupings	Not applicable.
Population characteristics	Not applicable.
Recruitment	Not applicable.
Ethics oversight	Not applicable.

Note that full information on the approval of the study protocol must also be provided in the manuscript.

Field-specific reporting

Please select the one below that is the best fit for your research. If you are not sure, read the appropriate sections before making your selection.

☒ Life sciences ☐ Behavioural & social sciences ☐ Ecological, evolutionary & environmental sciences

For a reference copy of the document with all sections, see nature.com/documents/nr-reporting-summary-flat.pdf

Life sciences study design

All studies must disclose on these points even when the disclosure is negative.

Sample size	No formal sample size calculations were performed a priori. For in vivo animals experiments sample sizes were determined based on established practices in similar published studies and the 3Rs principles (Replacement, Reduction, Refinement) for ethical animal research (Dell et al., ILAR J. 2002). We used minimum numbers necessary to achieve statistical significance while maintaining scientific rigor, based on our previous experience and comparable studies (Li et al., Nat Immunol 2022).
Data exclusions	There was no formal criteria of sample exlusions however samples with clear technical failures during sequencing process were excluded from analysis.
Replication	Samples were produced in biological duplicates or triplicates with exception of mRNA-1273 treatment of A549 cells and viability assays, which were repeated once. The exact number of biological replicates used for statistical anaysis is stated for every single experiment. As described above, samples with clear technical failures during processing or data collection were excluded from analyses this applies to nanopore sequencing and ELISA measurements).
Randomization	Mice of different genotypes were assigned with individual numerical tags in the database and they were used for the tissue collection and throughout subsequent processing as the only identifiers.
Blinding	Individual mice had an assigned numerical tag and mice with different genotypes were indistinguishable by the experimenter (except for Tent5a(-/-) mice which have distinguishable phenotype). However, vaccination is a routine procedure and therefore the knowledge about the genotype of mice at the time of vaccination was highly unlikely to affect the result of immunization. Blinding was not relevant for the experiments performed on cultured cells.

Reporting for specific materials, systems and methods

We require information from authors about some types of materials, experimental systems and methods used in many studies. Here, indicate whether each material, system or method listed is relevant to your study. If you are not sure if a list item applies to your research, read the appropriate section before selecting a response.

Materials & experimental systems

n/a	Involved in the study	
☐	☒	Antibodies
☐	☒	Eukaryotic cell lines
☒	☐	Palaeontology and archaeology
☐	☒	Animals and other organisms
☒	☐	Clinical data
☒	☐	Dual use research of concern
☒	☐	Plants

Methods

n/a	Involved in the study	
☒	☐	ChIP-seq
☐	☒	Flow cytometry
☒	☐	MRI-based neuroimaging

Antibodies

Antibodies used

The antibodies used in this study are listed in Supplementary Table Antibodies as well as in the methods section.
Anti-SARS-CoV-2 Spike Glycoprotein S1 antibody (Abcam, ab275759, 1:2500), Anti-SARS-CoV-2 Spike Glycoprotein antibody (Abcam, ab272504, 1:2500), Anti-GAPDH (Novus Biologicals, NB300-327, 1:5000), Anti-Tubulin (Millipore, mAb DM1A, CP06, 1:5000), Anti-Actin (Cell Signaling; 13E5, 4970, 1:5000), Anti-Actinin (Cell Signaling; 3134, 1:5000), Anti-PDI (Cell Signaling, C81H6, 3501, 1:2500), Goat Anti-Mouse IgG, H&L Chain Specific Peroxidase Conjugate (Millipore Cat# 401215, 1:5000), Goat Anti-Rabbit IgG, H&L Chain Antibody, Peroxidase Conjugated (Millipore Cat # 401393, 1:5000), Anti-CD80 (E3Q9V, Cell Signaling, 15416, 1:2500), Anti-GRP94 (Santa Cruz, H-212, sc-11402, 1:2500), Anti-eIF2alpha (Cell Signaling, 9722, 1:5000), Anti-Calreticulin (Cell Signaling, D3E6, 12238, 1:2500), Anti-PERK (Cell Signaling, C33E10, 3192, 1:2500), Anti-HA (Roche/Sigma, 12CA5, 11583816001, 1:2500), Anti-SSR1 (Abcam, ab240562, 1:2500).
PerCp-Cy™5.5 Rat anti-mouse CD45 IgG2b κ 30-F11 (BD Biosciences; 550994, 1:50); BV786 Rat anti-mouse CD11b Rat IgG2b, κ M1/70 (BD Biosciences; 740861, 1:50); PE-Cy7 Rat anti-mouse F4/80 IgG2a, κ BM8 (eBioscience; 25-4801-82, 1:50); BV605 Rat anti-mouse I-A/I-E IgG2b, κ M5/114.15.2 (BD Biosciences; 563413, 1:50); PE American hamster anti-mouse CD11c IgG N418 (eBioscience; 12-0114-82, 1:50); APC-Fire™ Mouse anti-mouse CD64 IgG1, κ X54-5/7.1 (BioLegend; 139333, 1:50).
PerCp-Cy5.5 Mouse anti-human CD1c IgG1, κ L161 (BioLegend; 331512, 1:300); PE-Cy7 Mouse anti-human CD11c IgG1, κ Bu15 (BioLegend; 337216, 1:300); PE Mouse anti-human CD83 IgG1, κ HB15e (BioLegend; 305308, 1:300); APC Mouse anti-human CD86 IgG2b, κ IT2.2 (BioLegend; 305412, 1:300); APC-Cy7 Mouse anti-human HLA-DR IgG2a, κ L243 (BioLegend; 307618, 1:300); AlexaFluor 700 Mouse anti-human CD14 IgG2a, κ M5E2 (BioLegend; 301822, 1:300); FITC Mouse anti-human CD83 IgG1, κ HB15e (BioLegend; 305306, 1:300); PerCp-Cy5.5 Mouse anti-human CD163 IgG1, κ GHI/61 (BD Biosciences; 563887, 1:300).

Validation

Antibodies specific for the required antigens or epitopes were purchased from commercial vendors and specific clones were chosen based on the literature, our own pilot studies, or by the product data sheets provided by companies.

1. https://www.abcam.com/en-pl/products/primary-antibodies/sars-cov-2-spike-glycoprotein-s1-antibody-ab275759
2. https://www.abcam.com/en-us/products/primary-antibodies/sars-cov-2-spike-glycoprotein-antibody-coronavirus-ab272504
3. https://www.novusbio.com/products/gapdh-antibody_nb300-327
4. https://www.merckmillipore.com/PL/pl/product/Anti-Tubulin-Mouse-mAb-DM1A,EMD_BIO-CP06
5. https://www.cellsignal.com/products/primary-antibodies/b-actin-13e5-rabbit-mab/4970
6. https://www.cellsignal.com/products/primary-antibodies/a-actinin-antibody/3134
7. https://www.cellsignal.com/products/primary-antibodies/pdi-c81h6-rabbit-mab/3501
8. https://www.merckmillipore.com/PL/pl/product/Goat-Anti-Mouse-IgG-HL-Chain-Specific-Peroxidase-Conjugate,EMD_BIO-401215
9. https://www.merckmillipore.com/PL/pl/product/Goat-Anti-Rabbit-IgG-H-L-Chain-Specific-Peroxidase-Conjugate,EMD_BIO-401393
10. https://www.cellsignal.com/products/primary-antibodies/cd80-e3q9v-rabbit-mab/15416
11. https://www.scbt.com/p/grp-94-antibody-h-212?srsltid=AfmBOop9l8MWVRcLTD1duekLoijuhp4Pqn3lAySw5EEA5teQuEdSc2Jv
12. https://www.cellsignal.com/products/primary-antibodies/eif2a-antibody/9722?srsltid=AfmBOorzKh54bldneFWrmX9lP-mm11ve7e_1KGZQwNeVLqWNT-ciQ3Xe
13. https://www.cellsignal.com/products/primary-antibodies/calreticulin-d3e6-xp-rabbit-mab/12238?srsltid=AfmBOorsV4HFZ8LztLlsDJsJ1gMRxxDBSdCPlVRKuUxoiavjP7EwNEBd
14. https://www.cellsignal.com/products/primary-antibodies/perk-c33e10-rabbit-mab/3192?srsltid=AfmBOorBw-IQ6XaHYoh_eBrhglp2LCoIqhpGcj9k1PXYMN70vVbqVO3o
15. https://www.sigmaaldrich.com/PL/pl/product/roche/roahaha?utm_source=google&utm_medium=cpc&utm_campaign=8809292844&utm_content=90342175164&gad_source=1&gclid=CjwKCAiA-ty8BhA_EiwAkyoa374iD4N-cIAxqbFwFus9xDFas9s0LvtnJ2cchp4gPBGnjIwwCbps5xoCv2kQAvD_BwE
16. https://www.abcam.com/en-us/products/primary-antibodies/trap-alpha-trapa-antibody-ab240562
17. https://www.bdbiosciences.com/en-us/products/reagents/flow-cytometry-reagents/research-reagents/single-color-antibodies-ruo/percp-cy-5-5-rat-anti-mouse-cd45.55099411.
18. https://www.bdbiosciences.com/en-us/products/reagents/flow-cytometry-reagents/research-reagents/single-color-antibodies-ruo/bv786-rat-anti-cd11b.740861
19. https://www.thermofisher.com/antibody/product/F4-80-Antibody-clone-BM8-Monoclonal/25-4801-82
20. https://www.bdbiosciences.com/en-us/products/reagents/flow-cytometry-reagents/research-reagents/single-color-antibodies-ruo/bv605-rat-anti-mouse-i-a-i-e.563413

21. https://www.thermofisher.com/antibody/product/CD11c-Antibody-clone-N418-Monoclonal/12-0114-82
22. https://www.biolegend.com/fr-ch/products/apc-fire-750-anti-mouse-cd64-fcgammari-antibody-21731
23. https://www.biolegend.com/nl-be/products/percp-anti-human-cd1c-antibody-5181
24. https://www.biolegend.com/de-at/products/pe-cyanine7-anti-human-cd11c-antibody-6129
25. https://www.biolegend.com/en-gb/products/pe-anti-human-cd83-antibody-681?GroupID=BLG10102
26. https://www.biolegend.com/en-gb/products/apc-anti-human-cd86-antibody-2864?GroupID=BLG11941
27. https://www.biolegend.com/en-ie/products/apc-cyanine7-anti-human-hla-dr-antibody-2863?GroupID=BLG1
28. https://www.biolegend.com/fr-ch/products/alexa-fluor-700-anti-human-cd14-antibody-3397?GroupID=BLG4805
29. https://www.biolegend.com/en-gb/products/fitc-anti-human-cd83-antibody-680
30. https://www.bdbiosciences.com/en-us/products/reagents/flow-cytometry-reagents/research-reagents/single-color-antibodies-ruo/percp-cy-5-5-mouse-anti-human-cd163.563887

Eukaryotic cell lines

Policy information about cell lines and Sex and Gender in Research

Cell line source(s)

The primary Bone Marrow Derived Macrophages cell cultures were established from the bone marrow monocytes isolated from Tent5a(Flox/Flox)/Tent5c(-/-) and wild-type mice as desribed previously (Liudkovska et al, Science Adv, 2022). Bone marrows were isolated from femurs of mice of both sex, and, in all cases, established from the material mixed from multiple individuals (siblings of the same sex) to obtain number of cells sufficient for subsequent analyses. The sex of animals used as a source of bone marrow cells was not considered in the further analyses.

Human monocyte-derived macrophages (hMDMs) were isolated from buffy coats obtained commercially from the Regional Blood Centre in Warsaw, Poland. All donors were 18 to 45 years old healthy males.

HEK293 Flp-In T-REx (R78007, Thermo Fisher Scientific) cell line with conditional knock-down of CNOT1 was generated in this study, as described in Methods section. HEK293 Flp-In T-REx (HEK293T) was obtained from ATCC (ATCC; CRL-3216). A549 cell line was obtained from Sigma-Aldrich (#86012804-1VL).

Authentication

None of the cell lines were authenticated.

Mycoplasma contamination

All cell lines tested negative for mycoplasm contamination by PCR.

Commonly misidentified lines
(See ICLAC register)

No commonly misidentified lines were used in this study.

Animals and other research organisms

Policy information about studies involving animals; ARRIVE guidelines recommended for reporting animal research, and Sex and Gender in Research

Laboratory animals

All mice lines except for CD11+ CRE were generated by the CRISPR/Cas9-based method in the Genome Engineering Unit (https://crisprmice.eu/) at the International Institute of Molecular and Cell Biology in Warsaw, and described previously (double knockout Tent5a(Flox/Flox)/Tent5c(-/-) in Liudkovska et al., Science Adv. 2022 and Tent5a(-/-) in Gewartowska et al., Cell Rep, 2021), with the exception of dTag-Tent5a mouse line which was newly generated. CD11+ CRE mouse line was purchased from Jacksons Laboratory (JAX #008068).
Mice of both sex were used for the immunization experiments at age 6-14 weeks.
All mice were bred at the animal house of Faculty of Biology, University of Warsaw. Mice were maintained in conventional conditions in open polypropylene cages filled with wood chip bedding (Rettenmaier) . Environment was enriched with nest material and paper tubes. Mice were fed at libutum with standard laboratory diet (Labofeed B, Morawski). Humidity in the rooms was kept at 55% ± 10%, temperature at 22oC ± 2oC, at least 15 air changes per hour, light regime set at 12h/12h (lights on from 6:00 to 18:00).

Wild animals

No wild animals were used in the study.

Reporting on sex

Mice of both sex were used for the immunization experiments at age 6-14 weeks, with equal or similar ratios of both sex in directly compared groups. Sex of the subjects was not considered in the immunization experiments, only the genotype. Sex of mice used for the immunization experiments is provided in the Source Data (where available)

Field-collected samples

No field collected samples were used in the study.

Ethics oversight

All procedures with animals were approved by were approved by the II Local Ethical Committee in Warsaw (approval numbers: WAW2/71/2021, WAW2/129/2021, WAW2/95/2022, WAW2/127/2022, WAW2/007/2023) with the requirements of the EU (Directive 2010/63/EU) and Polish (Act number 266/15.01.2015) legislation.

Note that full information on the approval of the study protocol must also be provided in the manuscript.

Plants

Seed stocks	No plants were used in this study.
Novel plant genotypes	No plants were used in this study.
Authentication	No plants were used in this study.

Flow Cytometry

Plots

Confirm that:

☒ The axis labels state the marker and fluorochrome used (e.g. CD4-FITC).

☒ The axis scales are clearly visible. Include numbers along axes only for bottom left plot of group (a 'group' is an analysis of identical markers).

☒ All plots are contour plots with outliers or pseudocolor plots.

☒ A numerical value for number of cells or percentage (with statistics) is provided.

Methodology

Sample preparation	Cells were isolated from muscles, differentiated in cultures in vitro or isolated from buffy coats as described in the methods section under the sections entitled Flow Cytometry Analysis.
Instrument	CytoFlex SRT cell sorter (Beckman Coulter); Fortessa X20 and FACSLyric analyzer (BD Biosciences).
Software	CytExpert SRT v1.1 Software for the CytoFLEX Platform; FACSDiva 8.3 software for Fortessa X20. BD FACSuite™ for FACSLyric analyzer.
Cell population abundance	Purity of post-sort fractions for cells of interest was determined by sorting 1000 cells and re-acquiring at least 50% of each sample. The purity of sorted cells was at least 85% of phenotype intended for enrichment.
Gating strategy	Cell populations are described in the relevant figures and legends. Detailed gating strategy is described in Extended Data Figure 8 and in the methods section under the title Flow Cytometry Analysis.

☒ Tick this box to confirm that a figure exemplifying the gating strategy is provided in the Supplementary Information.
